# Application of Enantioselective Sulfur Ylide Epoxidation to a Short Asymmetric Synthesis of Bedaquiline, a Potent Anti-Tuberculosis Drug

**DOI:** 10.1021/acs.orglett.3c01286

**Published:** 2023-06-07

**Authors:** Maryam Bashir, Muhammad Arshad, Robina Begum, Varinder K. Aggarwal

**Affiliations:** † School of Chemistry, University of Bristol, Cantock’s Close, Bristol BS8 1TS, U.K.; ‡ Centre for Organic Chemistry, School of Chemistry, University of the Punjab, Lahore 54590, Pakistan; § Institute of Chemistry, The Islamia University of Bahawalpur, Bahawalpur 63100, Pakistan

## Abstract

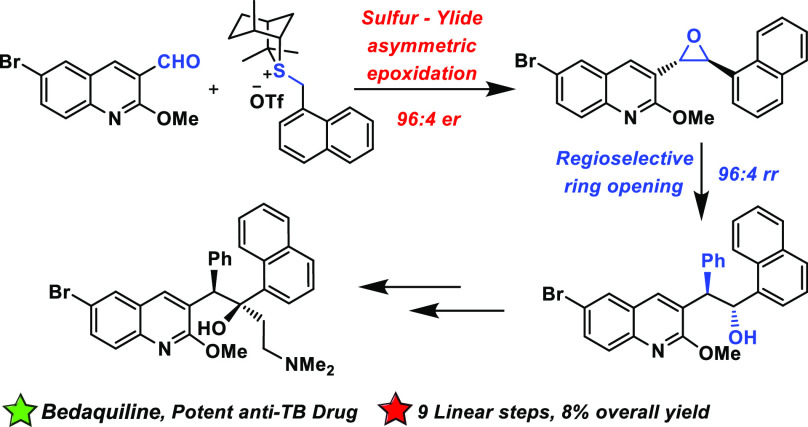

A highly selective asymmetric synthesis of a potent anti-TB drug (−)-bedaquiline is accomplished using sulfur ylide asymmetric epoxidation, employing (+)-isothiocineole as an inexpensive and readily available chiral sulfide. Excellent enantioselectivity (er 96:4) and diastereoselectivity (dr 90:10) were obtained for the construction of the key diaryl epoxide, which was subsequently subjected to a highly regioselective ring opening (96:4). The synthesis was completed in nine steps starting from commercially available aldehyde in 8% overall yield.

Tuberculosis (TB), a bacterial infection primarily caused by *Mycobacterium tuberculosis*, has been and continues to be a major global health concern, particularly in low- and middle-income countries.^1^ Despite the fact that TB is curable, almost one-quarter of the world’s population has been infected with latent TB, rendering it one of the leading causes of death.^2^ The percentage of new TB cases has increased significantly due to multidrug resistance (MDR) TB and extensive-drug resistance (XDR) TB, making it one of the prime challenges in medicinal chemistry. The first MDR tuberculosis drug,^3^ (−)-bedaquiline (BDQ) **(−)-1** (TMC 207, R207910, branded as Sirturo), was introduced in 2012.^4^ The compound contains two stereogenic centers, but only the 1*R*/2*S* enantiomer is effective against *M. tuberculosis*.

BDQ inhibits both drug-sensitive and drug-resistant bactericidal growth through inhibition of the proton pump of adenosine triphosphate (ATP) synthase. Its high lipophilicity^5,6^ has fueled the development of more water-soluble variants, e.g., **1a**(7) (Figure 1).

**Figure 1:**
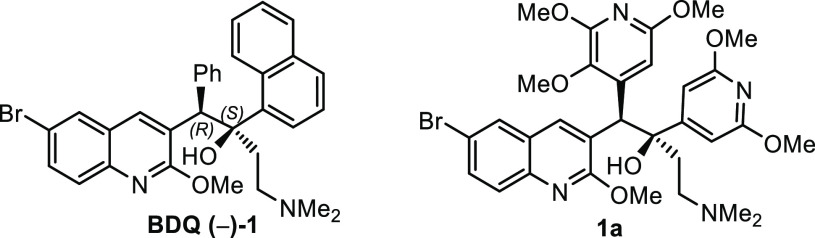
Bedaquiline **(−)-1** and water-soluble variant **1a**.

Several stereoselective syntheses of BDQ **(−)-1** have been reported.^3,8−10^ The first catalytic asymmetric synthesis was reported by Shibasaki^8^ in 12 steps (longest linear sequence) using an enantioselective proton migration as the key step (Scheme 1a). Chandrasekhar et al.^9^ reported the total synthesis of BDQ **(−)-1** also in 12 steps using a Sharpless asymmetric epoxidation (Scheme 1b). Recently, a direct diastereoselective synthesis was reported using a chiral base in the deprotonation step giving a 90:10 mixture of diastereoisomers but as a racemic mixture, which was separated by chiral supercritical fluid chromatography (SFC) to access the desired enantiomer (1*R*,2*S*)-BDQ^10^ (Scheme 1c). Herein, we report a nine-step asymmetric synthesis of bedaquiline **(−)-1** employing the sulfur ylide-mediated asymmetric epoxidation as a key step followed by a regioselective ring opening of the resulting epoxide (Scheme 1d).

**Scheme 1:**
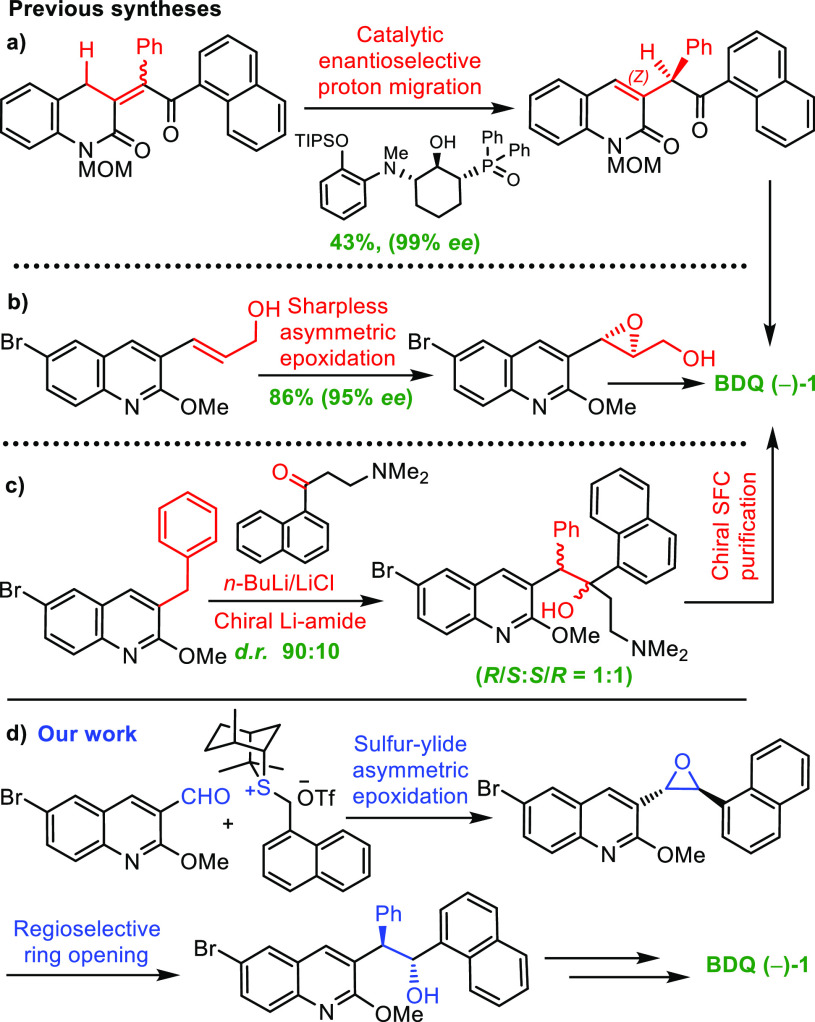
Previous Syntheses and Our Synthesis of Bedaquiline **(−)-1** (a) Shibasaki’s catalytic asymmetric synthesis (overall yield of 5%), (b) Chandrasekhar’s catalytic asymmetric synthesis (overall yield of 12%), (c) Lubanyana’s diastereoselective but racemic synthesis (overall yield of 13%), and (d) this work (sulfur ylide-mediated asymmetric synthesis).

We considered accessing BDQ **(−)-1** from either a trisubstituted epoxide **2** or a disubstituted epoxide **3**, each of which could potentially be obtained through an asymmetric sulfur ylide-mediated epoxidation reaction.^11,12^ The most direct route involved trisubstituted epoxide **2**, which could be obtained through coupling of sulfonium salt **4** with ketone **5**. However, there was no precedent for controlling the diastereo- and enantioselectivity in the formation of such a trisubstituted epoxide.^13^ Instead, we considered going via disubstituted epoxide **3**, which had much greater precedent, but this route required a regioselective ring opening of a diaryl epoxide, which again had little precedent. The key epoxide **3** could be prepared by the coupling of sulfonium salt **7** to aldehyde **8** (Scheme 2). To explore the regioselectivity in the ring opening of diaryl epoxide **3**, we targeted 1,2-disubstituted epoxide **13** for testing our methodology. This was prepared by the reaction of the known 2-methoxyquinoline-3-carboxaldehyde **11**(14,15) with sulfonium salt **12**.^16^ Treatment of sulfonium salt **12** and aldehyde **11** with KOH in a 9:1 MeCN/H_2_O solvent gave the corresponding 1,2-disubstituted epoxide **13** in high yield (88%) and 98:2 *trans*:*cis* ratio (scheme in Table 1).^16^

**Scheme 2:**
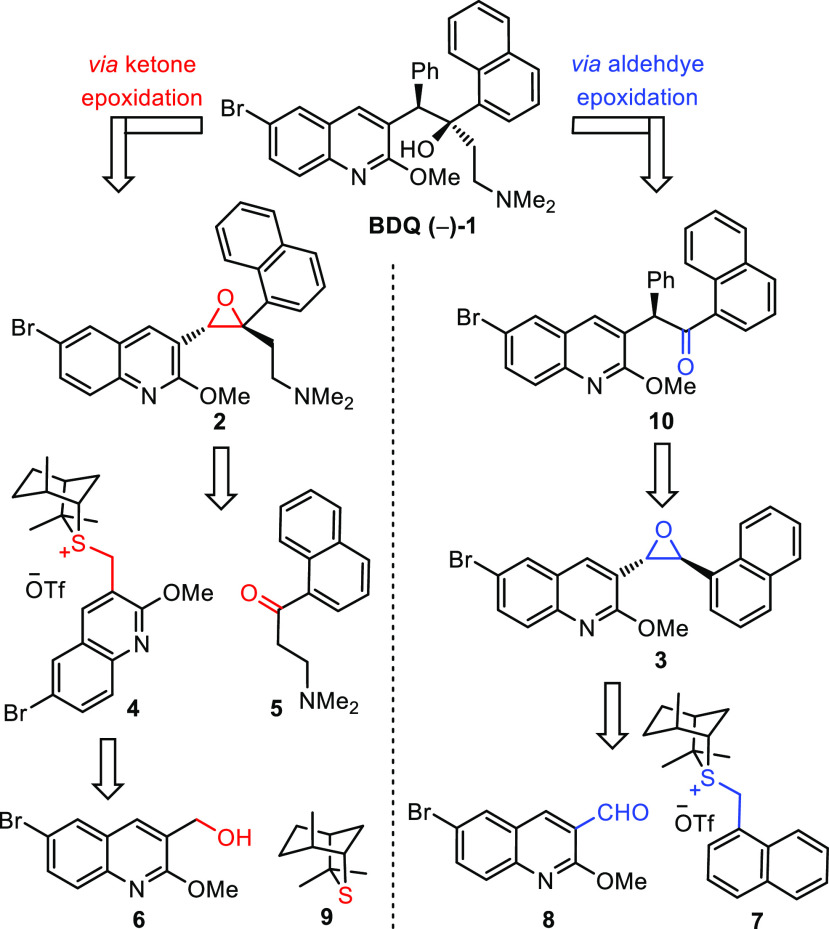
Retrosynthetic Analysis of BDQ **(−)-1**

**Table 1:**
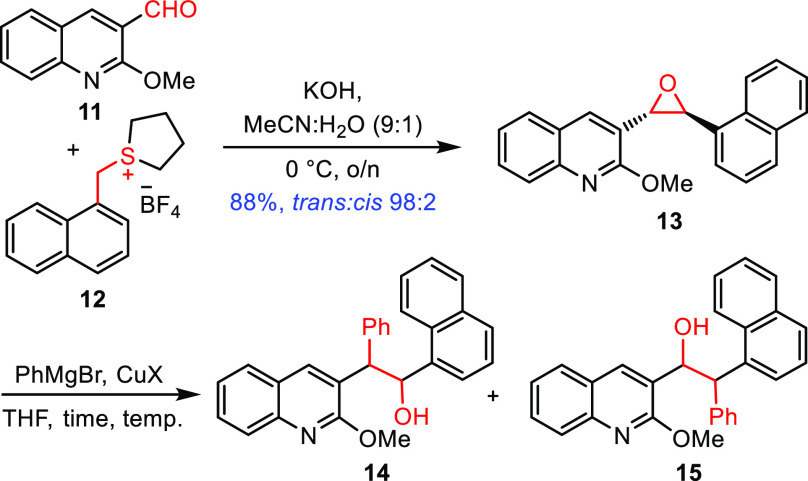
Optimization of the Regioselective Ring Opening of Epoxide **13**

entry	temp (°C)	PhMgBr (equiv)	CuX (equiv)	yield (%)^a^	**14**:**15**	RSM (%)^a^
1	–40	2.5	CuBr·SMe_2_ (0.25)	2	–	92
2	–78	5 (PhLi)	CuI (2.5)	–	–	–
3	–40	2.5	CuI (0.25)	41	1.1:1	30
4	–40	5	CuCl (2.5)	30	1.3:1	47
5	–40	10	CuCN (5)	64	3:1	–
6^b^	–78	10	CuCN (5)	66	5:1	–

a The yield (**14** + **15**) was determined with NMR by adding dibromomethane as an internal standard. RSM = recovered starting material.

b Slow addition of Grignard reagent.

With epoxide **13** in hand, we investigated its regioselective ring opening using PhMgBr in the presence of different copper salts and under different conditions (Table 1).^13,17^ Of the copper salts examined, CuCN was found to be optimal, and using an excess of the Grignard reagent at a low temperature was found to give a 5:1 ratio of regioisomers **14** and **15** in favor of the desired regioisomer **14** (entry 6). In particular, slow addition of the epoxide at −78 °C was important for achieving high regioselectivity, presumably by limiting exotherms and maintaining a constant (low) temperature. Although difficult to rationalize, it is possible that the *o*-MeO group on the quinoline promotes ring opening by either coordinating to the organometallic and guiding it into the adjacent position, or by weakening the C–O bond through donation, or both. Having established that regio-control could be achieved, we began with the synthesis of bedaquiline itself.

The stereochemistry of the key epoxide could be controlled by the choice of the chiral sulfide enantiomer employed. (−)-Isothiocineole **(−)-9** is easy to access in enantiopure form because it is derived from (+)-limonene, which is itself available in 99:1 er, whereas (+)-isothiocineole **(+)-9** requires a low-temperature recrystallization to upgrade the ee, because (−)-limonene is available in only 90:10 er.^18^ Even though the use of (−)-isothiocineole **(−)-9** would be expected to lead to the opposite enantiomer (1*S*,2*R*) of (+)-bedaquiline **(+)-1** on the basis of the established model, we elected to test out all of the chemistry on this more readily available chiral sulfide enantiomer.

The synthesis began with the preparation of known 6-bromo-2-methoxyquinoline-3-formaldehyde **8** from commercially available 6-bromo-2-chloroquinoline-3-formaldehyde **17**.^14,15^ (−)-Isothiocineole **(−)-9** was treated with 1-(bromomethyl)naphthalene **16** in a two-phase mixture of 1-butanol and an aqueous solution of LiOTf, affording sulfonium salt **(−)-7** in 68% yield.^16,19^ Sulfonium salt **(−)-7** was reacted with aldehyde **8** in the presence of KOH in a 9:1 MeCN/H_2_O solvent, which gave epoxide **(+)-3** in 88% yield with excellent dr (99:1) but an unexpectedly low 72:28 er (Table 2, entry 1).

**Table 2:**
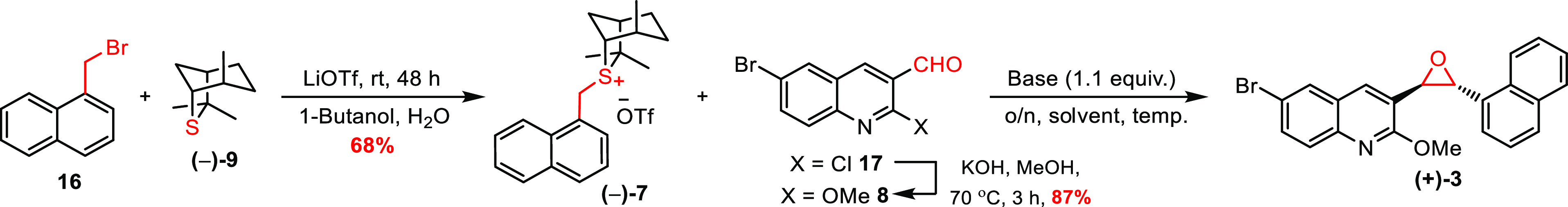
Optimization of the Enantioselectivity of Key Epoxide **(+)-3**

entry	base	scale of salt **(−)-7** (mmol)	concentration of salt **(−)-7** (M)	solvent	temp (°C)	yield (%)^a^	dr (*trans*:*cis*)	er
1	KOH	0.1	0.1	MeCN/H_2_O (9:1)	0	88	99:1	72:28
2	KOH	0.1	0.1	MeCN/^*t*^BuOH (15:1)	0	86	96:4	55:49
3	KOH	0.1	0.1	MeCN/H_2_O (4:1)	0	92	99:1	75:25
4	KOH	0.1	0.1	MeCN/^*t*^BuOH (1:5)	0	67	75:25	67:33
5	KOH	0.1	0.1	MeCN/H_2_O (2:1)	0	88	98:2	77:23
6	LiHMDS	0.5	0.1	THF	–78	46	90:10	85:15
7	LiHMDS	0.5	0.045	THF	–78	53	88:12	96:4
8	LiHMDS^b^	2.5	0.040	THF	–78	80	90:10	96:4

a Isolated yield of *trans* epoxide.

b The addition of the base was slow using a syringe pump (0.01 mL/min).

The unexpected low enantioselectivity clearly required further optimization, and we were guided by the mechanism and the factors that control enantioselectivity. The ylide preferentially adopts conformation **7A** and reacts on the front face to give betaine **7A-I**, which undergoes bond rotation and ring closure to give the major enantiomer (*R,R*) of the *trans* epoxide (Scheme 3). The main factors governing enantioselectivity are (i) conformational control of the ylide, (ii) facial selectivity of the ylide, and (iii) reversibility in formation of the betaine.^19^ Because factors i and ii were likely to be well controlled as the 1-naphthyl group is similar to an *o*-substituted aryl group (which is known to give a high er),^19^ we believed that factor iii, high reversibility during the formation of the betaine, was responsible for eroding the enantioselectivity. We, therefore, sought conditions that would reduce betaine reversibility. These include using more protic media to enhance the solvation of the betaine or using less ionic metals (Li instead of K), each of which would reduce the barrier to bond rotation (Scheme 3). Another method for limiting reversibility is to reduce temperature; at higher temperatures, the barrier to fragmentation of the betaine back to starting materials will be lower due to entropy. We first investigated different protic conditions (Table 2, entries 2–5), but in this case, we saw little improvement in er. However, using LiHMDS as a base at a low temperature (−78 °C) led to a significant increase in enantioselectivity to 85:15 er, albeit with a small decrease in diastereoselectivity (90:10). Further improvements in er to 96:4 were observed by decreasing the concentration and adding the base slowly with a syringe pump (entries 7 and 8).

**Scheme 3:**
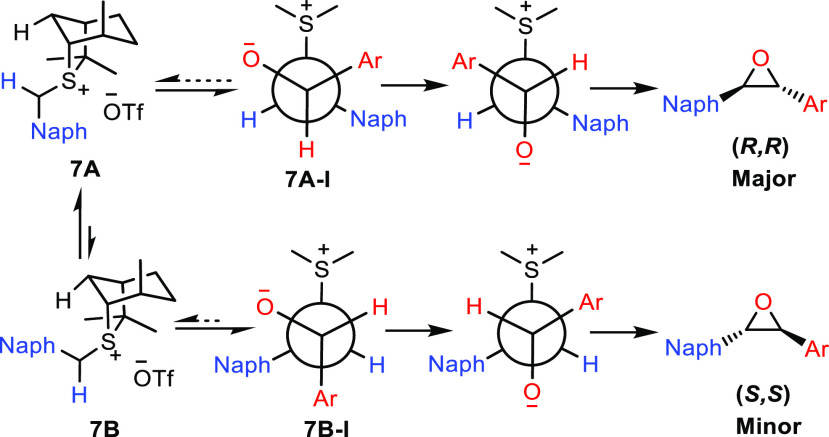
Rationale for Enantioselectivity in Epoxidation

The unexpected challenges in obtaining high selectivity warrant further discussion. The model for enantioselectivity is shown in Scheme 3.^20,21^ Minor ylide conformer **7B** reacts on the front face to give betaine **7B-I**, which undergoes bond rotation and ring closure to give the minor enantiomer (*S,S*) of the *trans* epoxide. The lower-than-expected enantioselectivity must originate from a greater degree of reversibility of betaine formation, presumably caused by the greater steric hindrance of the 1-naphthyl substituent as well as the *o*-substituted quinoline aldehyde. The steric hindrance in both components will result in a higher barrier to the bond rotation step and a lower barrier to fragmentation back to the starting materials, thereby leading to greater reversibility and lower enantioselectivity. However, conformers **7A** and **7B** do not react reversibly to the same extent. The minor conformer of ylide **7B**, being less stable than **7A**, will react less reversibly than **7A** and so will lead to an increased amount of the unwanted enantiomer (Curtin–Hammett).^22^ By using Li instead of K as the counterion on the alkoxide, the barrier to separating charges will be reduced, facilitating the bond rotation step, and by using lower temperatures, the level of entropy-driven fragmentation of the betaine back to starting materials will also be reduced. Both factors will reduce reversibility in betaine formation and consequently lead to higher enantioselectivity (Scheme 3).

Having achieved high enantioselectivity in epoxide formation, we focused our efforts on completing the synthesis. Regioselective ring opening of *trans* epoxide **(+)-3** with PhMgBr and CuCN gave alcohol **(+)-18** with higher regioselectivity (24:1), presumably as a consequence of the presence of the 6-bromo substituent, compared to 5:1, which was observed in its absence (Table 1). The subsequent steps followed Chandrasekhar’s protocol.^9^ Alcohol **(+)-18** was oxidized using Dess-Martin periodinane,^3^ giving ketone **(−)-10** in 90% yield, and subsequent addition of freshly prepared allylzinc bromide gave a 1:1 mixture of alcohols **19**.^9^ Generating the quaternary stereogenic center is a particularly challenging transformation, and despite considerable experimentation,^9^ this was the best selectivity achieved. Oxidative cleavage of the alkene using RuCl_3_ and NaIO_4_ gave the corresponding aldehyde, which was reduced *in situ* using NaBH_4_ to diol **20** in 88% yield over two steps.^24^ Tosylation of the primary alcohol followed by displacement of the tosyl group with dimethylamine gave a mixture of (+)-BDQ **(+)-1** and its epimer **21** in 90% yield over two steps.^24^ These diastereoisomers were separated by column chromatography to give the desired (+)-bedaquiline diastereoisomer **(+)-1** (Scheme 4). The overall yield was 8% from aldehyde **8**.

**Scheme 4:**
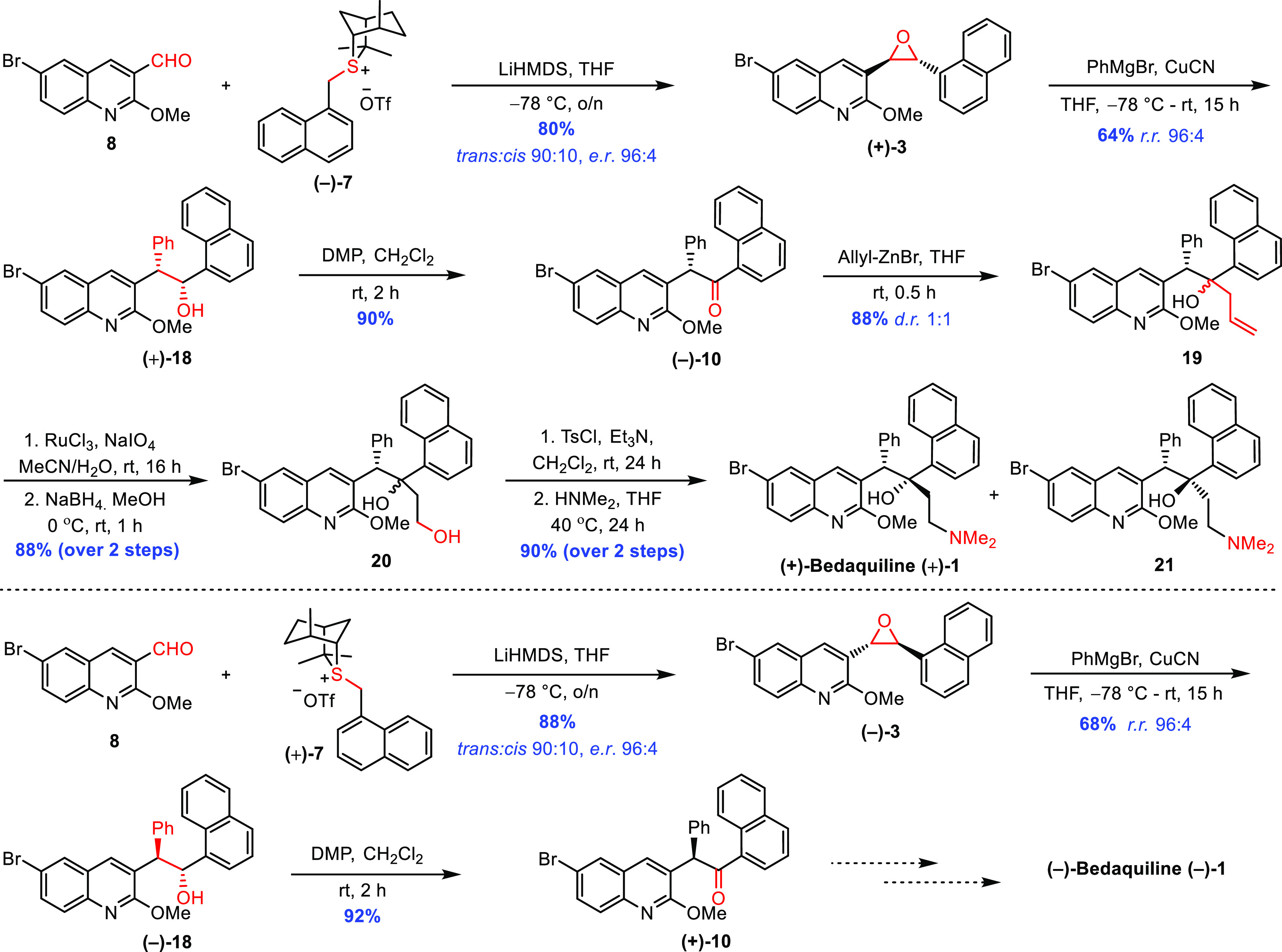
Total Synthesis of Bedaquiline (BDQ) via Sulfur Ylide Asymmetric Epoxidation Using Isothiocineole

The chemistry was repeated using (+)-isothiocineole **(+)-9** to give sulfonium salt **(+)-7**,^16,19^ which on treatment with aldehyde **8**(14,15) in the key step gave enantiomeric *trans*-epoxide **(−)-3** in 88% yield (dr 90:10, er 96:4). Ring opening of epoxide **(−)-3** with PhMgBr-CuCN gave the corresponding alcohol **(−)-18** with high regioselectivity (rr 96:4), and subsequent Dess-Martin periodinane oxidation^23^ afforded ketone **(+)-10** in 92% yield, thereby completing a formal total synthesis of (1*R*,2*S*)-(−)-bedaquiline **(−)-1** (Scheme 4).

In conclusion, we have successfully completed a nine-step synthesis of the potent anti-TB drug, bedaquiline. Key steps included an efficient sulfur ylide-mediated asymmetric epoxidation that, after optimization, afforded high enantio- and diastereoselectivity (er 96:4, dr 90:10 *trans*:*cis*). Furthermore, ring opening of the *trans* diaryl epoxide with PhMgBr-CuCN occurred with high regioselectivity (24:1) in favor of the desired regioisomer. Epoxides can also be formed by a Wittig reaction followed by asymmetric epoxidation, but the advantage of the sulfur ylide method is that it is not only much more atom economic as no wasteful phosphine oxides are generated but also a single-step reaction that controls both relative and absolute stereochemistry. The work highlights the successful application of a sulfur ylide-based methodology in the construction of complex molecules.

## Data Availability

The data underlying this study are available in the published article and its Supporting Information.

## Supporting Information Available

Experimental procedures, characterization data, ^1^H and ^13^C NMR spectra, chiral GC spectra, etc. (PDF)
