# Supplementary material for: Application of Enantioselective Sulfur Ylide Epoxidation to a Short Asymmetric Synthesis of Bedaquiline, a Potent Anti-Tuberculosis Drug
Source: Org Lett. 2023 Jun 7;25(23):4281–5. doi: 10.1021/acs.orglett.3c01286 (PMC10278180; doi:10.1021/acs.orglett.3c01286)
Supplement: Supplementary file 1 — ol3c01286_si_001.pdf [file ol3c01286_si_001.pdf]

SUPPORTING INFORMATION

Application of Enantioselective Sulfur Ylide Epoxidation to a Short Asymmetric Synthesis of Bedaquiline, a Potent Anti-TB Drug

Maryam Bashir,[a,b] Muhammad Arshad,*[c] Robina Begum,[b] Varinder K. Aggarwal*[a]

[a] School of Chemistry, University of Bristol, Cantock's Close, Bristol BS8 1TS, United Kingdom

[b] Centre for Organic Chemistry, School of Chemistry, University of the Punjab, Lahore-54590, Pakistan

[c] Institute of Chemistry, The Islamia University of Bahawalpur, Bahawalpur-63100, Pakistan

*e-mail: v.aggarwal@bristol.ac.uk

muhammad.arshad@iub.edu.pk

TABLE OF CONTENTS

1. MATERIALS AND GENERAL METHODS S3

1.1. Glassware, Solvents and Reagents S3

1.2. Chromatography and Instrumentation S3

1.3. Naming of Compounds S3

2. EXPERIMENTAL DATA S4

2.1. Synthesis substituted quinolines S4

2.1.1. 2-Chloroquinoline-3-carbaldehyde (24)[1] S4

2.1.2. 6-Bromo-2-chloroquinoline-3-carbaldehyde (17) [1] S4

2.1.3. 2-Methoxyquinoline-3-carbaldehyde (11) [2] S5

2.1.4. 6-Bromo-2-methoxyquinoline-3-carbaldehyde (8) [2] S5

2.2. Synthesis of sulfonium salt from alcohol S5

2.2.1. 1-(Naphthalen-1-ylmethyl)tetrahydro-1H-thiophen-1-ium tetrafluoroborate (12)[3,4] S5

2.3. Synthesis of isothiocineole S6

2.3.1. (1*R*,4*R*,5*R*)-4,7,7-Trimethyl-6-thiabicyclo[3.2.1]octane (–)-9 [3,4] S6

2.3.2.(1*S*,4*S*,5*S*)- 4,7,7-Trimethyl-6-thiabicyclo[3.2.1]octane (+)-9[3,4] S6

2.4. Synthesis of sulfonium salt from 1-(bromomethyl)naphthalene S6

2.4.1.(1*R*,4*R*,5*R*,6*R*)-4,7,7-Trimethyl-6-(naphthalen-1-ylmethyl)-6-thiabicyclo[3.2.1]octan-6-ium trifluoromethanesulfonate (−)-7[3,4] S6

2.4.2.(1*S*,4*S*,5*S*,6*S*)-4,7,7-Trimethyl-6-(naphthalen-1-ylmethyl)-6-thiabicyclo[3.2.1]octan-6-ium trifluoromethanesulfonate (+)-7[3,4] S7

2.5. Synthesis of epoxide using sulfonium salts S7

2.5.1. 2-Methoxy-3-((2*S*,3*S*)-3-(naphthalen-1-yl)oxiran-2-yl)quinoline (13) S7

2.5.2. 6-Bromo-2-methoxy-3-((2*R*,3*R*)-3-(naphthalen-1-yl)oxiran-2-yl)quinoline (+)-3 .... S8

2.5.3. 6-Bromo-2-methoxy-3-((2*S*,3*S*)-3-(naphthalen-1-yl)oxiran-2-yl)quinoline (−)-3 .... S9

2.6. Regioselective ring opening of epoxide 9

2.6.2.(1*R*,2*S*)-2-(6-Bromo-2-methoxyquinolin-3-yl)-1-(naphthalen-1-yl)-2-phenylethan-1-ol (+)-18 S10

2.6.3.(1*R*,2*R*)-2-(6-bromo-2-methoxyquinolin-3-yl)-1-(naphthalen-1-yl)-2-phenylethan-1-ol (−)-18 S10

2.7. Synthesis of ketone S11

2.7.1. (*S*)-2-(6-Bromo-2-methoxyquinolin-3-yl)-1-(naphthalen-1-yl)-2-phenylethan-1-one (−)-10 .... S11

2.7.2. (*R*)-2-(6-bromo-2-methoxyquinolin-3-yl)-1-(naphthalen-1-yl)-2-phenylethan-1-one (+)-10[6] S11

2.8. Allylation of ketone using allylzinc bromide S12

2.8.1. (1*S*)-1-(6-Bromo-2-methoxyquinolin-3-yl)-2-(naphthalen-1-yl)-1-phenylpent-4-en-2-ol and... S12

(1*S*)-1-(6-Bromo-2-methoxyquinolin-3-yl)-2-(naphthalen-1-yl)-1-phenylpent-4-en-2-ol (19) S12

2.9. Oxidative cleavage of alkene using $NaIO_4$ and $RuCl_3$ S12

2.9.1.(4*S*)-4-(6-Bromo-2-methoxyquinolin-3-yl)-3-hydroxy-3-(naphthalen-1-yl)-4-phenylbutanal (25)S12

2.10. Reduction of aldehyde using $NaBH_4$ S13

2.10.1.(4*S*)-4-(6-Bromo-2-methoxyquinolin-3-yl)-3-(naphthalen-1-yl)-4-phenylbutane-1,3-diol (20) S13

2.11. Tosylation of Alcohol S13

2.11.1 (4*S*)-4-(6-Bromo-2-methoxyquinolin-3-yl)-3-hydroxy-3-(naphthalen-1-yl)-4-phenylbutyl-4-methylbenzenesulfonate (26) S13

2.12. Synthesis of (1*S*,2*R*) (+)-Bedaquiline (+)-1 and (1*S*,2*S*) epimer 21 .... S14

2.12.1.(1*S*,2*R*)-1-(6-Bromo-2-methoxyquinolin-3-yl)-4-(dimethylamino)-2-(naphthalen-1-yl)-phenylbutan 2-ol (+)-1 .... S14

2.12.2.(1*S*,2*S*)-1-(6-Bromo-2-methoxyquinolin-3-yl)-4-(dimethylamino)-2-(naphthalen-1-yl)-1-phenylbutan-2-ol (21) S14

3.1. Experimentation for synthesis of Trisubstituted epoxides S15

3.1.1. 2-methoxy-3-((2S,3S)-3-methyl-3-(pyridin-4-yl)oxiran-2-yl)quinoline (29) S15

3.1.2. 3-(dimethylamino)-1-(naphthalen-1-yl)propan-1-one (5)[8] S15

3.1.3. 2-(3-(2-methoxyquinolin-3-yl)-2-(naphthalen-1-yl)oxiran-2-yl)-N,N-dimethylethan-1-amine (31) S15

6. SPECTROSCOPIC DATA S17

^{1}H NMR (400 MHz, $CDCl_3$) of 24 (see procedure) S17

^{13}C NMR (100 MHz, $CDCl_3$) of 24 .... S17

^{1}H NMR (400 MHz, $CDCl_3$) of 17 (see procedure) S18

^{13}C NMR (100 MHz, $CDCl_3$) of 17 .... S18

^{1}H NMR (400 MHz, $CDCl_3$) of 11 (see procedure) S19

^{13}C NMR (100 MHz, $CDCl_3$) of 11 .... S19

^{1}H NMR (400 MHz, $CDCl_3$) of 8 (see procedure) S20

^{13}C NMR (100 MHz, $CDCl_3$) of 8 .... S20

^{1}H NMR (400 MHz, DMSO) of 12 (see procedure) S21

^{13}C NMR (100 MHz, DMSO) of 12 .... S21

^{1}H NMR (400 MHz, $CDCl_3$) of 9 (see procedure) S22

^{13}C NMR (100 MHz, $CDCl_3$) of 9 .... S22

^{1}H NMR (400 MHz, $CDCl_3$) of 7 (see procedure) S23

^{13}C NMR (100 MHz, $CDCl_3$) of 7 .... S23

^{1}H NMR (400 MHz, $CDCl_3$) of 13 (see procedure) S24

^{13}C NMR (100 MHz, $CDCl_3$) of 13 .... S24

^{1}H NMR (400 MHz, $CDCl_3$) of (+)-3 (see procedure) S25

^{13}C NMR (100 MHz, $CDCl_3$) of (+)-3 .... S25

^{1}H NMR (400 MHz, $CDCl_3$) of 14 (see procedure) S26

^{13}C NMR (100 MHz, $CDCl_3$) of 14 .... S26

^{1}H NMR (600 MHz, $CDCl_3$) of 15 (see procedure) S27

^{13}C NMR (150 MHz, $CDCl_3$) of 15 .... S27

^{1}H NMR (400 MHz, $CDCl_3$) of (+)-18 (see procedure) S28

^{13}C NMR (100 MHz, $CDCl_3$) of (+)-18 .... S28

^{1}H NMR (400 MHz, $CDCl_3$) of (−)-10 (see procedure) S29

^{13}C NMR (100 MHz, $CDCl_3$) of (−)-10 .... S29

^{1}H NMR (600 MHz, $CDCl_3$) of (+)-1 (see procedure) S30

^{13}C NMR (150 MHz, $CDCl_3$) of (+)-1 .... S30

^{1}H NMR (600 MHz, $CDCl_3$) of 21 (see procedure) S31

^{13}C NMR (150 MHz, $CDCl_3$) of 21 .... S31

Chiral Phase Chromatograms for Epoxides (+)-3 and (−)-3 .... S32

Chiral GC chromatograms S34

7. REFERENCES S36

1. MATERIALS AND GENERAL METHODS

1.1. Glassware, Solvents and Reagents

All manipulations were performed with oven-dried (130 °C for a minimum of 12 h) or flame-dried glassware using standard Schlenk techniques under an atmosphere of nitrogen, unless otherwise stated.

All anhydrous solvents were commercially supplied or dried using an Anhydrous Engineering Alumina Column Drying System (THF, Toluene, Et_2O, CH_2Cl_2). Reagents were purchased from commercial sources and used as received.

1.2. Chromatography and Instrumentation

Thin layer chromatography (TLC) was performed using Merck Kieselgel 60 F254 fluorescent treated silica, which was visualized under UV light, or by staining with aqueous basic potassium permanganate ($KMnO_4$) followed by heating, *p*-anisaldehyde solution followed by heating, Hanessian's stain (CAM stain) followed by heating, or an ethanolic solution of phosphomolybdic acid followed by heating, as stated.

Flash column chromatography (FCC) was carried out using Sigma-Aldrich silica gel (60 Å, 230–400 mesh, 40–63 μm) or a Biotage Isolera™ flash purification system. In cases where automated column chromatography was employed the solvent gradient and flow rate are indicated.

NMR spectra were recorded at various field strengths, as indicated, using Bruker 400 MHz, Varian VNMR 400 MHz, and 600 MHz for ^{1}H and ^{13}C acquisitions. All NMR spectra were recorded at 25 °C unless otherwise stated. Chemical shifts (δ) are reported in parts per million (ppm) and referenced $CDCl_3$ (^{1}H: 7.26 ppm; ^{13}C: 77.0 ppm) or DMSO-d_6 (^{1}H: 2.50 ppm; ^{13}C: 39.5 ppm). Coupling constants (*J*) are given in Hertz (Hz) and refer to apparent multiplicities (s = singlet, d = doublet, t = triplet, q = quartet, quin = quintet, sex = hextet, h = heptet, m = multiplet, br = broad signal, dd = doublet of doublets, etc.). The ^{1}H NMR spectra are reported as follows: chemical shift (multiplicity, coupling constants, number of protons). ^{13}C NMR values are reported up to one decimal after rounding off the second decimal digit.

High resolution mass spectra (HRMS) were recorded on a Bruker Daltonics MicrOTOF II by Electrospray Ionisation (ESI); a Thermo Scientific QExactive by Electron Ionisation (EI); a Thermo Scientific Orbitrap Elite by ESI or Atmospheric Pressure Chemical Ionisation (APCI); or a Bruker UltrafleXtreme by Matrix-assisted Laser Desorption/Ionisation (MALDI).

IR spectra were recorded neat as a thin film on a Perkin Elmer Spectrum One FT-IR. Selected absorption maxima (ν_{max}) are reported in wavenumbers (cm^{-1}).

1.3. Naming of Compounds

Compound names are those generated by ChemDraw Professional 20.0 software (PerkinElmer), following the IUPAC nomenclature.

2. EXPERIMENTAL DATA

2.1. Synthesis substituted quinolines

2.1.1. 2-Chloroquinoline-3-carbaldehyde (24)[1]

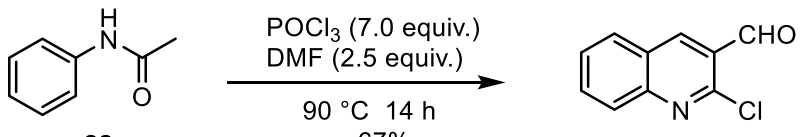

Compound **24** was prepared via literature procedure [1], with a slight modification in quenching reaction and purification. In a flame dried 500 mL 2 neck round bottom flask, *N,N*-dimethylformamide (7.12 mL, 92.3 mmol, 2.5 equiv.) was added and cooled to 0 °C. Phosphoryl chloride (24.2 mL, 259.0 mmol, 7.0 equiv.) was added dropwise keeping temperature between 0 °C to –5 °C under an inert atmosphere. To this solution, **22** (5.00 g, 37.0 mmol, 1.0 equiv.) was added and the reaction was stirred at room temperature for 5 mins then heated in an oil bath at 90 °C for 14 h. After completion (monitored by TLC), the reaction mixture was cooled to rt and added to 600 mL water under vigorous stirring, slowly keeping the temperature of mixture below 30 °C. After quenching reaction, it was placed in ice bath and stirred for 30 mins. Precipitates formed were filtered, washed with cold water, dried in vacuum, and recrystallized from ethyl acetate to give **24** (4.75 g, 67%) as yellow crystals.

TLC (SiO_2, Pent:EtOAc 7:3, $KMnO_4$, UV); R_f = 0.53; **^{1}H NMR** (400 MHz, $CDCl_3$) δ 10.58 (s, 1H), 8.78 (s, 1H), 8.09 (d, *J* = 8.5 Hz, 1H), 8.00 (d, *J* = 8.1 Hz, 1H), 7.90 (t, *J* = 8.5 Hz, 1H), 7.70-7.62 (m, 1H); **^{13}C NMR** (100 MHz, $CDCl_3$) δ 189.2, 140.3, 133.6, 129.7, 128.6, 128.2; **IR** (thin film) υ 2923, 2871, 1686, 1613, 1044, 760 cm^{-1}; **HRMS** (ESI) calcd for $C_{10}H_7ClNO$ $[M + H]^+$: 192.0211 m/z, found: 192.0214 m/z. See spectrum.

2.1.2. 6-Bromo-2-chloroquinoline-3-carbaldehyde (17) [1]

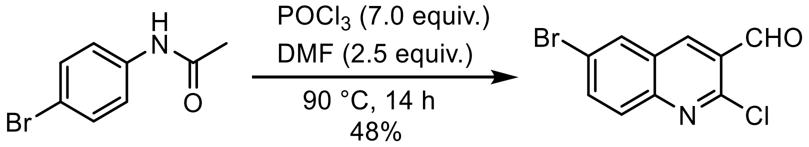

Compound **17** was prepared via a literature procedure [1], following **2.1.1** using **23** (5.00 g, 23.4 mmol, 1.0 equiv.). Crude product was recrystallized from ethyl acetate to give **17** (3.05 g, 48%) as orange solid.

TLC (SiO_2, Pent:EtOAc 7:3, $KMnO_4$, UV) R_f = 0.51; **^{1}H NMR** (400 MHz, $CDCl_3$) δ 10.56 (s, 1H), 8.67 (s, 1H), 8.15 (t, J = 1.4 Hz, 1H), 7.95 (d, *J* = 1.3 Hz, 2H); **^{13}C NMR** (100 MHz, $CDCl_3$) δ 188.8, 150.4, 148.1, 139.1, 137.0, 131.5, 130.2, 127.6, 127.0, 122.1; **IR** (thin film) υ 2978, 1685, 1574, 1483, 1043, 831 cm^{-1}; **HRMS** (EI) calcd for $C_{10}H_5BrClNO$ $[M]^+$: 268.9242 m/z, found: 268.9238 m/z. See spectrum.

2.1.3. 2-Methoxyquinoline-3-carbaldehyde (11) [2]

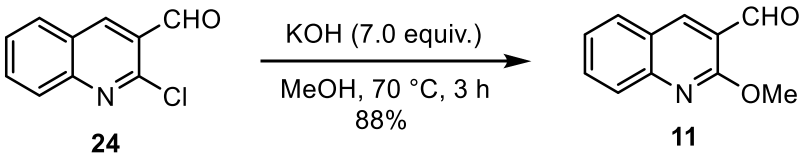

Compound **11** was prepared via literature procedure [2] with a slight modification in stoichiometry and temperature. A 500 mL round bottom flask was charged with a solution of **24** (3.00 g, 15.7 mmol, 1.0 equiv.) in MeOH (230 mL). KOH (6.16 g, 109.9 mmol, 7.0 equiv.) was added and the resulting reaction mixture was heated in an oil bath at 70 °C for 3 h. After complete consumption of starting material, reaction mixture was poured into 200 mL ice cold water. Precipitates formed were filtered, washed with cold water, dried in air, and purified by flash silica gel column chromatography (Pent:EtOAc 9:1) to afford **11** (2.58 g, 88%) as off-white solid.

TLC (SiO_2, Pent:EtOAc 9:1, $KMnO_4$, UV) R_f = 0.61; **^{1}H NMR** (400 MHz, $CDCl_3$) δ 10.46 (s, 1H), 8.57 (s, 1H), 7.85 (t, *J* = 8.3, Hz, 2H), 7.75 – 7.71 (m, 1H), 7.43 (m, 1H), 4.18 (s, 3H); **^{13}C NMR** (100 MHz, $CDCl_3$) δ189.3, 161.2, 149.0, 140.0, 132.5, 129.7, 127.2, 125.0, 124.4, 120.0, 53.8; **IR** (thin film) υ 2953, 1690, 1599, 1444, 1390, 1276 cm^{-1}; **HRMS** (ESI) calcd for $C_{11}H_{10}NO_2$ [M + H]$^+$: 188.0706 m/z, found: 188.0708 m/z. See spectrum.

2.1.4. 6-Bromo-2-methoxyquinoline-3-carbaldehyde (8) [2]

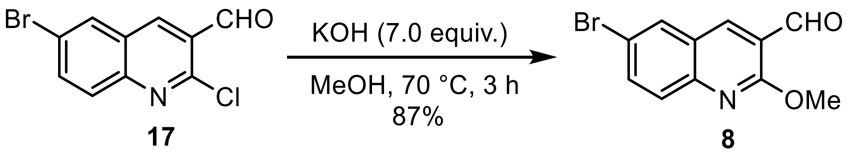

Compound **8** was prepared via literature procedure [2], following **2.1.3** using **17** (3.00 g, 11.1 mmol, 1.0 equiv.). Crude product was purified by flash silica gel column chromatography (Pent:EtOAc 9:1) to afford **8** (2.56 g, 87%) as light yellow solid.

TLC (SiO_2, Pent:EtOAc 9:1, $KMnO_4$, UV) R_f = 0.60; **^{1}H NMR** (400 MHz, $CDCl_3$) δ 10.46 (s, 1H), 8.49 (s, 1H), 7.99 (d, *J* = 2.1 Hz, 1H), 7.81 – 7.73 (m, 2H), 4.18 (s, 3H); **^{13}C NMR** (100 MHz, $CDCl_3$) δ 189.0, 161.4, 147.6, 138.7, 135.6, 131.4, 129.0, 125.5, 120.6, 118.2, 54.0; **IR** (thin film) υ 2950, 1688, 1596, 1488, 1341, 1267 cm^{-1}, **HRMS** (EI) calcd for $C_{11}H_8BrNO_2$ [M]$^+$: 264.9733 m/z, found: 264.9736 m/z. See spectrum.

2.2. Synthesis of sulfonium salt from alcohol

2.2.1. 1-(Naphthalen-1-ylmethyl)tetrahydro-1H-thiophen-1-ium tetrafluoroborate (12)[3,4]

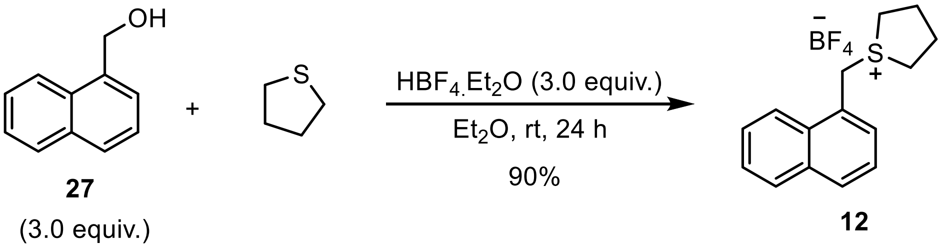

Using tetrahydrothiophene (0.50 mL, 5.67 mmol, 1.0 equiv.) compound **12** was prepared (1.79 g, 90%) according to literature procedure [3,4].

TLC (SiO_2, DCM:MeOH 9:1, $KMnO_4$, UV) R_f = 0.32; **^{1}H NMR** (400 MHz, DMSO) δ 8.44 (d, *J* = 8.6 Hz, 1H), 8.07 (t, *J* = 8.9 Hz, 2H), 7.79 (d, *J* = 7.0 Hz, 1H), 7.73 (ddd, *J* = 8.4, 6.8, 1.5 Hz, 1H), 7.66 (ddd, *J* = 7.9, 6.7, 1.1 Hz, 1H), 7.59 (dd, *J* = 8.2, 7.1 Hz, 1H), 5.00 (s, 2H), 3.51 – 3.36 (m, 4H), 2.47 – 2.35 (m, 2H), 2.26 – 2.12 (m, 2H); **^{13}C NMR** (100 MHz, DMSO) δ 133.6, 130.9, 130.7 (CH × 2), 129.1, 127.3, 126.8, 125.7, 125.4, 123.7, 43.2, 43.1 (CH_2 × 2), 28.2 (CH_2 × 2); **IR** (thin film) υ 2924, 1061, 1488, 777 cm^{-1}; **HRMS** (ESI) calcd for $C_{15}H_{17}S$ $[M]^+$: 229.1045 m/z, found: 229.1039 m/z. *See spectrum.*

2.3. Synthesis of isothiocineole

2.3.1. (1*R*,4*R*,5*R*)-4,7,7-Trimethyl-6-thiabicyclo[3.2.1]octane (–)-9 [3,4]

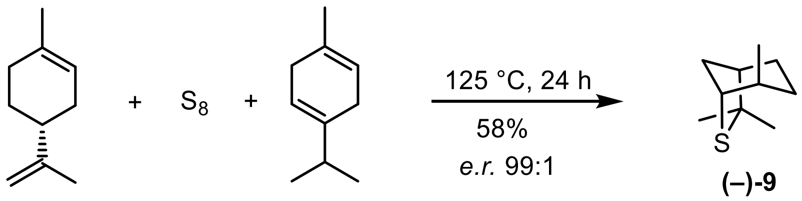

(–)-9

Starting from (+)-Limonene (6.81 g, 50.0 mmol, 1.0 equiv.) compound **(–)-9** was prepared (4.92 g, 58%) according to literature procedure [3].

$[\alpha]_D^{18}$ = –61.4 (*c* = 1.3 in $CHCl_3$); **^{1}H NMR** (400 MHz, $CDCl_3$) δ 3.30 (m, 1H), 2.31 (tt, *J* = 13.5, 6.2 Hz, 1H), 2.14 – 1.97 (m, 2H), 1.89 (m, 1H), 1.83 – 1.81 (m, 1H), 1.64 –1.59 (m, 2H), 1.49 (s, 3H), 1.37 (s, 3H), 1.14 (dd, *J* = 14.2, 5.5 Hz, 1H), 1.05 (d, *J* = 7.3 Hz, 3H); **^{13}C NMR** (100 MHz, $CDCl_3$) δ 52.6, 52.1, 47.1, 35.2, 34.7, 34.1, 25.2, 24.1, 23.6, 18.5; **IR** (thin film) υ 2953, 2920, 1461, 1346, 1375, 1145, 1048 cm^{-1}; **HRMS** (EI) calcd for $C_{10}H_{18}S$ $[M]^+$: 170.1124 m/z, found: 170.1124 m/z. *See spectrum.*

2.3.2.(1*S*,4*S*,5*S*)- 4,7,7-Trimethyl-6-thiabicyclo[3.2.1]octane (+)-9[3,4]

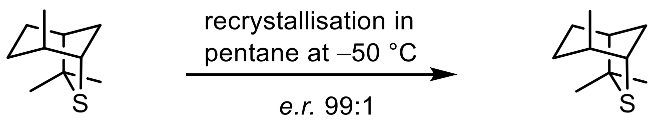

e.r. 90:10 **(+)-9**

Compound **(+)-9** was prepared via literature procedure [4] starting from (−)-limonene (6.81 g, 50.0 mmol, 1.0 equiv.), which gave (*S*)-sulfide **(+)-9** (5.14 g, 60%) in 90:10 *e.r.* Enantiopurity was enhanced by recrystallization in pentane at −50 °C to afford *e.r.* 99:1 in (2.60 g, 30% yield). **(+)-9** have the same NMR spectra as that of **(–)-9**.

$[\alpha]_D^{22}$ = +66.3 (*c* = 1.3 in $CHCl_3$); **HRMS** (EI) calcd for $C_{10}H_{18}S$ $[M]^+$: 170.1124 m/z, found: 170.1124 m/z.

2.4. Synthesis of sulfonium salt from 1-(bromomethyl)naphthalene

2.4.1.(1*R*,4*R*,5*R*,6*R*)-4,7,7-Trimethyl-6-(naphthalen-1-ylmethyl)-6-thiabicyclo[3.2.1]octan-6-ium

trifluoromethanesulfonate (−)-7[3,4]

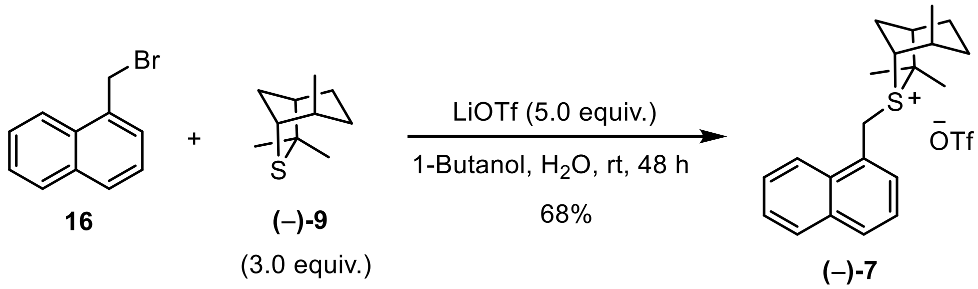

Starting from **16** (2.21 g, 10.0 mmol, 1.0 equiv.) compound **(−)-7** was prepared (3.16 g, 68%) according to literature procedure [3].

TLC (SiO_2, Pent:EtOAc 1:1, $KMnO_4$, UV) R_f = 0.62; **$[\alpha]_D^{23}$** = –176 (*c* = 1.5 in $CHCl_3$); **^{1}H NMR** (400 MHz, $CDCl_3$) δ 8.14 (d, *J* = 8.5 Hz, 1H), 7.73 (d, *J* = 8.3 Hz, 1H), 7.62 (q, J = 7.0 Hz, 3H), 7.47 (t, *J* = 7.6 Hz, 1H), 7.18 – 7.08 (m, 1H), 5.33 (d, *J* = 12.7 Hz, 1H), 4.91 (d, *J* = 12.7 Hz, 1H), 3.67 (s, 1H), 2.94 (m, 1H), 2.41 (s, 1H), 2.32 (d, *J* = 14.1 Hz, 1H), 1.95 (s, 3H), 1.88 (s, 3H), .83 – 1.66 (m, 3H), 1.66 – 1.38 (m, 2H), 0.96 (d, *J* = 7.0 Hz, 3H); **^{13}C NMR** (100 MHz, $CDCl_3$) δ 133.6, 131.74, 131.0, 130.72, 128.8, 127.8, 126.6, 125.4, 123.9, 123.3, 73.5, 64.2, 50.5, 39.9, 31.8, 31.7, 25.3, 25.2, 23.1, 22.1, 17.7; **IR** (thin film) υ 3004, 2945, 2865, 1455, 1256, 1172, 1028 cm^{-1}; **HRMS** (ESI) calcd for $C_{21}H_{27}S$ $[M]^+$: 311.1828 m/z, found: 311.1832 m/z. See spectrum.

2.4.2.(1*S*,4*S*,5*S*,6*S*)-4,7,7-Trimethyl-6-(naphthalen-1-ylmethyl)-6-thiabicyclo[3.2.1]octan-6-ium trifluoromethanesulfonate (+)-7[3,4]

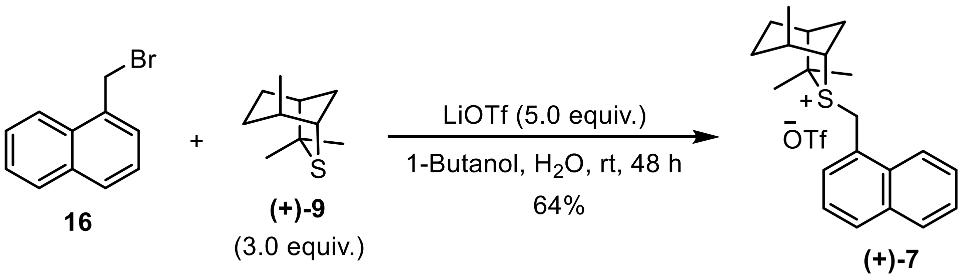

Starting from **16** (2.21 g, 10.0 mmol, 1.0 equiv.) compound **(+)-7** was prepared (2.97 g, 64%) according to literature procedure [3]. **(+)-7** have the same NMR spectra as that of **(–)-7**.

$[\alpha]_D^{22}$ = +200.1 (*c* = 1.5 in $CHCl_3$); **HRMS** (ESI) calcd for $C_{21}H_{27}S$ $[M]^+$: 311.1828 m/z, found: 311.1832 m/z.

2.5. Synthesis of epoxide using sulfonium salts

2.5.1. 2-Methoxy-3-((2*S*,3*S*)-3-(naphthalen-1-yl)oxiran-2-yl)quinoline (13)

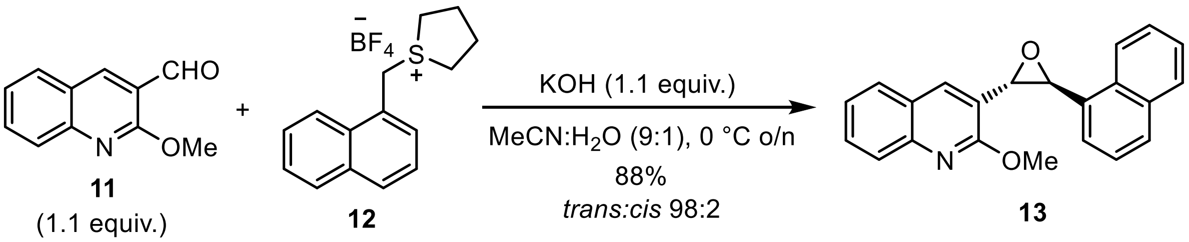

Starting from **12** (1.11 g, 3.50 mmol, 1.0 equiv.) compound **13** was obtained (1.01 g, 88%) following the literature procedure [3].

TLC (SiO_2, Pent:EtOAc 30:1, CAM, UV) R_f = 0.45 for ***trans*** and 0.43 for ***cis***; **^{1}H NMR** (400 MHz, $CDCl_3$) ***trans* isomer** δ 8.15 – 8.09 (m, 2H), 7.95 – 7.88 (m, 2H), 7.87 (d, *J* = 8.2 Hz, 1H), 7.80 (dd, *J* = 7.9, 1.5 Hz, 1H), 7.69 – 7.62 (m, 2H), 7.57 – 7.53 (m, 2H), 7.52 (dd, *J* = 2.3, 1.4 Hz, 1H), 7.43 (ddd, *J* = 8.1, 7.0, 1.2 Hz, 1H), 4.43 – 4.40 (m, 1H), 4.26 (dd, *J* = 2.1, 0.8 Hz, 1H), 4.11 (s, 3H); **^{13}C NMR** (100 MHz, $CDCl_3$) δ 160.5, 146.0, 133.7, 133.3, 133.0, 131.3, 129.6, 128.7, 128.4, 127.6, 127.0, 126.3, 126.0, 125.6, 125.1, 124.4, 123.2, 122.5, 122.3, 61.3, 57.5, 53.5; **IR** (thin film) υ 2977, 1638, 1400, 1262, 1062 cm^{-1}; **HRMS** (ESI) calcd for $C_{22}H_{18}NO_2$ $[M+H]^+$: 328.1332 m/z, found: 328.1334 m/z. *See spectrum.*

2.5.2. 6-Bromo-2-methoxy-3-((2*R*,3*R*)-3-(naphthalen-1-yl)oxiran-2-yl)quinoline (+)-3

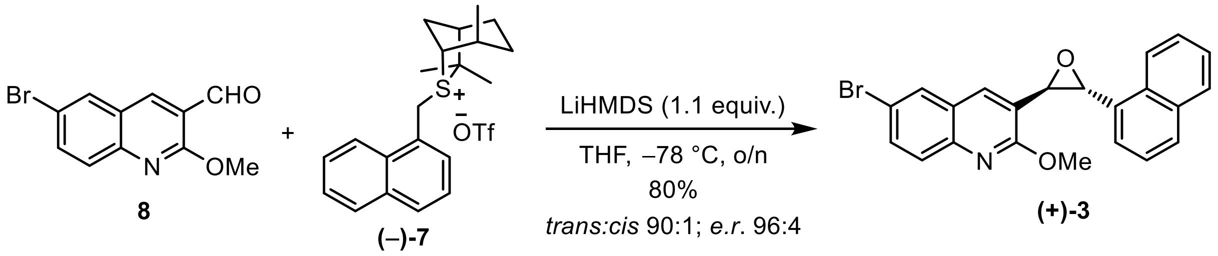

In a flame dried Schlenk tube, sulfonium salt **(−)-7** (1.15 g, 2.50 mmol, 1.0 equiv.) was dissolved in THF (62.5 mL, c= 0.04 M) and then aldehyde **8** (0.731 g, 2.75 mmol, 1.1 equiv.) was added. The reaction mixture was cooled to −78 °C and LiHMDS (1 M in THF) (2.75 mL, 2.75 mmol, 1.1 equiv.) was added using syringe pump (0.01 mL/min). After addition of base, reaction was stirred at same low temperature for overnight. Upon completion, the reaction was quenched with saturated aqueous NaCl solution and extracted with 25 mL DCM. The organic layer was separated, and aqueous layer was extracted twice with DCM (25 mL × 2). All organic layers were combined, dried over $MgSO_4$ and solvent removed under reduced pressure. Crude product was purified using flash silica gel column chromatography (Pent:EtOAc 100:0.5 to 100:3) to afford epoxide **(+)-3** as white solid (0.811 g, 80%).

TLC (SiO_2, Pent:EtOAc 20:1, CAM, UV) R_f = 0.72; **$[\alpha]_D^{22}$** = +24.0 (*c* = 1.5 in $CHCl_3$); **^{1}H NMR** (400 MHz, $CDCl_3$) δ 8.12 – 8.07 (m, 1H), 8.03 (s, 1H), 7.95 – 7.90 (m, 2H), 7.87 (d, *J* = 8.2 Hz, 1H), 7.77 (d, *J* = 8.9 Hz, 1H), 7.71 (dd, *J* = 8.9, 2.0 Hz, 1H), 7.66 (d, *J* = 7.0 Hz, 1H), 7.56 (dd, *J* = 2.4, 1.6 Hz, 1H), 7.54 – 7.51 (m, 2H), 4.39 (d, *J* = 2.0 Hz, 1H), 4.24 (dd, *J* = 2.0, 1.0 Hz, 1H), 4.10 (s, 3H); **^{13}C NMR** (100 MHz, $CDCl_3$) δ 160.6, 145.0, 133.3, 132.8, 132.7, 132.6, 131.3, 129.6, 128.7, 128.7, 128.5, 126.4, 126.3, 126.0, 125.5, 123.5, 123.1, 122.5, 117.6, 61.3, 57.3, 53.7; **IR** (thin film) υ 2948, 1625, 1599, 1466, 1401, 1050 cm^{-1}; **HRMS** (ESI) calcd for $C_{22}H_{17}BrNO_2$ $[M + H]^+$: 406.0443 m/z, found: 406.0431 m/z. *See spectrum.*

2.5.3. 6-Bromo-2-methoxy-3-((2*S*,3*S*)-3-(naphthalen-1-yl)oxiran-2-yl)quinoline (−)-3

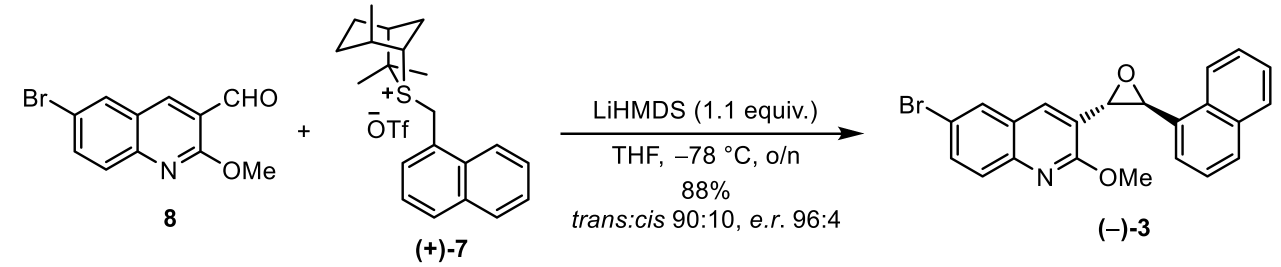

Following procedure given in **2.5.2**, using sulfonium salt **(+)-7** (576 mg, 1.25 mmol, 1.0 equiv.) enantiomeric epoxide **(–)-3** was prepared (447 mg, 88%). **(+)-3** has the same NMR spectra as that of **(–)-3**.

[α]$_D$^{21} = −26.66 (*c* = 1.5 in $CHCl_3$). **HRMS** (ESI) calcd for $C_{22}H_{17}BrNO_2$ $[M+H]^+$: 406.0443 m/z, found: 406.0431 m/z.

2.6. Regioselective ring opening of epoxide

2.6.1. (1*R*,2*R*)-2-(2-Methoxyquinolin-3-yl)-1-(naphthalen-1-yl)-2-phenylethan-1-ol (14) and (1*R*,2*S*)-1-(2-Methoxyquinolin-3-yl)-2-(naphthalen-1-yl)-2-phenylethan-1-ol (15)

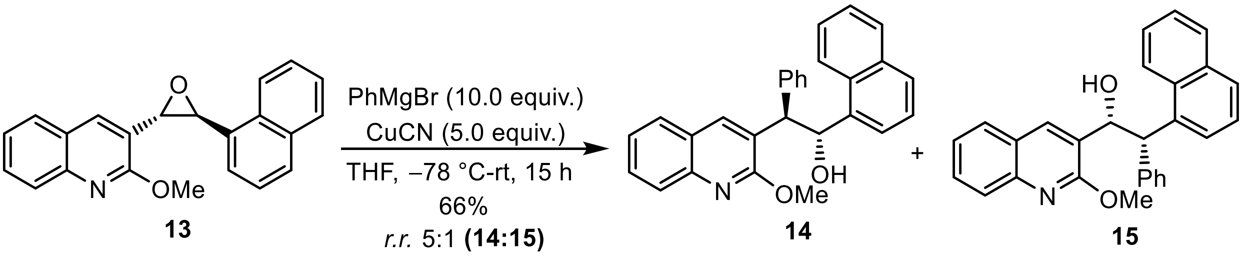

In a flame dried Schlenk tube, CuCN (233.6 mg, 3.05 mmol, 5.0 equiv.) was added followed by addition of THF (15 mL, *c* = 0.04 M) and cooled to −40 °C. To this suspension, PhMgBr (*c*= 0.87 M in THF, 7.02 mL, 6.11 mmol, 10.0 equiv.) was added and reaction mixture stirred at same low temperature for 1 h. The temperature of cryostat was then adjusted to −78 °C, and epoxide **13** (200 mg, 0.611 mmol, 1.0 equiv.) in 2 mL THF was added slowly using syringe pump (0.01 mL/min). After 2 hours, the reaction was allowed to warm to rt slowly by turning off cryostat (cooling bath was not removed). Over this period, the reaction mixture turned from white suspension to bright orange color. After completion, the reaction was quenched with 30 mL solution of NH_4OH:NH_4Cl (1:1). The reaction mixture turned blue, which was stirred for 2 h. Then it was extracted with EtOAc (15 mL × 3). Organic layers were combined, dried over $MgSO_4$ and concentrated under reduced pressure. The crude mixture was purified with flash silica gel column chromatography (Pent:EtOAc 9:1 to 1:1) to give an inseparable mixture of regioisomers **14** and **15**. This mixture of regioisomers was purified by recrystallization method. To this crude mixture, a minimum amount of DCM was added, white precipitates formed, which were separated. Again, DCM was added to the crude residue and precipitates formed were separated. The process was repeated until no further precipitates formed by adding DCM. Precipitates were dried to give desired regioisomer **14** (124 mg, 50%) and the rest was **15** (25 mg, 10%) after removal of DCM.

Regioisomer 14: TLC (SiO_2, Pent:EtOAc 7:3, CAM, UV) R_f = 0.34; **^{1}H NMR** (400 MHz, $CDCl_3$) δ 8.21 – 8.18 (m, 2H), 7.87 – 7.83 (m, 1H), 7.82 – 7.69 (m, 3H), 7.57, (t, *J* = 6.9 Hz, 1H), 7.53 – 7.42 (m, 2H), 7.38 (ddd, *J* =

8.1, 7.0, 1.2 Hz, 1H), 7.33 – 7.27 (m, 4H), 7.19 – 7.07 (m, 3H), 6.32 (dd, *J* = 6.7, 4.3 Hz, 1H), 5.21 (d, *J* = 6.7 Hz, 1H), 3.65 (s, 3H), 2.44 (d, *J* = 4.1 Hz, 1H); **^{13}C NMR** (100 MHz, $CDCl_3$) δ 160.8, 145.4, 141.1, 138.1, 138.9, 133.7, 130.8, 129.0, 128.8, 128.7 (CH × 2), 128.2 (CH × 2), 128.1, 127.4, 126.8, 126.5, 126.0, 125.3, 125.2, 125.1, 125.0, 124.2, 124.0, 123.2, 72.8, 53.3, 50.2; **IR** (thin film) υ 3430, 2949, 1633, 1572, 1400, 1261 cm^{-1}; **HRMS** (ESI) calcd for $C_{28}H_{24}NO_2$ [M + H]$^+$: 406.1802 m/z, found: 406.1797 m/z. See spectrum.

Regioisomer 15: TLC (SiO$_2$, Pent:EtOAc 7:3, CAM, UV) R_f = 0.35; **^{1}H NMR** (600 MHz, $CDCl_3$) δ 7.99 (dd, *J* = 7.9, 4.6 Hz, 2H), 7.87 (s, 1H), 7.81 (dd, *J* = 7.9, 1.7 Hz, 2H), 7.79 (d, *J* = 8.2 Hz, 1H), 7.63 – 7.59 (m, 2H), 7.59 – 7.56 (m, 1H), 7.41 – 7.37 (m, 1H), 7.36 (dt, *J* = 8.3, 1.9 Hz, 1H), 7.35 – 7.32 (m, 1H), 7.13 – 7.10 (m, 2H), 7.08 – 7.04 (m, 2H), 7.04 – 7.01 (m, 1H), 5.84 (dd, *J* = 8.8, 4.6 Hz, 1H), 5.37 (d, *J* = 8.5 Hz, 1H), 3.96 (s, 3H), 2.85 (d, *J* = 6.1 Hz, 1H); **^{13}C NMR** (150 MHz, $CDCl_3$) δ 160.0, 145.4, 141.1, 136.8, 136.3, 134.3, 132.6, 129.3, 128.9, 128.7 (CH × 2), 128.1 (CH × 2), 127.6, 127.5, 126.7, 126.5, 126.3, 126.1, 125.4, 125.3, 125.2, 125.0, 124.2, 123.5, 73.1, 53.6, 53.1; **IR** (thin film) υ 3398, 3057, 1624, 1505, 1396, 1240 cm^{-1}; **HRMS** (ESI) calcd for $C_{28}H_{24}NO_2$ [M + H]$^+$: 406.1801 m/z, found: 406.1803 m/z. see spectrum.

2.6.2. (1*R*,2*S*)-2-(6-Bromo-2-methoxyquinolin-3-yl)-1-(naphthalen-1-yl)-2-phenylethan-1-ol (+)-18

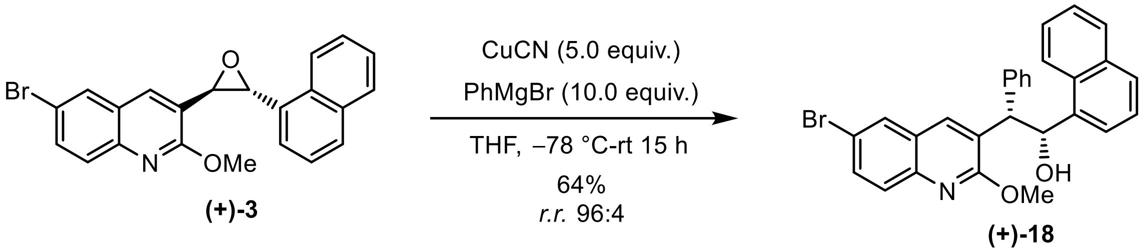

(+)-3 **(+)-18**

Applying procedure **2.6.1**, using **(+)-3** (600 mg, 1.47 mmol, 1.0 equiv.) afforded compound **(+)-18** (460 mg, 64%).

TLC (SiO$_2$, Pent:EtOAc 9:1, CAM, UV) R_f = 0.46; **[α]$_D^{22}$** = +14.0 (*c* = 1.0 in $CHCl_3$); **^{1}H NMR** (400 MHz, $CDCl_3$) δ 8.17 (d, *J* = 9.5 Hz, 1H), 8.12 (s, 1H), 7.90 (d, *J* = 2.0 Hz, 1H), 7.88 – 7.83 (m, 1H), 7.71 (d, 9.5 Hz, 1H), 7.69 – 7.60 (m, 2H), 7.54 – 7.46 (m, 2H), 7.31 – 7.24 (m, 4H), 7.20 – 7.08 (m, 3H), 6.28 (d, *J* = 6.4 Hz, 1H), 5.19 (d, *J* = 6.4 Hz, 1H), 3.60 (s, 3H), 2.39 (s, 1H); **^{13}C NMR** (100 MHz, $CDCl_3$) δ 161.0, 143.9, 140.9, 138.0, 137.0, 133.6, 132.2, 130.7, 129.5, 128.9, 128.7 (CH × 2), 128.5, 128.3 (CH × 2), 128.2, 126.6, 126.41, 126.37, 126.0, 125.4, 125.0, 124.1, 123.1, 117.1, 72.7, 53.5, 49.9; **IR** (thin film) υ 3411, 3028, 1617, 1597, 1462, 1257, 1003 cm^{-1}; **HRMS** (ESI) calcd for $C_{28}H_{23}BrNO_2$ [M +H]$^+$: 484.0912 m/z, found: 484.0909 m/z. See spectrum.

2.6.3. (1*R*,2*R*)-2-(6-bromo-2-methoxyquinolin-3-yl)-1-(naphthalen-1-yl)-2-phenylethan-1-ol (−)-18

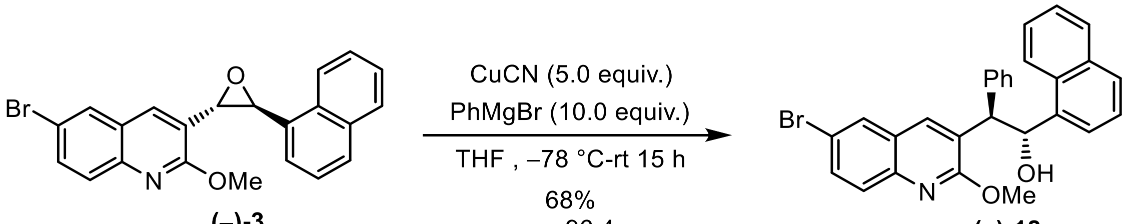

(–)-3 **(–)-18**

Applying procedure **2.6.1**, using epoxide **(−)-3** (536 mg,1.32 mmol, 1.0 equiv.) afforded compound **(−)-18** (440 mg, 68%). Compound **(−)-18** has same NMR data as that of **(+)18**.

[α]$_D^{22}$ = −4.0 (*c* = 1.0 in $CHCl_3$); **HRMS** (ESI) calcd for $C_{28}H_{23}BrNO_2$ [M + H]$^+$: 484.0912 m/z, found: 484.0909 m/z.

2.7. Synthesis of ketone

2.7.1. (*S*)-2-(6-Bromo-2-methoxyquinolin-3-yl)-1-(naphthalen-1-yl)-2-phenylethan-1-one (−)-10

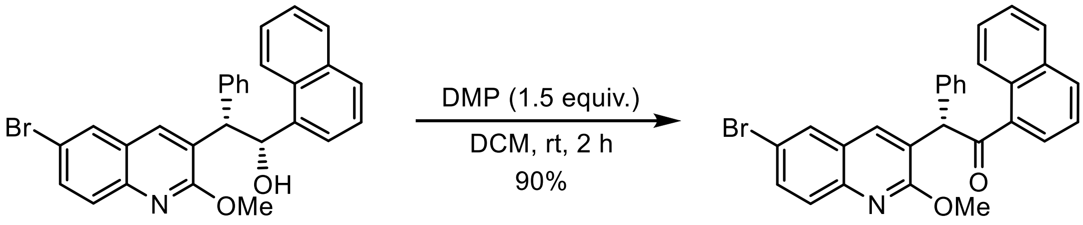

(+)-18 **(–)-10**

Following literature procedure [5], a solution of alcohol **(+)-18** (500 mg, 1.03 mmol, 1.0 equiv in DCM (15 mL) was prepared in a Schlenk tube. Dess-Martin periodinane (656 mg, 1.54 mmol, 1.5 equiv.) was added slowly. After stirring the mixture at rt for 2 h, reaction was quenched with saturated aqueous solution of $Na_2S_2O_3$ (15 mL) and saturated aqueous solution $NaHCO_3$ (15 mL) and stirred for 10 mins. The organic layer was separated, and aqueous layer was extracted twice with DCM (20 mL × 2). The combined organic layer was washed with brine, dried over $MgSO_4$ and solvent removed under reduced pressure. Crude mixture was purified by flash silica gel column chromatography (Pent:EtOAc 90:10) to afford ketone **(−)-10** as white solid (447 mg, 90%).

TLC (SiO_2, Pent:EtOAc 10:1, CAM, UV) R_f = 0.50; **[α]$_D^{20}$** = −176 (*c* = 1.5 in $CHCl_3$); **^{1}H NMR** (400 MHz, $CDCl_3$) δ 8.58 (d, *J* = 8.5 Hz, 1H), 8.07 (dd, *J* = 7.3, 1.2 Hz, 1H), 7.96 (d, *J* = 8.2 Hz, 1H), 7.86 (dd, *J* = 8.1, 1.5 Hz, 1H), 7.76 (d, *J* = 2.2 Hz, 1H), 7.73 (d, *J* = 8.9 Hz, 1H), 7.65 (dd, *J* = 8.9, 2.2 Hz, 1H), 7.58 (ddd, *J* = 8.6, 6.9, 1.6 Hz, 1H), 7.52 (ddd, *J* = 8.0, 6.8, 1.4 Hz, 1H), 7.50 – 7.41 (m, 6H), 7.40 – 7.34 (m, 1H), 6.26 (s, 1H), 3.99 (s, 3H); **^{13}C NMR** (100 MHz, $CDCl_3$) δ 200.9, 160.0, 144.4, 136.9, 136.0, 135.4, 133.9, 132.7, 132.3, 130.5, 129.7 (CH× 2), 129.6, 129.4 (CH × 2), 128.5, 128.3, 128.0, 127.9, 127.7, 126.9, 126.5, 126.4, 125.7, 124.3, 117.2, 57.3, 53.9; **IR** (thin film) υ 3020, 2910, 1686, 1597, 1463, 1254, 1062 cm^{-1}; **HRMS** (ESI) calcd for $C_{28}H_{21}BrNO_2$ [M + H]$^+$: 482.0753 m/z; found: 482.0756 m/z. *See spectrum*.

2.7.2. (*R*)-2-(6-bromo-2-methoxyquinolin-3-yl)-1-(naphthalen-1-yl)-2-phenylethan-1-one (+)-10[6]

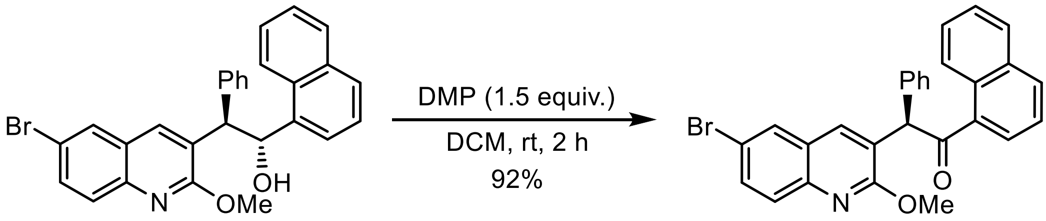

(–)-18 **(+)-10**

Applying procedure **2.7.1**, using alcohol **(−)-18** (500 mg,1.03 mmol, 1.0 equiv.) afforded compound **(+)-10** (460 mg, 92%). Optical rotation of compound **(+)-10** was compared with the literature [6], which confirmed the stereochemistry of the title compound. Compound **(+)10** has same NMR data as that of **(−)-10**.

[α]$_D^{20}$ = +153 (*c* = 1.5 in $CHCl_3$); **HRMS** (ESI) calcd for $C_{28}H_{21}BrNO_2$ [M + H]$^+$: 482.0754 m/z, found: 482.0753 m/z.

2.8. Allylation of ketone using allylzinc bromide

2.8.1. (1*S*)-1-(6-Bromo-2-methoxyquinolin-3-yl)-2-(naphthalen-1-yl)-1-phenylpent-4-en-2-ol and

(1*S*)-1-(6-Bromo-2-methoxyquinolin-3-yl)-2-(naphthalen-1-yl)-1-phenylpent-4-en-2-ol (19)

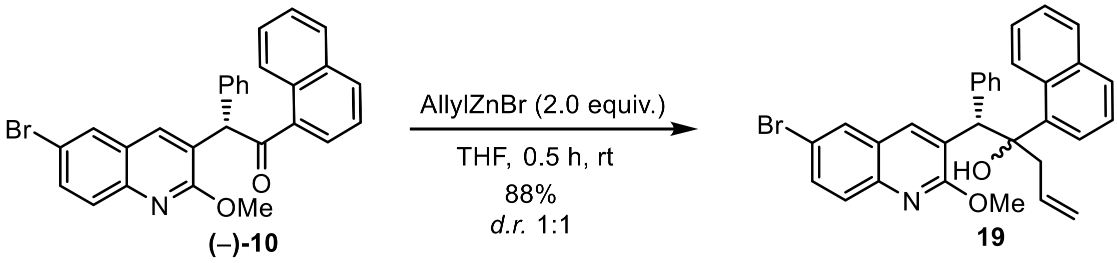

Allylation of ketone **(–)-10** was carried out by following a literature procedure [6]. A flame dried Schlenk tube was charged with freshly prepared solution of allylzinc bromide (*c* = 1 M in THF) (1.24 mL, 1.24 mmol, 2.0 equiv.). Ketone **(–)-10** (300 mg, 0.62 mmol, 1.0 equiv.) in THF (1.5 mL) was added to the above solution and stirred the mixture for 30 mins. The reaction mixture was quenched with saturated aqueous solution of NH_4Cl and extracted twice with EtOAc (10 mL × 2). The combined organic layer was washed with brine, dried over $MgSO_4$ and solvent removed under reduced pressure. The crude residue was purified by flash silica gel column chromatography (Pent:EtOAc 100:5) affording a mixture of inseparable diastereoisomers **19** as pale yellow solid (*d.r.* 1:1) (570 mg, 88%). This diastereomeric mixture proceeded to the next step.

TLC (SiO_2, Pent:EtOAc 10:1, CAM, UV) R_f = 0.60; **HRMS** (ESI) calcd for $C_{31}H_{27}BrNO_2$ $[M + H]^+$: 524.1227 m/z, found: 524.1225 m/z.

2.9. Oxidative cleavage of alkene using $NaIO_4$ and $RuCl_3$

2.9.1. (4*S*)-4-(6-Bromo-2-methoxyquinolin-3-yl)-3-hydroxy-3-(naphthalen-1-yl)-4-phenylbutanal (25)

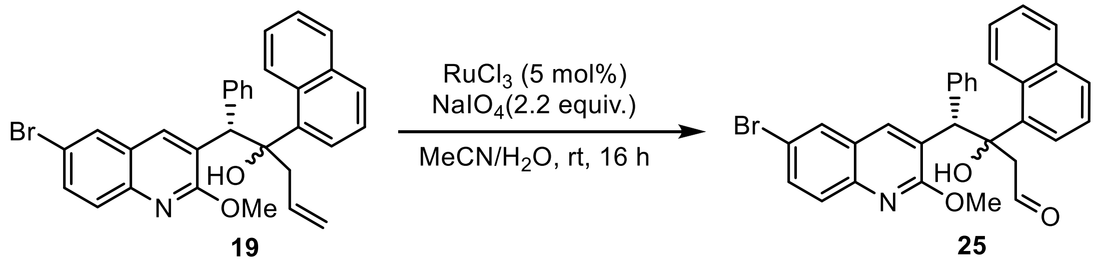

Aldehyde **25** was prepared using a literature procedure [7]. In a round bottom flask, a solution of alkene **19** (280 mg, 0.53 mmol,1.0 equiv.) and $RuCl_3$ (2.87 mg, 5 mol%) in acetonitrile (3 mL) was stirred for 10 mins. $NaIO_4$ (251 mg, 1.17 mmol, 2.2 equiv.) was added followed by portion wise addition of H_2O (120 µL × 10) in 30 mins. The green-colored reaction mixture was stirred at rt for 16 h. Upon completion, the reaction was quenched by $Na_2S_2O_3$ (15 mL, 10% w/v). Reaction mixture was extracted with EtOAc (10 mL × 3), combined organic layer was washed with brine, dried over $MgSO_4$ and solvent removed under reduced pressure to give aldehyde **25**, which was immediately used for next reaction without any manipulation.

TLC (SiO_2, Pent:EtOAc 10:1, CAM, UV) R_f = 0.31; **HRMS** (ESI) calcd for $C_{30}H_{25}BrNO_3$ $[M + H]^+$: 526.1010 m/z, found: 526.1018 m/z.

2.10. Reduction of aldehyde using $NaBH_4$

2.10.1. (4*S*)-4-(6-Bromo-2-methoxyquinolin-3-yl)-3-(naphthalen-1-yl)-4-phenylbutane-1,3-diol (20)

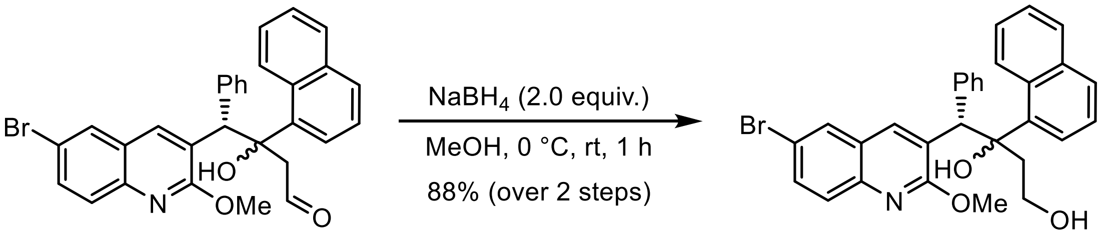

25 **20**

Compound **20** was prepared following a literature procedure [7]. A solution of aldehyde **25** (279 mg, 0.53 mmol, 1.0 equiv.) in MeOH (10 mL) was cooled to 0 °C, then $NaBH_4$ (40.1 mg, 1.06 mmol, 2.0 equiv.) was added. After this reaction mixture was stirred at rt for 1 h and then quenched with H_2O (15 mL). Volatile impurities were removed under reduced pressure and the crude mixture was extracted with EtOAc (10 mL × 3). Combined organic layer was washed with brine, dried over $MgSO_4$ and evaporated under reduced pressure. The crude mixture was purified by flash silica gel column chromatography (Pent:EtOAc 100:5 to 100:10) affording diol **20** as mixture of diastereoisomers (247 mg, 88% over 2 steps), which was subjected to next step.

TLC (SiO_2, Pent:EtOAc 10:2, CAM, UV) R_f = 0.34; **HRMS** (ESI) calcd for $C_{30}H_{27}BrNO_3$ $[M + H]^+$: 528.1169 m/z, found: 528.1169 m/z.

2.11. Tosylation of Alcohol

2.11.1 (4*S*)-4-(6-Bromo-2-methoxyquinolin-3-yl)-3-hydroxy-3-(naphthalen-1-yl)-4-phenylbutyl-4-methylbenzenesulfonate (26)

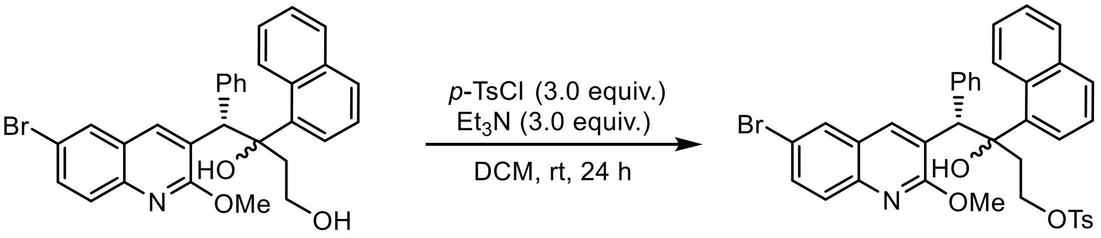

20 **26**

Following a literature procedure [7], a flame dried Schlenk tube was charged with a solution of diol **20** (120 mg, 0.23 mmol, 1.0 equiv.) in DCM (3 mL) and Et_3N (96 µL, 0.69 mmol, 3.0 equiv.) and the reaction mixture was cooled to 0 °C. *p*-TsCl (131.5 mg, 0.69 mmol, 3.0 equiv.) was added slowly and the resulting mixture stirred at rt for 24 h. After completion, the reaction was quenched with saturated aqueous solution of NH_4Cl (15 mL) and resulting mixture extracted with DCM (10 mL × 3). The combined organic layer was washed with brine, dried over $MgSO_4$ and solvent removed under reduced pressure. Crude residue was purified by flash silica gel column chromatography (Pent:EtOAc 80:20) affording tosylated mixture of two diastereoisomers **26**, which was subjected to next step.

TLC (SiO_2, Pent:EtOAc 8:2, CAM, UV) R_f = 0.38; **HRMS** (ESI) calcd for $C_{37}H_{33}BrNO_5S$ $[M + H]^+$: 682.1257 m/z, found: 682.1272 m/z.

2.12. Synthesis of (1*S*,2*R*) (+)-Bedaquiline (+)-1 and (1*S*,2*S*) epimer 21

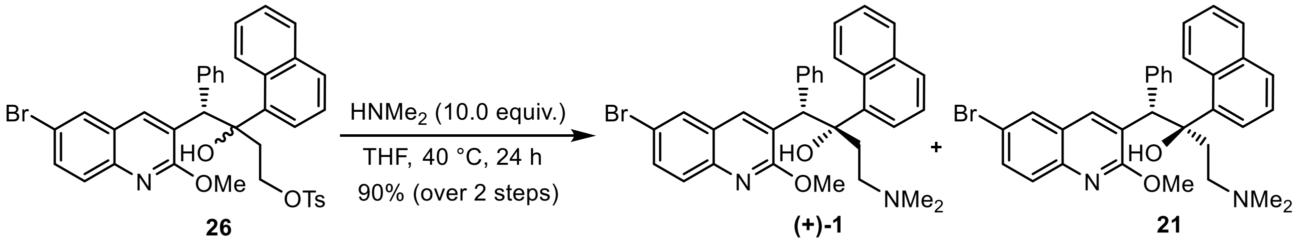

Following a literature procedure [6], conversion of **26** to target molecule (+)-bedaquiline **(+)-1** was carried out with a slight modification. In a flame dried Schlenk tube, tosylated alcohol **26** (142 mg, 0.21 mmol, 1.0 equiv.) and dimethyl amine (*c* = 2 M in THF, 2.10 mmol, 1.05 mL, 10.0 equiv.) were added and resulting mixture was heated using oil bath at 40 °C for 24 h. Upon completion, volatiles were removed under reduced pressure and crude mixture was purified by flash silica gel column chromatography (Pent:EtOAc 50:50) to afford **(+)-1** and its epimer **21** (105.2 mg, 90% over 2 steps).

2.12.1.(1*S*,2*R*)-1-(6-Bromo-2-methoxyquinolin-3-yl)-4-(dimethylamino)-2-(naphthalen-1-yl)-phenylbutan 2-ol (+)-1

TLC (SiO_2, Pent:EtOAc 1:1, $KMnO_4$, UV) R_f = 0.26; **$[\alpha]_D^{22}$** = +109.6 (*c* = 0.3 in DMF); **Lit: $[\alpha]_D^{25}$** = –165.2 (c = 0.8, DMF) [6].**^{1}H NMR** (600 MHz, $CDCl_3$) δ 8.90 (s, 1H), 8.61 (d, *J* = 8.7 Hz, 1H), 7.97 (d, *J* = 2.3 Hz, 1H), 7.91 (d, *J* = 7.3 Hz, 1H), 7.88 (d, *J* = 8.1 Hz, 1H), 7.72 (d, *J* = 8.8 Hz, 1H), 7.68 – 7.63 (m, 2H), 7.61 (td, *J* = 6.9, 3.3 Hz, 1H), 7.49 (t, *J* = 7.4 Hz, 1H), 7.31 (t, *J* = 7.7 Hz, 1H), 7.16 – 7.10 (m, 2H), 6.92 – 6.85 (m, 3H), 5.89 (s, 1H), 4.22 (s, 3H), 2.54 (d, *J* = 14.4 Hz, 1H), 2.15 – 2.07 (m, 1H), 2.06 – 2.01 (m, 1H), 1.99 (s, 6H), 1.97 – 1.91 (m, 1H); **^{13}C NMR** (150 MHz, $CDCl_3$) δ 161.4, 143.8, 141.7, 140.6, 138.8, 134.7, 131.9, 129.94, 129.91 (CH × 2), 129.83, 129.78, 128.5, 128.1, 127.9, 127.4, 127.1 (CH × 2), 126.9, 125.8, 125.3, 125.1, 125.0, 124.5, 117.0, 82.5, 56.3, 54.2, 49.5, 44.7, 33.5, 29.7; **HRMS** (ESI) calcd for $C_{32}H_{32}BrN_2O_2$ [M + H]$^+$: 555.1642 m/z; found: 555.1627 m/z. *see spectrum*.

2.12.2.(1*S*,2*S*)-1-(6-Bromo-2-methoxyquinolin-3-yl)-4-(dimethylamino)-2-(naphthalen-1-yl)-1-phenylbutan-2-ol (21)

TLC (SiO_2, Pent:EtOAc 1:1, $KMnO_4$, UV), R_f = 0.23; **$[\alpha]_D^{22}$** = −19.51 (*c* = 0.4 in DMF), **$[\alpha]_D^{22}$** = +41.2 (c = 0.31, DMF) [6]; **^{1}H NMR** (600 MHz, $CDCl_3$) δ, 8.59 (s, 1H), 8.49 (d, *J* = 8.8 Hz, 1H), 8.00 (dd, *J* = 7.5, 1.4 Hz, 1H), 7.88 (dt, *J* = 8.3, 1.8 Hz, 2H), 7.80 (dd, *J* = 7.7, 1.8 Hz, 2H), 7.58 (d, *J* = 8.0 Hz, 1H), 7.55 (ddd, *J* = 8.5, 6.8, 1.5 Hz, 1H), 7.45 (dd, *J* = 8.8, 2.3 Hz, 1H), 7.44 – 7.41 (m, 1H), 7.39 – 7.34 (m, 3H), 7.34 – 7.23 (m, 3H), 5.74 (s, 1H), 3.26 (s, 3H), 2.49 (dt, *J* = 14.8, 3.2 Hz, 1H), 2.26 (ddd, *J* = 14.7, 12.5, 3.1 Hz, 1H), 2.08 (td, *J* = 12.8, 2.5 Hz, 1H), 2.01 (s, 6H), 1.97 (ddd, *J* = 16.5, 8.2, 3.7 Hz, 1H); **^{13}C NMR** (150 MHz, $CDCl_3$) δ 160.5, 143.3, 141.7, 141.2, 137.8, 134.7, 131.34, 131.29 (CH × 2), 130.1, 129.6, 129.5, 128.1, 128.0, 127.9 (CH × 2), 127.4, 127.2, 126.6, 126.5, 125.4, 124.9, 124.8, 124.4, 116.3, 81.6, 56.2, 52.7, 51.0, 44.6, 34.1, 29.7; **HRMS** (ESI) calcd for $C_{32}H_{32}BrN_2O_2$ [M + H]$^+$: 555.1642 m/z, found: 555.1631 m/z. *see spectrum*.

3.1. Experimentation for synthesis of Trisubstituted epoxides

3.1.1. 2-Methoxy-3-((2S,3S)-3-methyl-3-(pyridin-4-yl)oxiran-2-yl)quinoline (29)

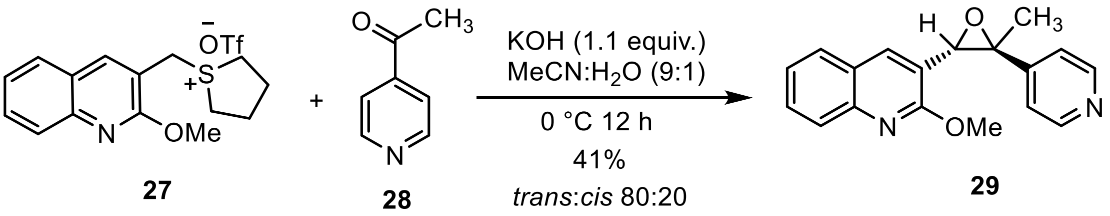

Following the literature procedure, starting from sulfonium salt **27** (205 mg, 0.50 mmol, 1.0 equiv.) and ketone **28** (66.6 mg, 0.55 mmol, 1.1 equiv.), trisubstituted epoxide **29** obtained (60 mg, 41%) [3]. This was test substrate to check whether the sulfonium salt and ketone would work under epoxidation conditions.

3.1.2. 3-(Dimethylamino)-1-(naphthalen-1-yl)propan-1-one (5)[8]

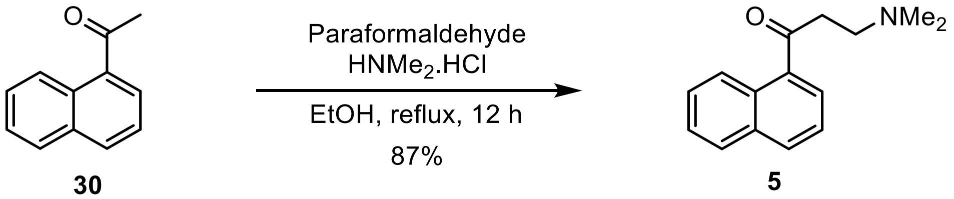

Following a literature procedure with slight modification, title compound was prepared. 1-Acetylnaphthelene **30** (851 mg, 5.0 mmol, 1.0 equiv.) afforded product **5** (995 mg, 87%) [8].

3.1.3. 2-(3-(2-Methoxyquinolin-3-yl)-2-(naphthalen-1-yl)oxiran-2-yl)-N,N-dimethylethan-1-amine (31)

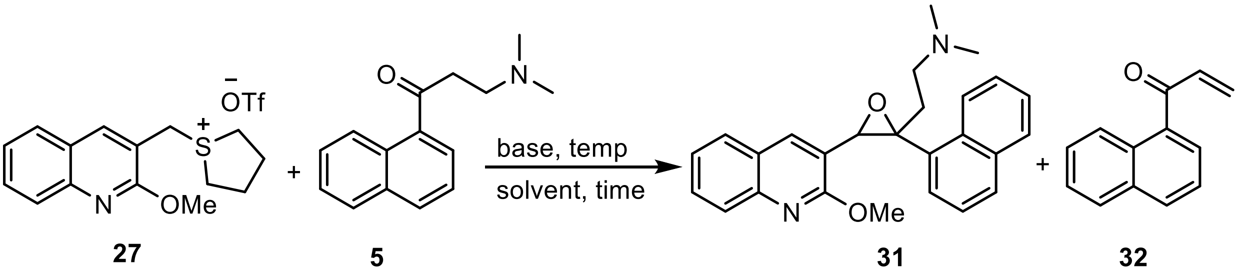

Following literature procedure, starting from sulfonium salt (41 mg, 0.10 mmol, 1.0 equiv.), the conditions below were tested for the synthesis of trisubstituted epoxide [9]. Unfortunately, none were successful, possibly because the ketone was too hindered. As such the research focused on the synthesis of disubstituted epoxides.

Entry	Base (equiv.)	Solvent (ratio)	Temp (°C)	Comment *a*
**1**	KOH (10.0)	MeCN/H_2O (9:1)	0	Product **31** 4%, RSM **5** 75%
**2**	KOH (1.1)	MeCN/H_2O (9:1)	0	No product, alkene **32** 30%
**3**	P_2–Et (1.0)	THF	−78	No product, messy NMR

**4**	KHMDS (1.1)	THF	−78	No product observed
**5**	KOH (10.0)	MeCN/H_2O (4:1)	0	Product 4%, messy NMR

[a] NMR yields were determined using internal standard dibromomethane.

6. SPECTROSCOPIC DATA

^{1}H NMR (400 MHz, $CDCl_3$) of **24** (see procedure)

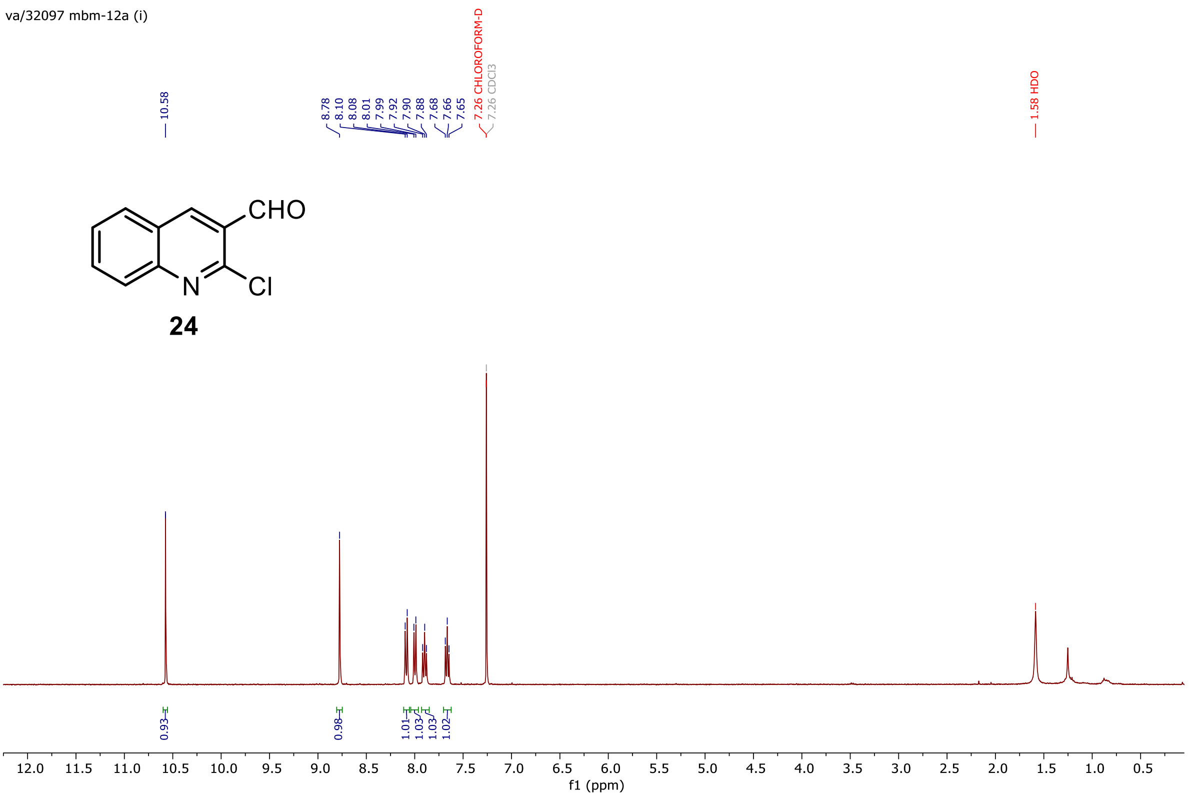

^{13}C NMR (100 MHz, $CDCl_3$) of **24**

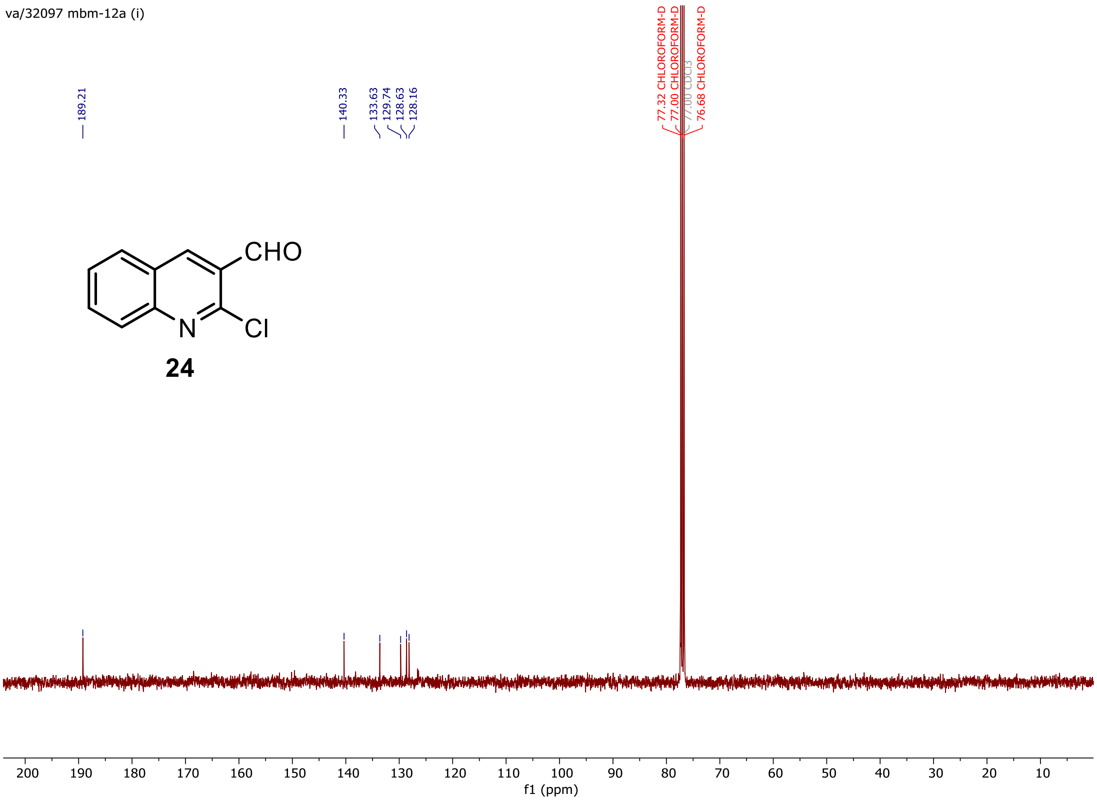

^{1}H NMR (400 MHz, $CDCl_3$) of **17** (see procedure)

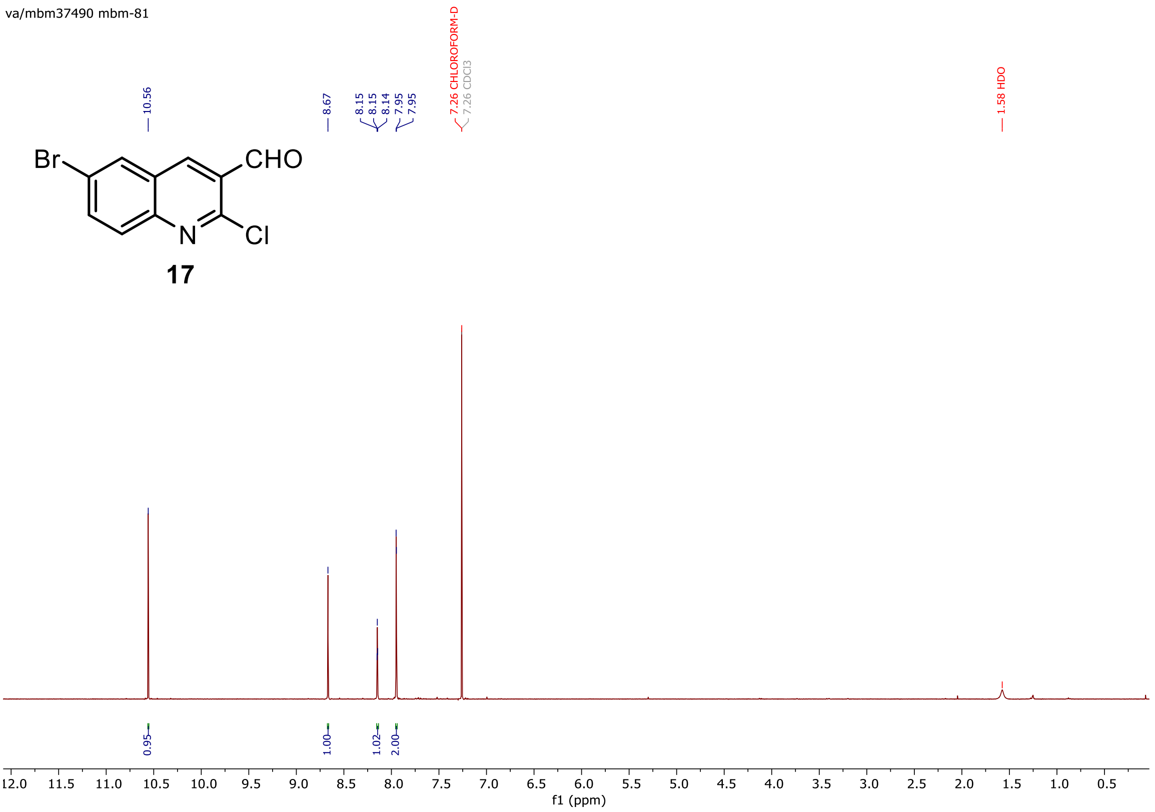

^{13}C NMR (100 MHz, $CDCl_3$) of **17**

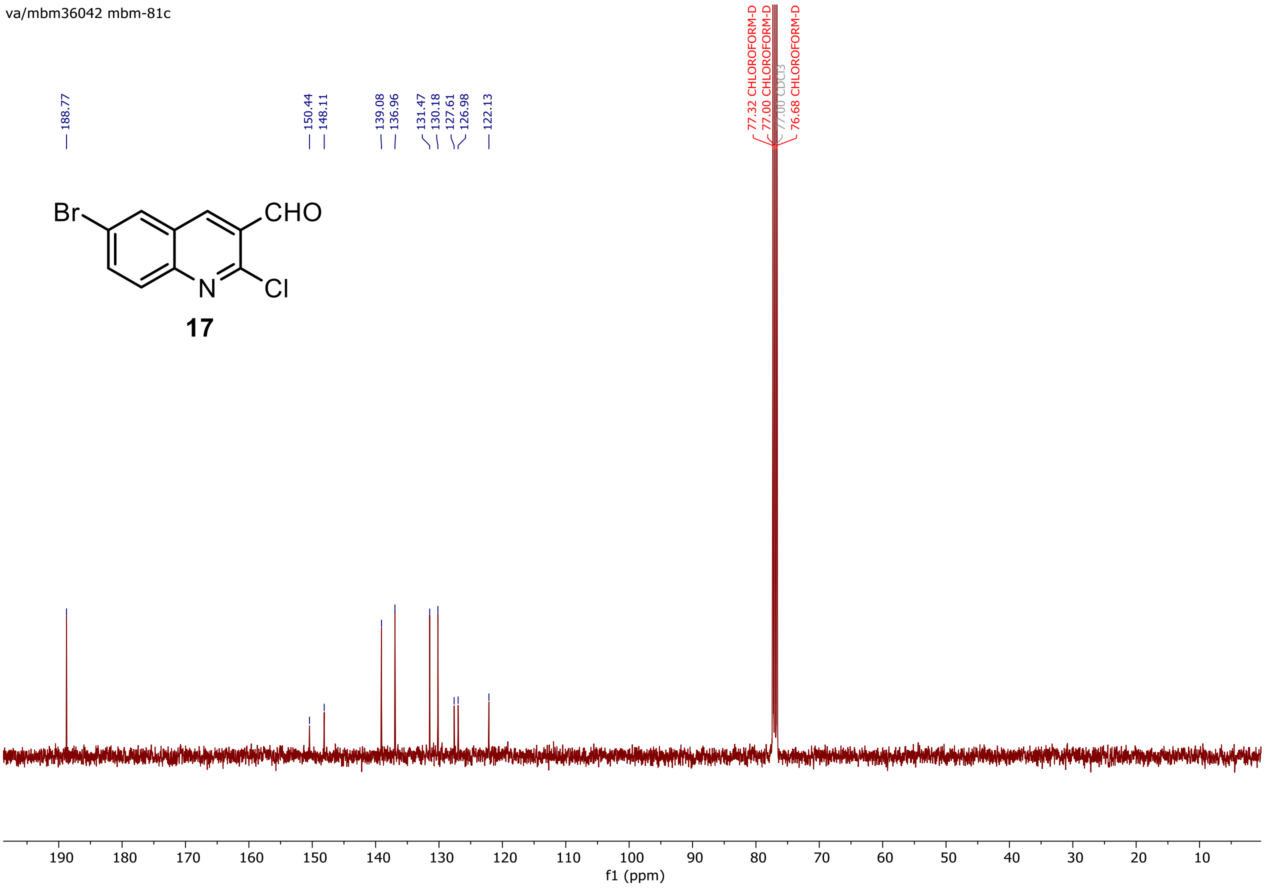

^{1}H NMR (400 MHz, $CDCl_3$) of **11** (see procedure)

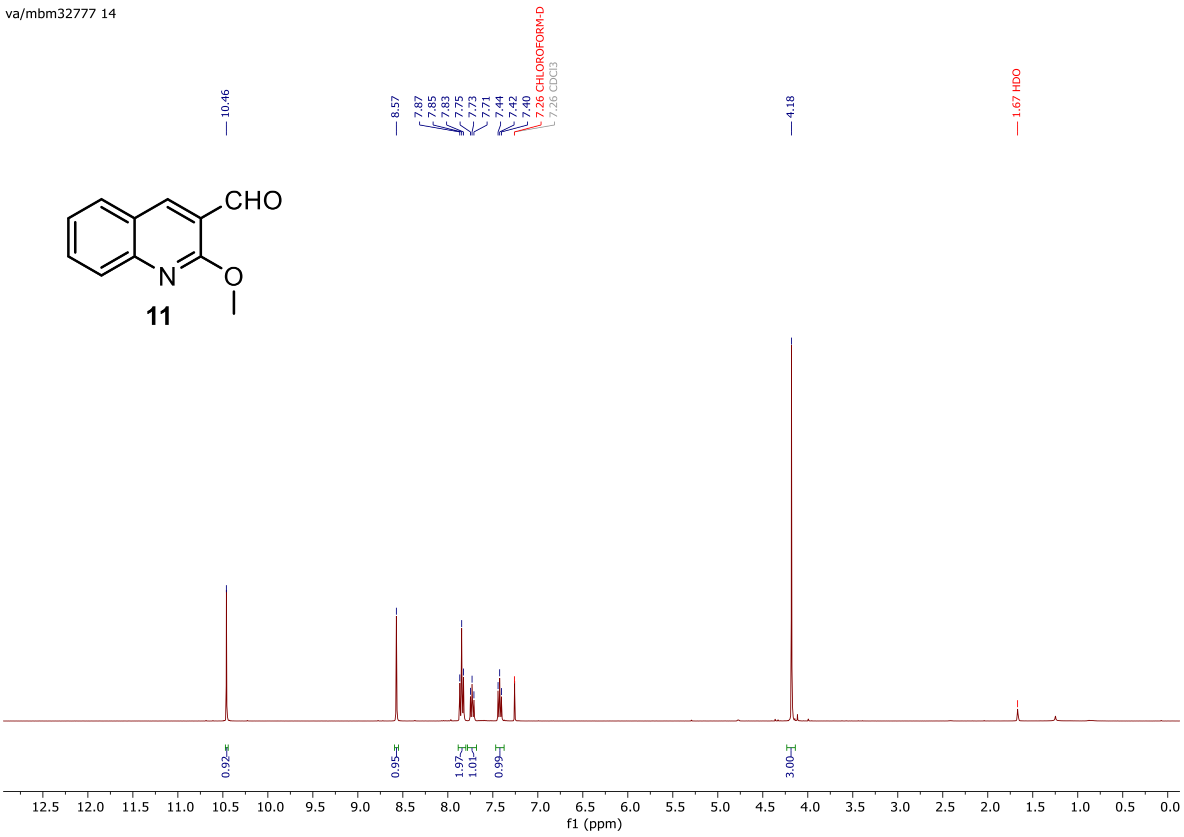

^{13}C NMR (100 MHz, $CDCl_3$) of **11**

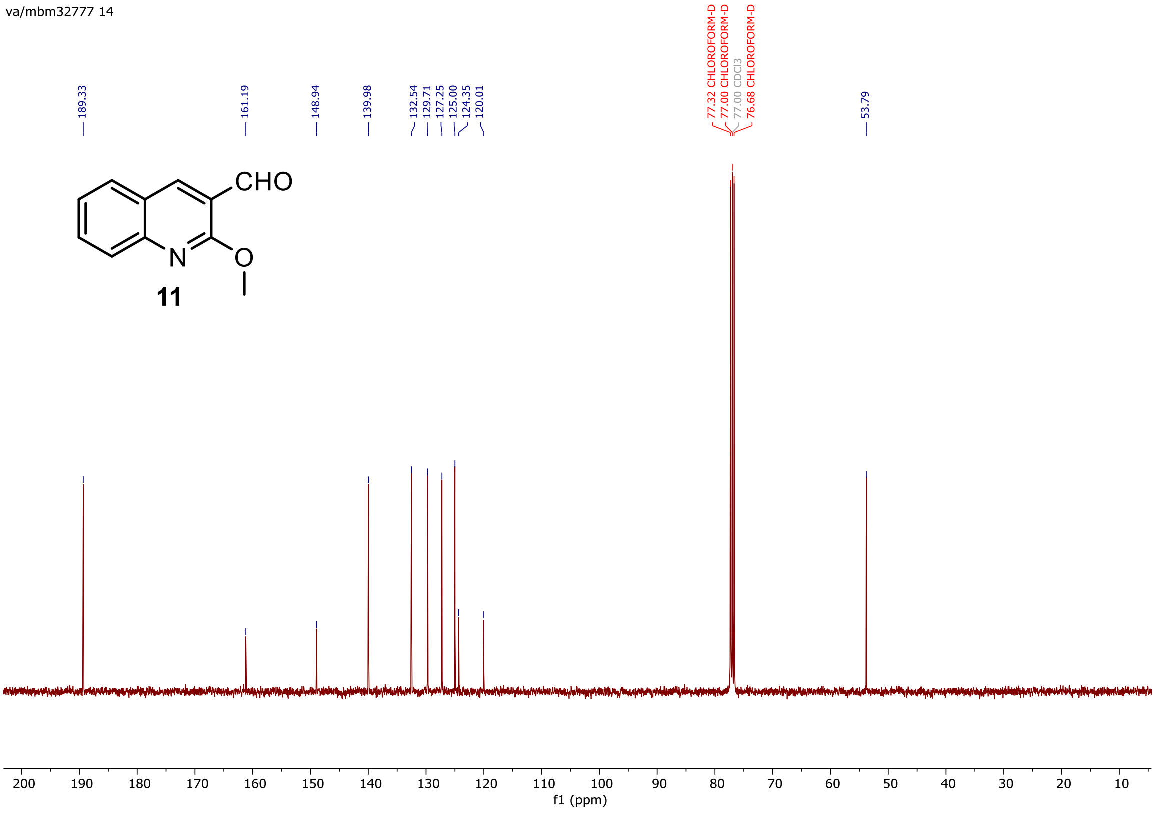

^{1}H NMR (400 MHz, $CDCl_3$) of **8** (see procedure)

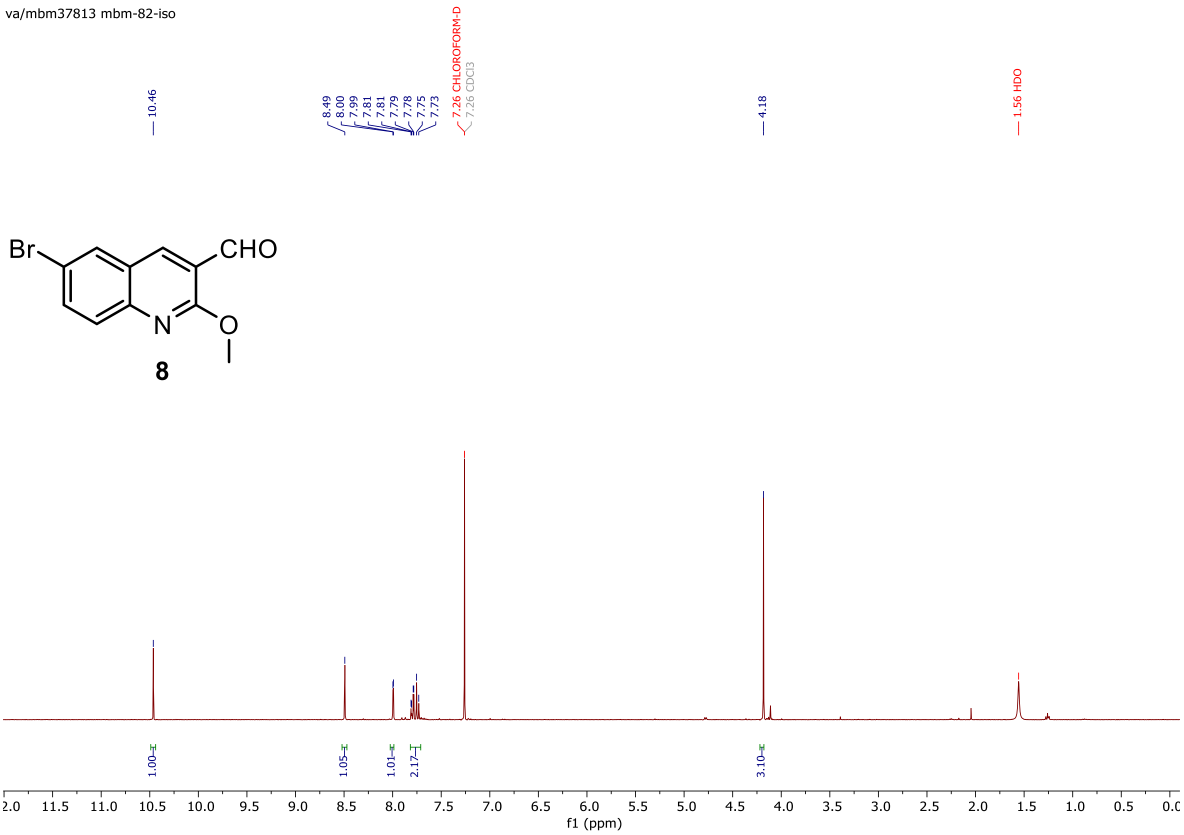

^{13}C NMR (100 MHz, $CDCl_3$) of **8**

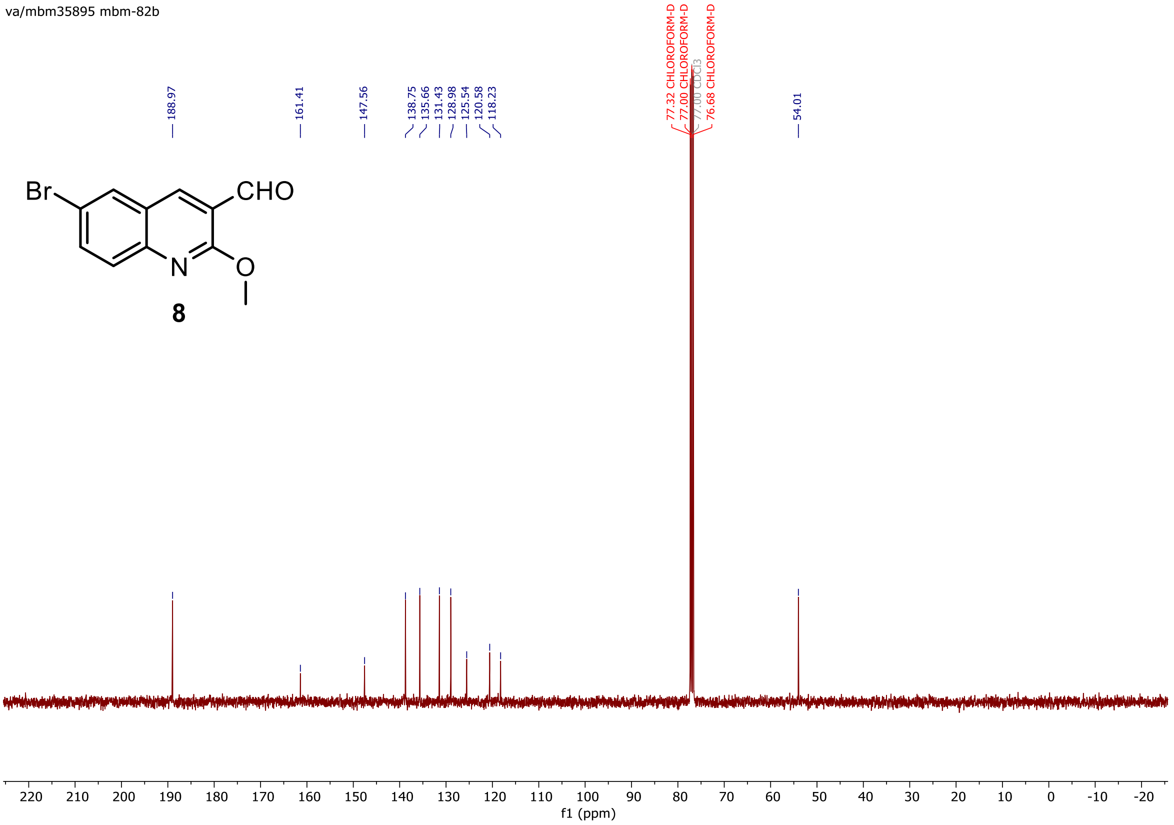

^{1}H NMR (400 MHz, DMSO) of **12** (see procedure)

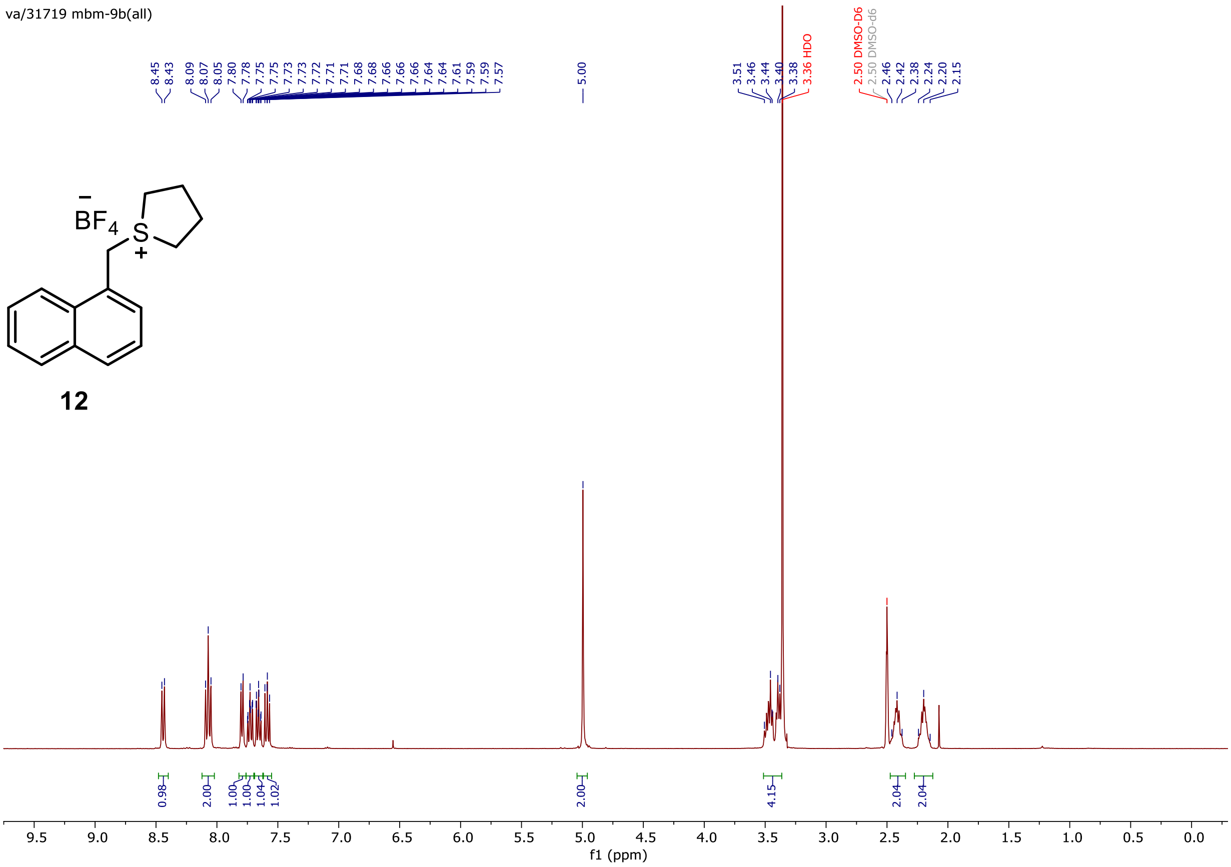

^{13}C NMR (100 MHz, DMSO) of **12**

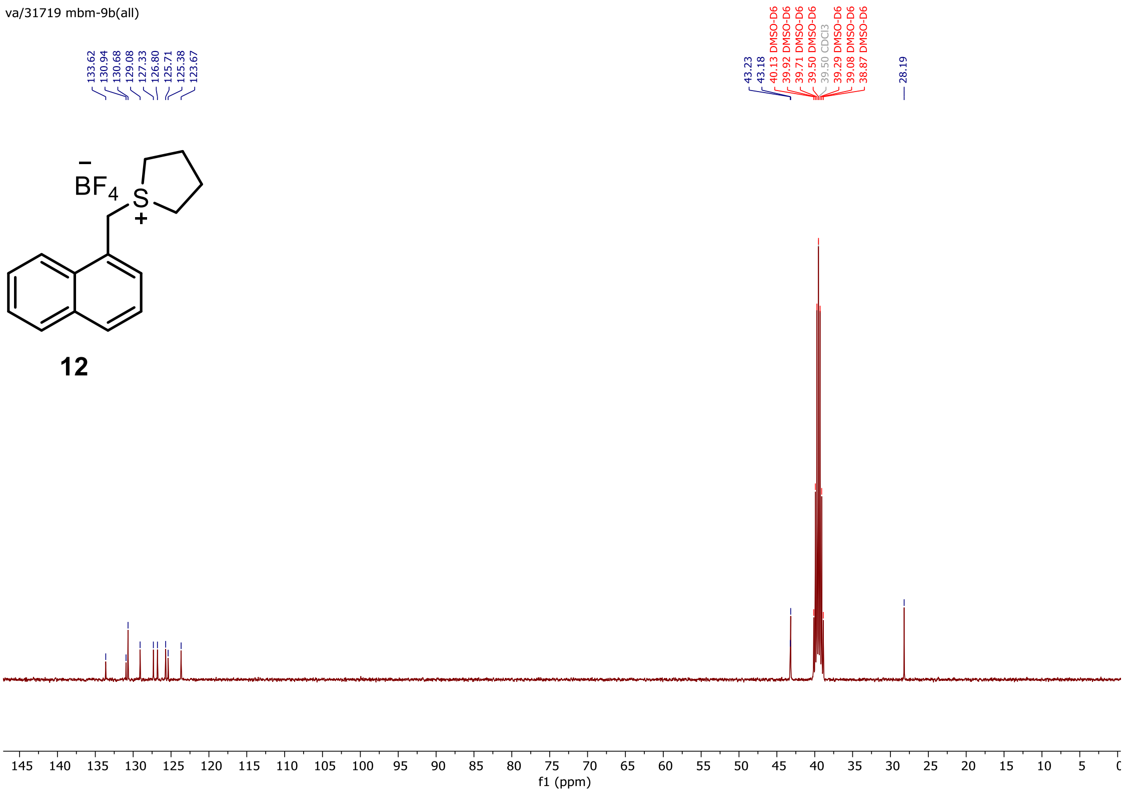

^1H NMR (400 MHz, $CDCl_3$) of **9** (see procedure)

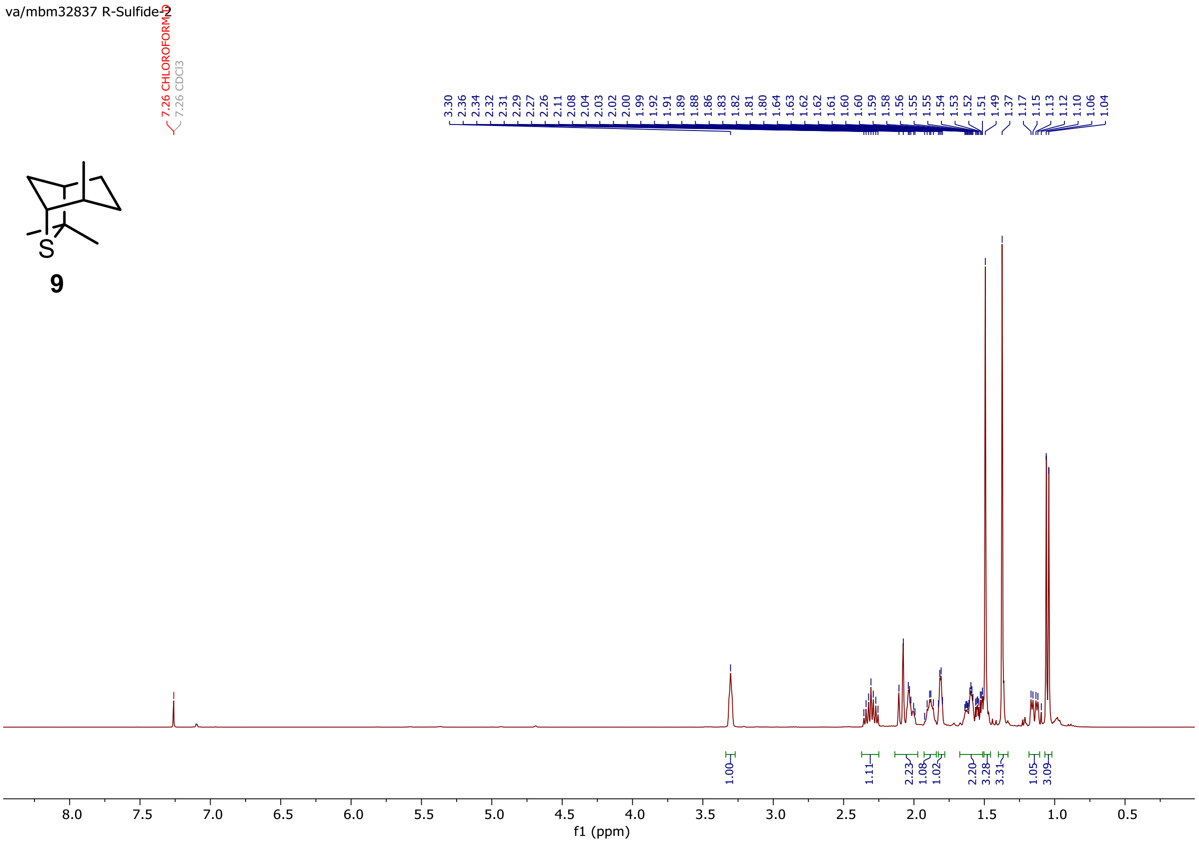

^{13}C NMR (100 MHz, $CDCl_3$) of **9**

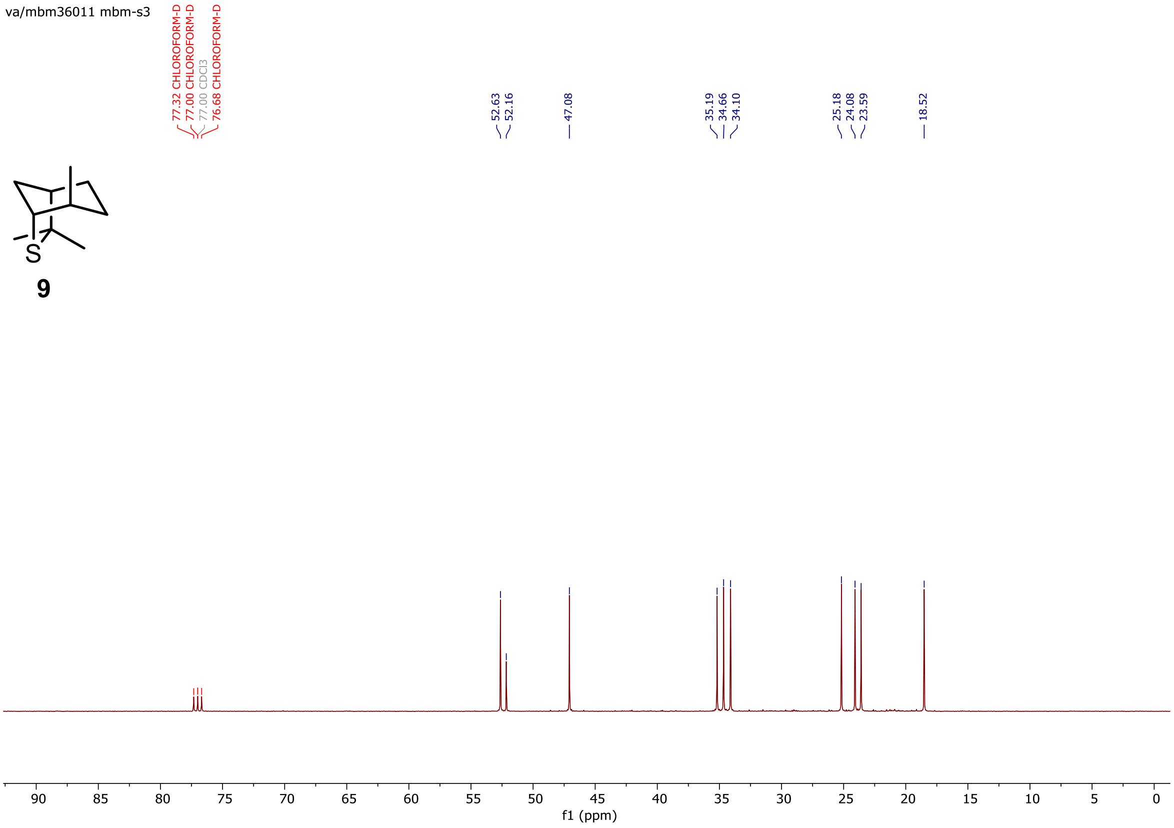

^{1}H NMR (400 MHz, $CDCl_3$) of **7** (see procedure)

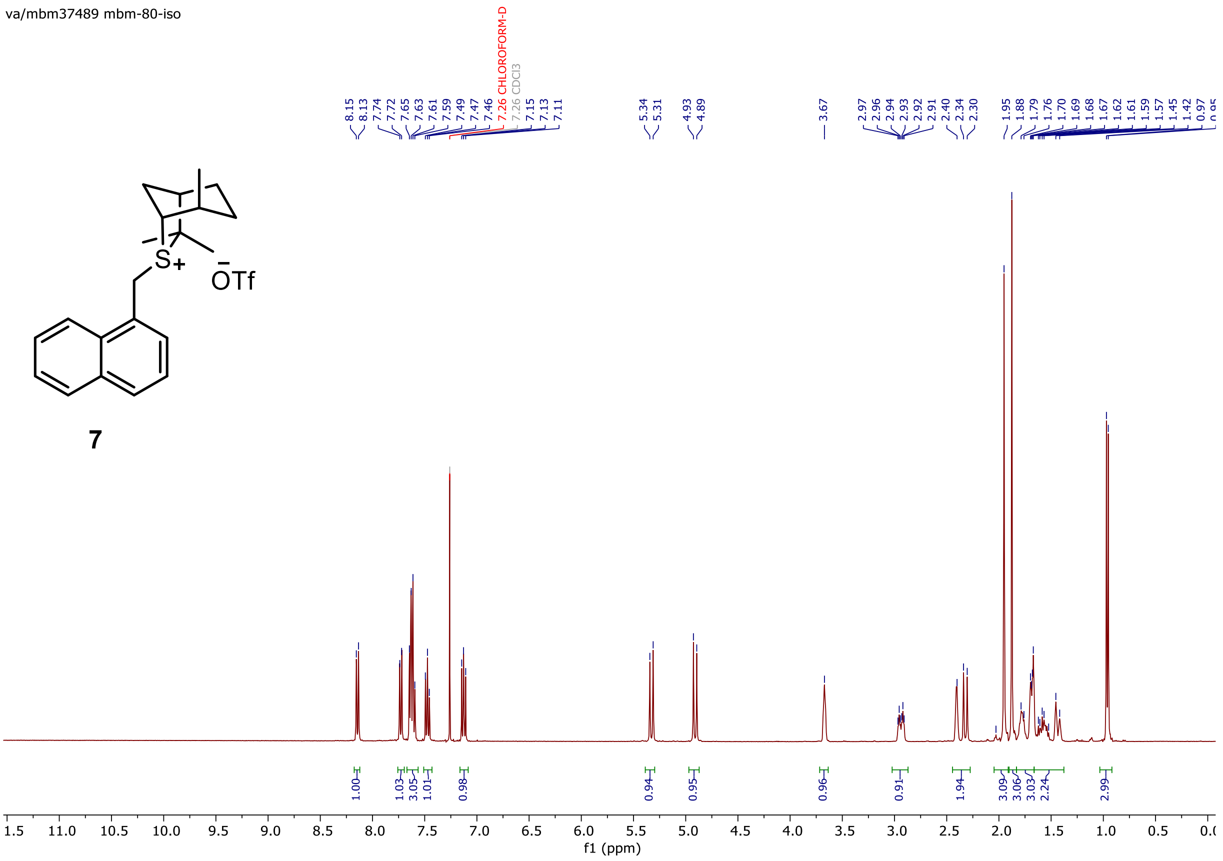

^{13}C NMR (100 MHz, $CDCl_3$) of **7**

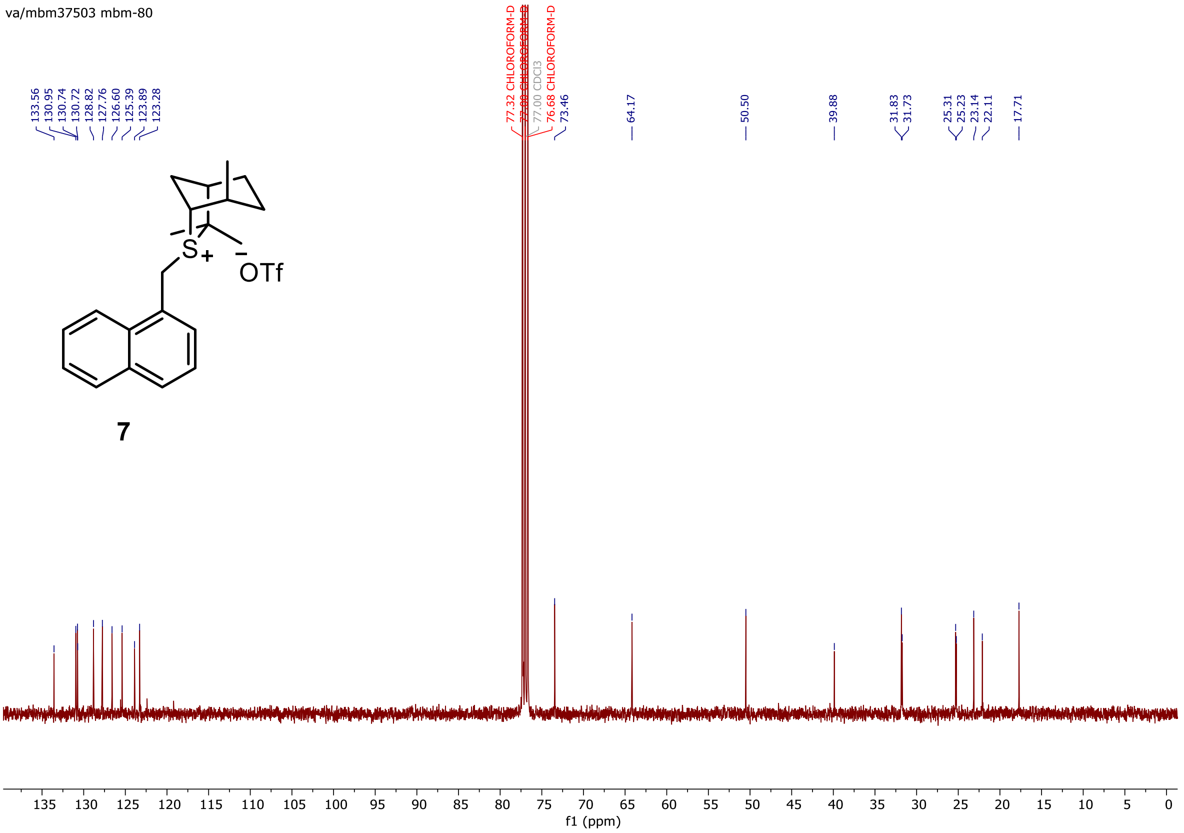

^{1}H NMR (400 MHz, $CDCl_3$) of **13** (see procedure)

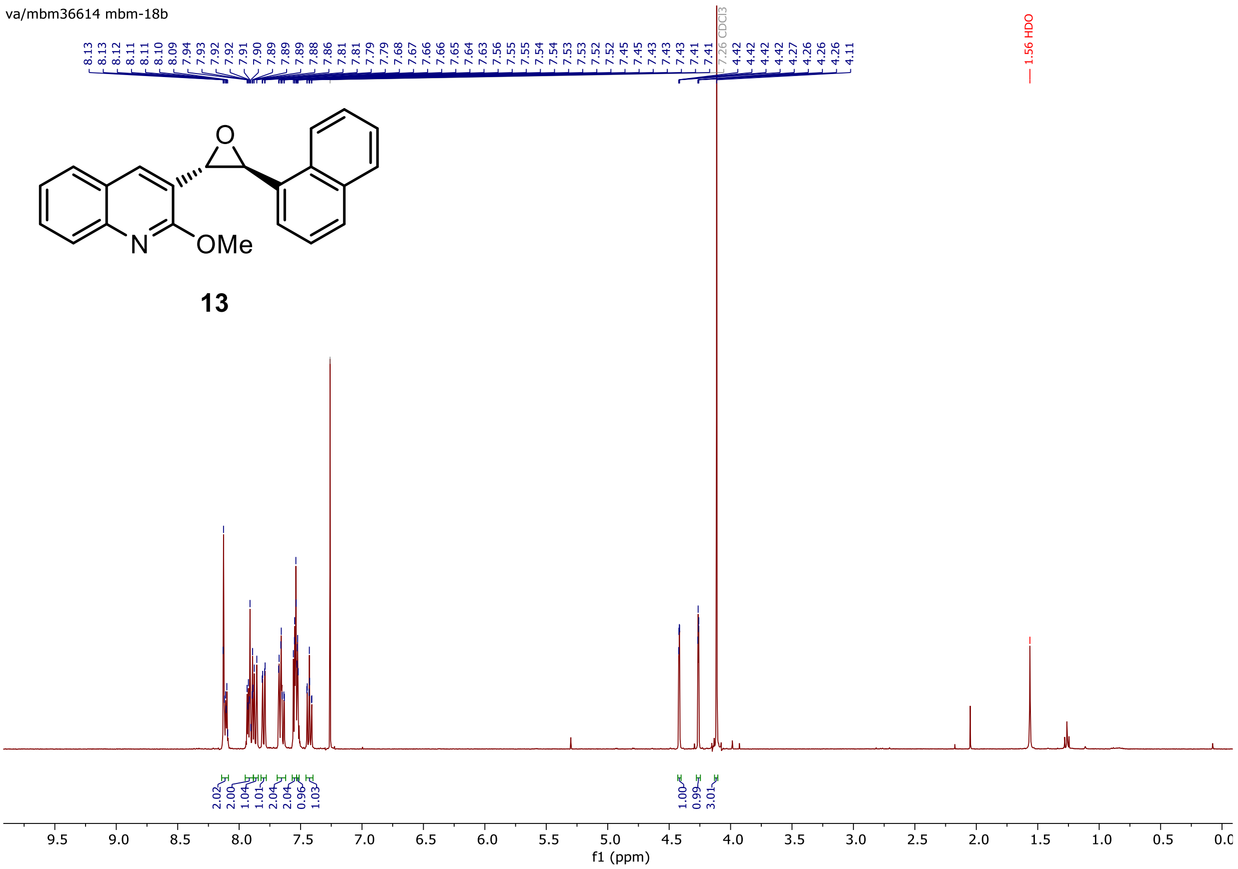

^{13}C NMR (100 MHz, $CDCl_3$) of **13**

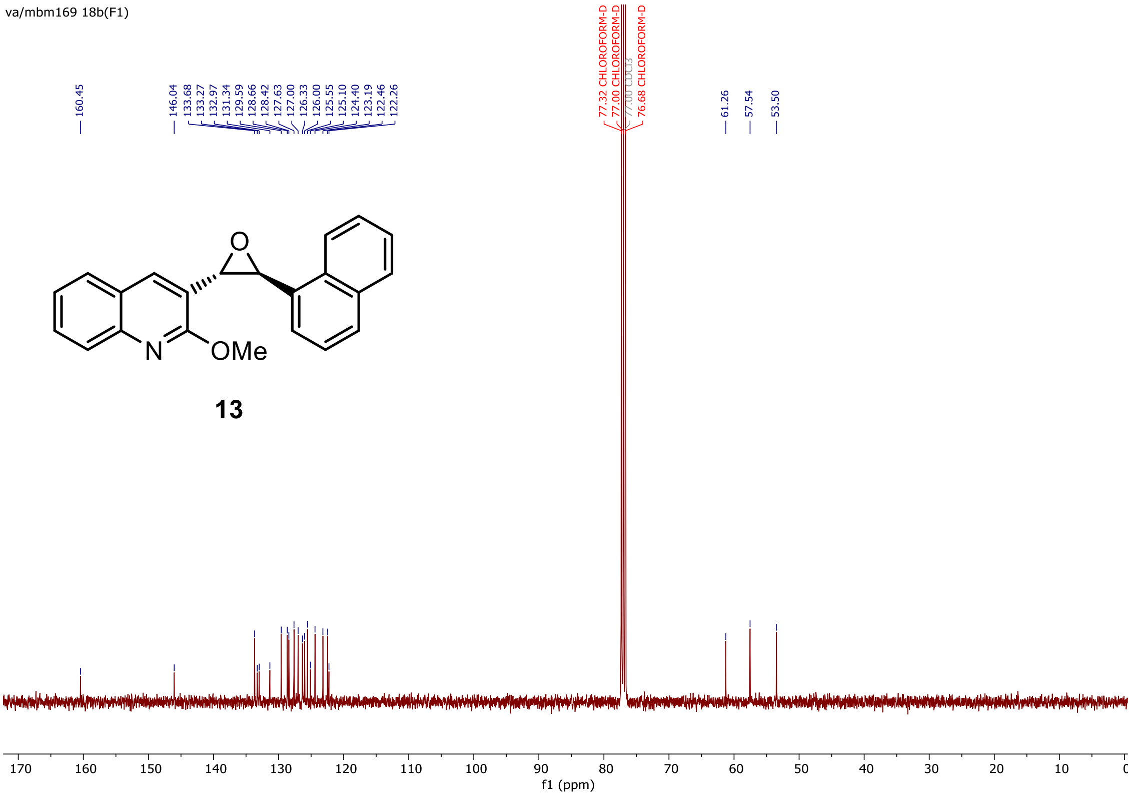

^{1}H NMR (400 MHz, $CDCl_3$) of **(+)-3** (see procedure)

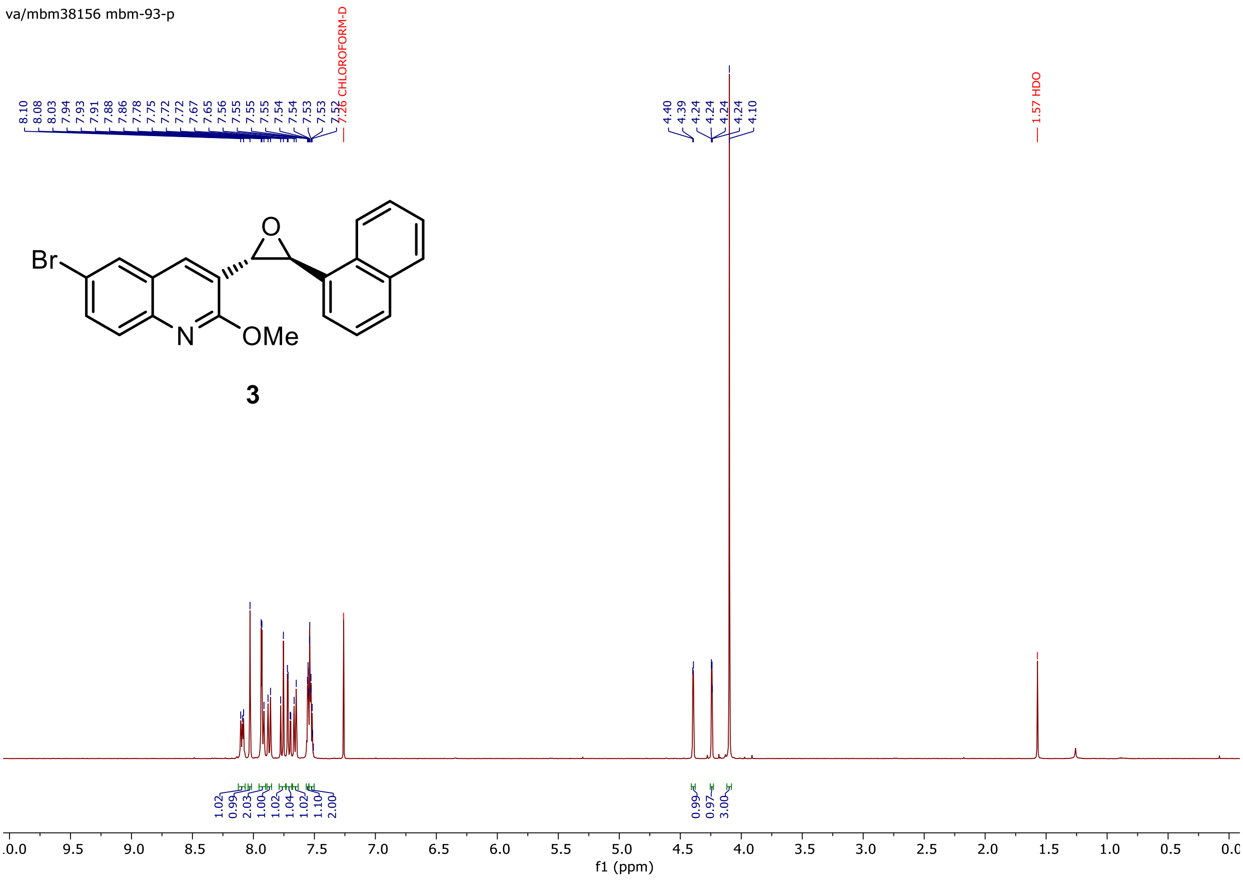

^{13}C NMR (100 MHz, $CDCl_3$) of **(+)-3**

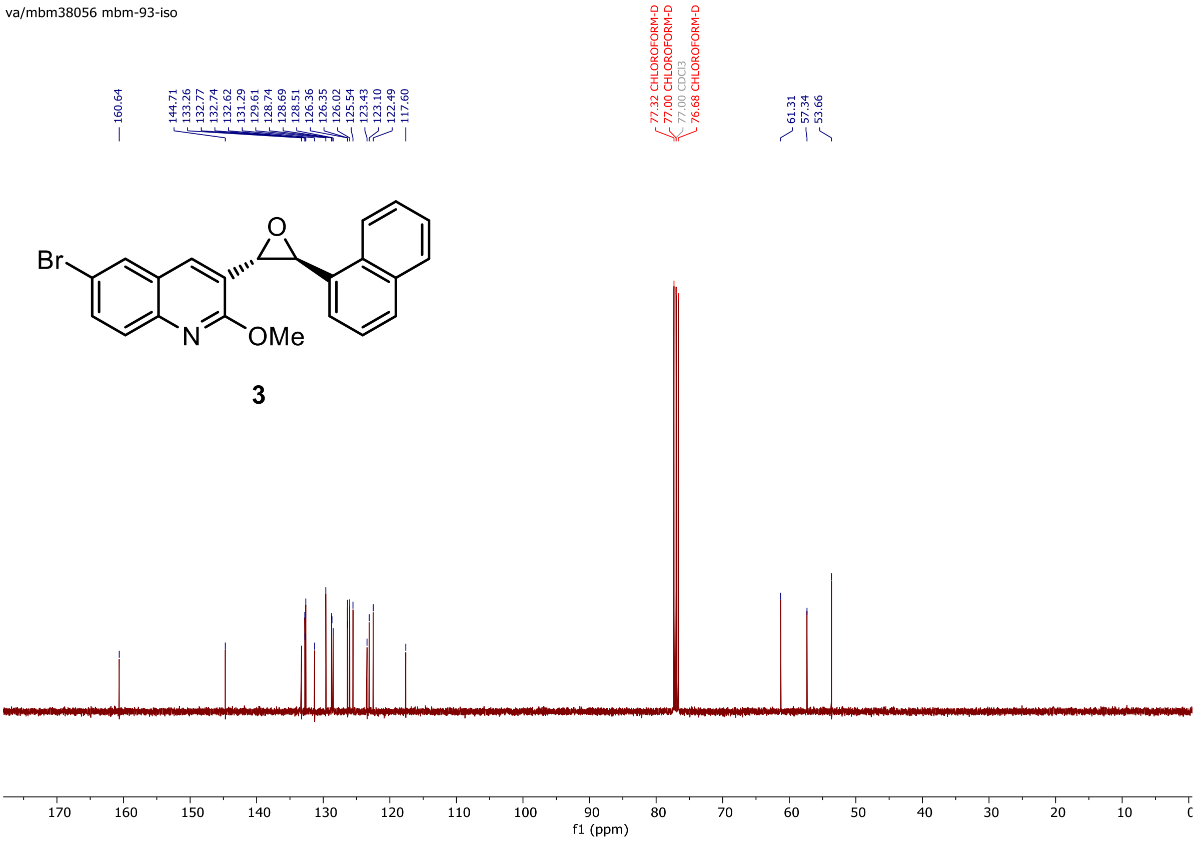

^{1}H NMR (400 MHz, $CDCl_3$) of **14** (see procedure)

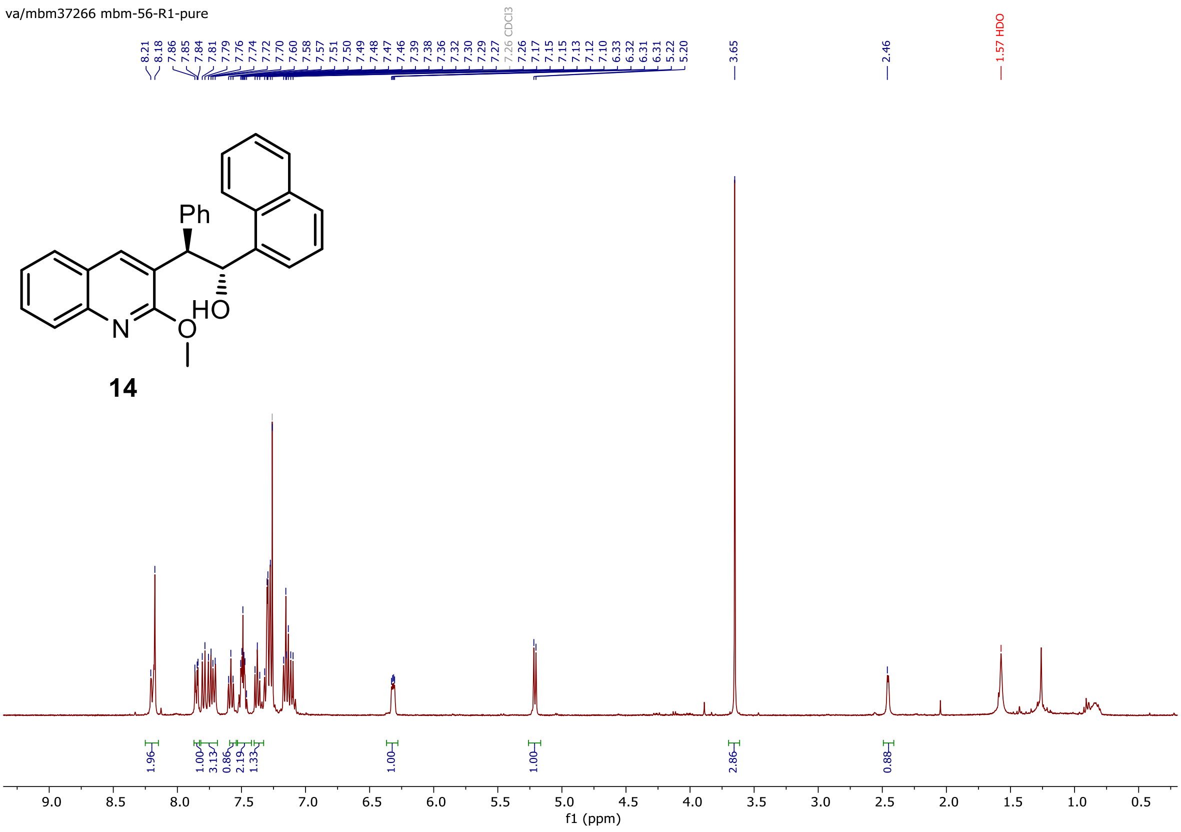

^{13}C NMR (100 MHz, $CDCl_3$) of **14**

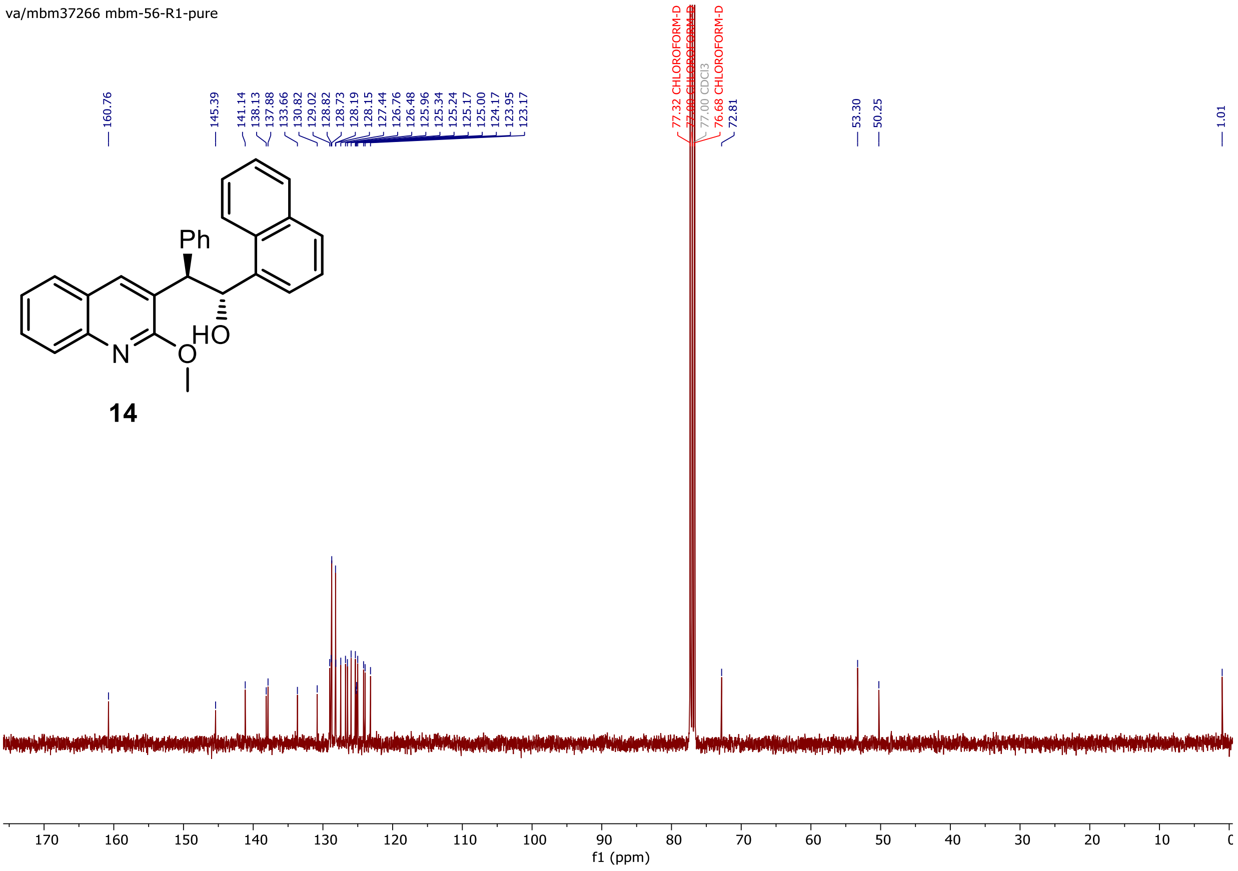

^{1}H NMR (600 MHz, $CDCl_3$) of **15** (see procedure)

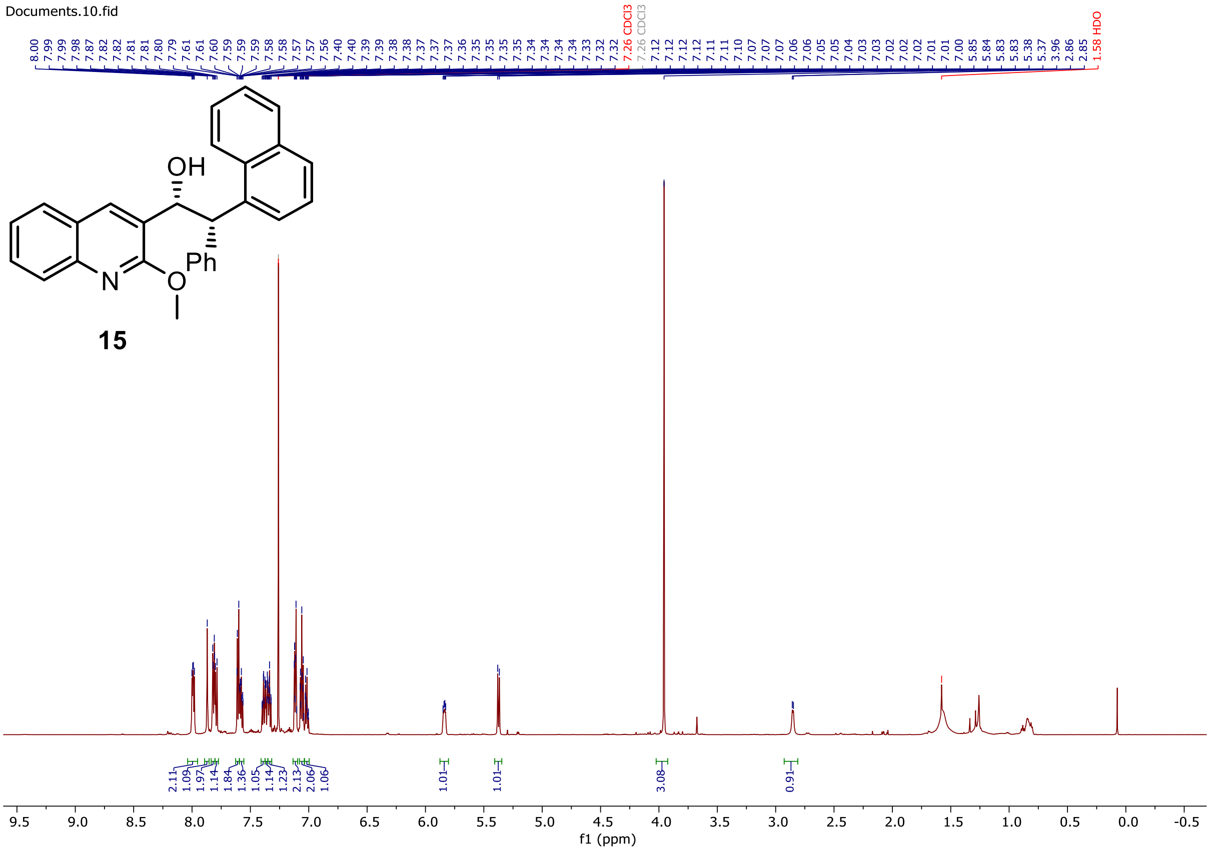

^{13}C NMR (150 MHz, $CDCl_3$) of **15**

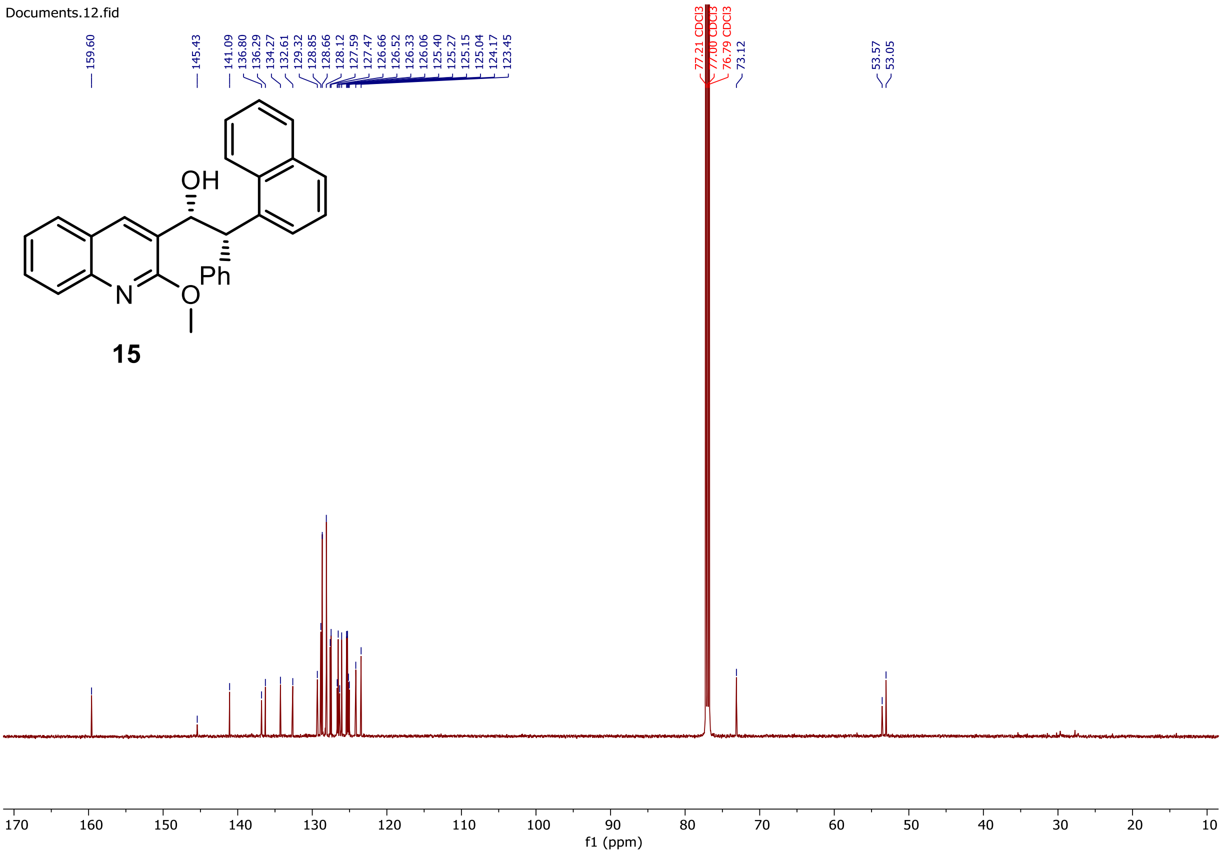

^{1}H NMR (400 MHz, $CDCl_3$) of **(+)-18** (see procedure)

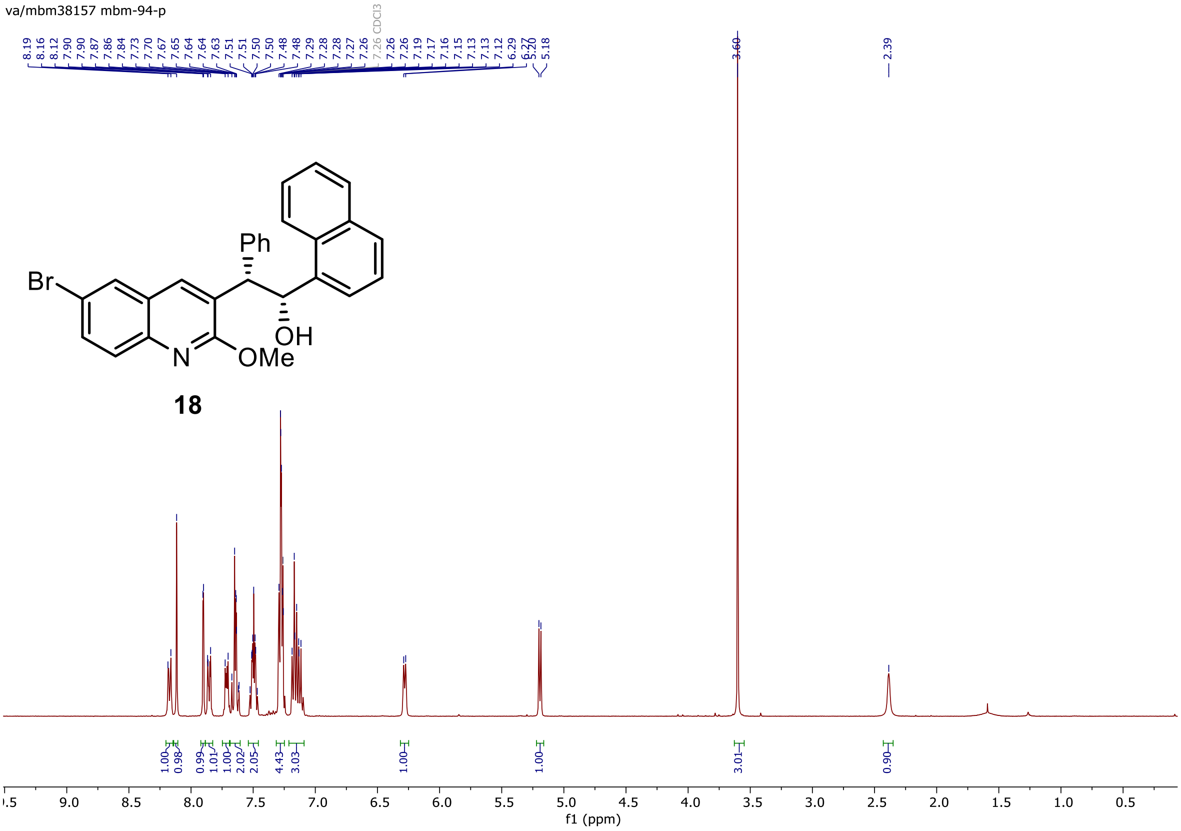

^{13}C NMR (100 MHz, $CDCl_3$) of **(+)-18**

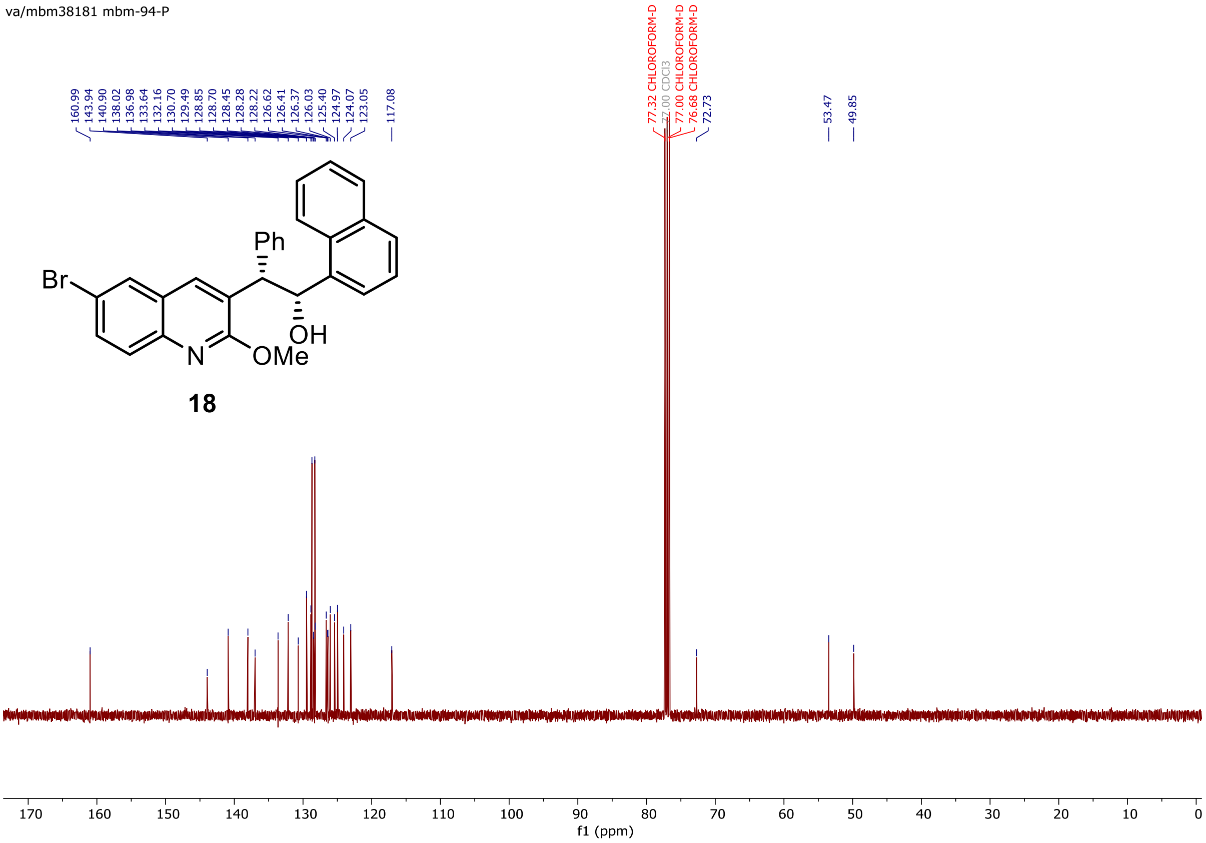

^{1}H NMR (400 MHz, $CDCl_3$) of **(−)-10** (see procedure)

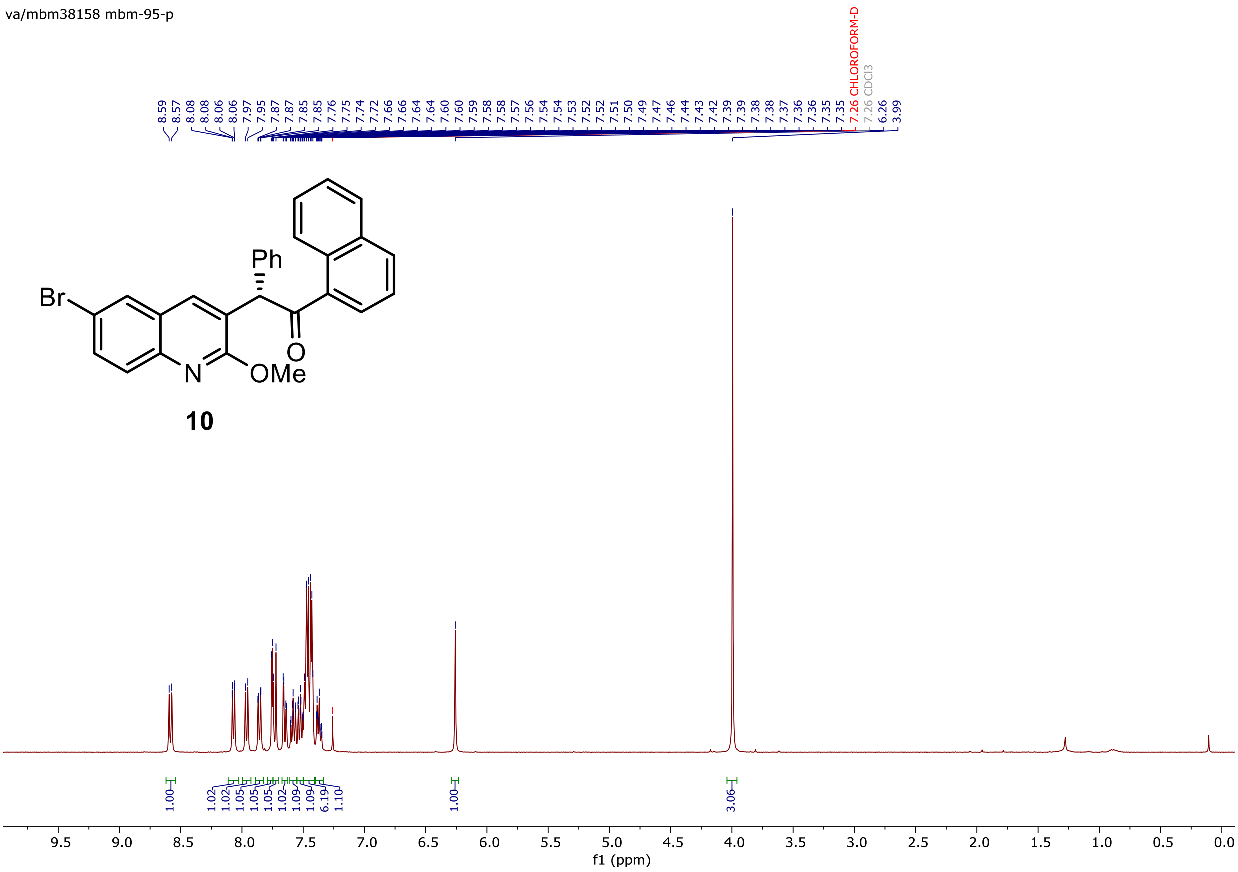

^{13}C NMR (100 MHz, $CDCl_3$) of **(−)-10**

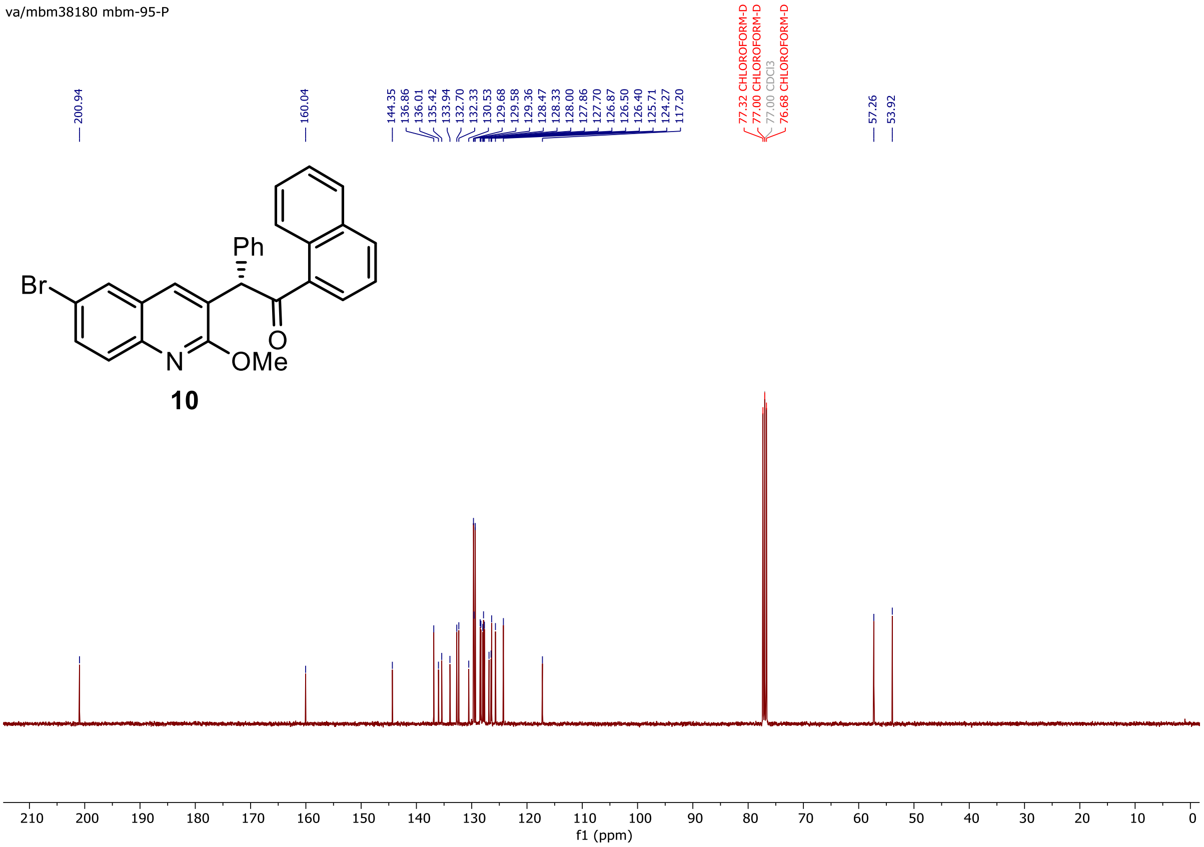

^{1}H NMR (600 MHz, $CDCl_3$) of **(+)-1** (see procedure)

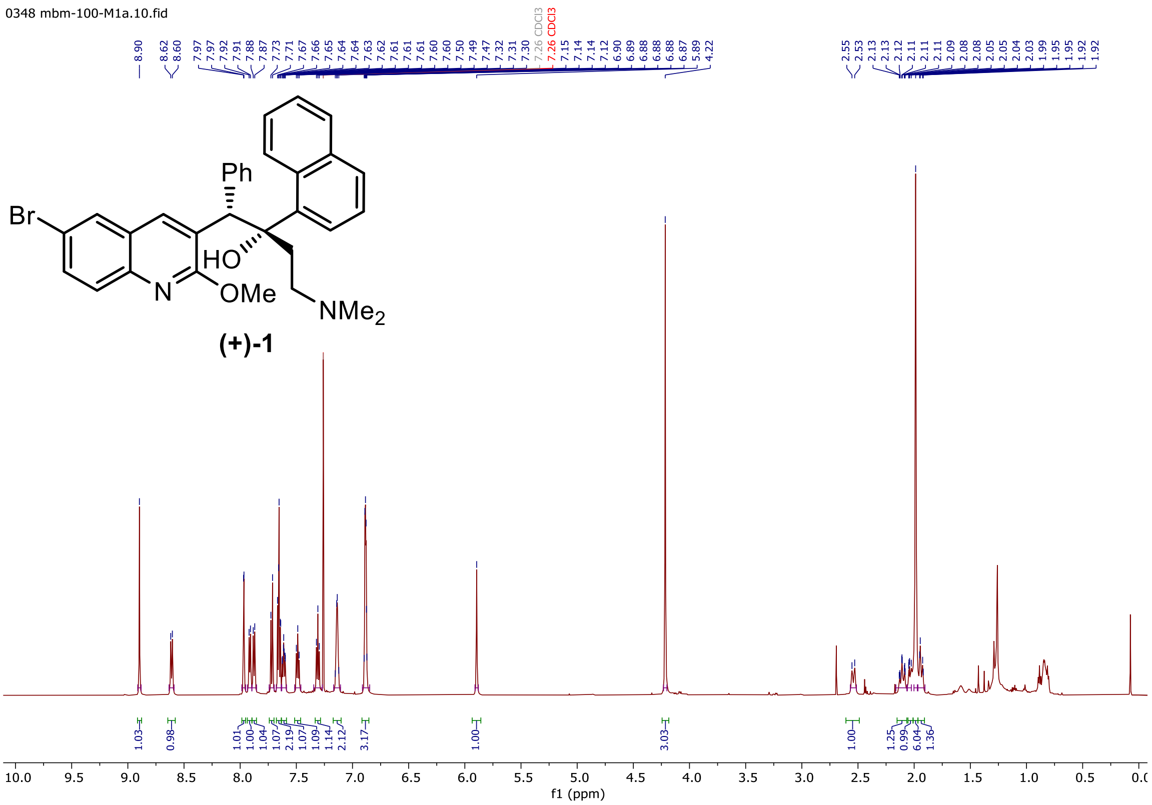

^{13}C NMR (150 MHz, $CDCl_3$) of **(+)-1**

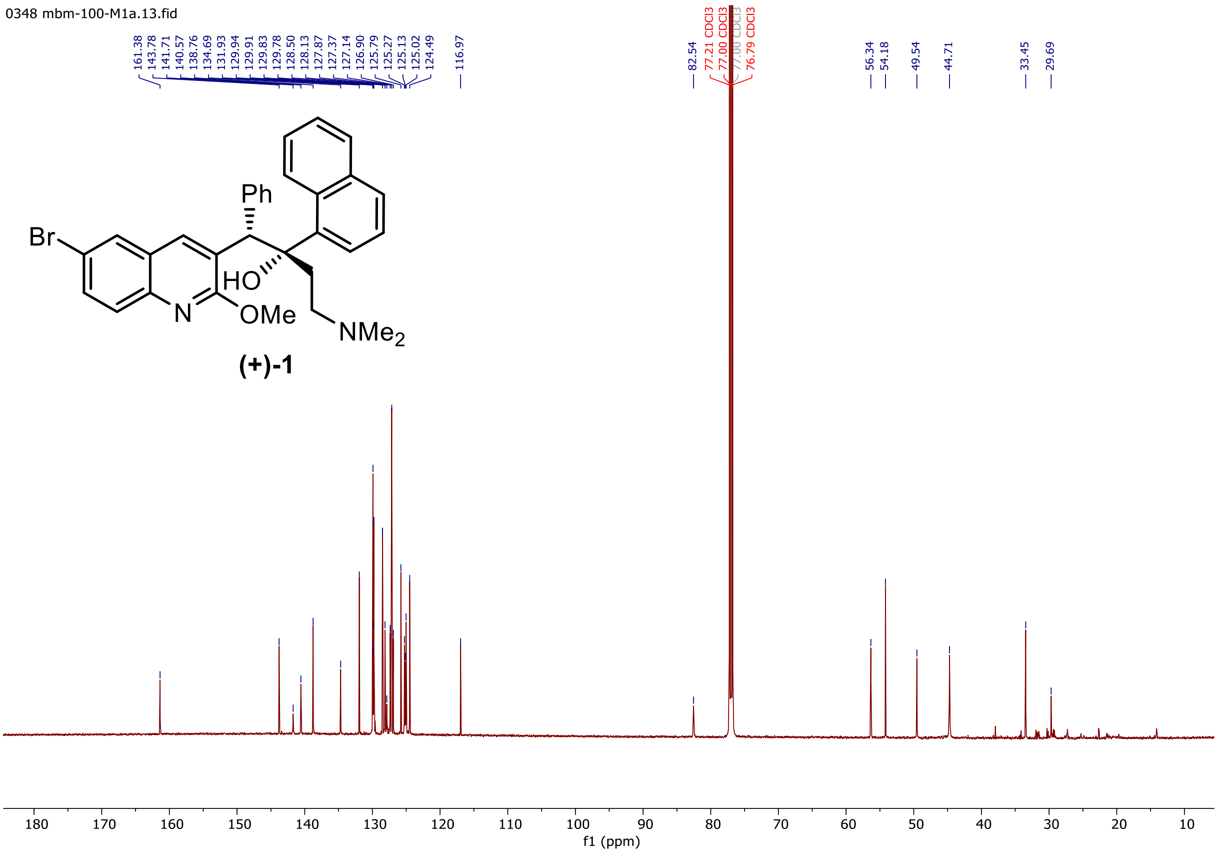

^{1}H NMR (600 MHz, $CDCl_3$) of **21** (see procedure)

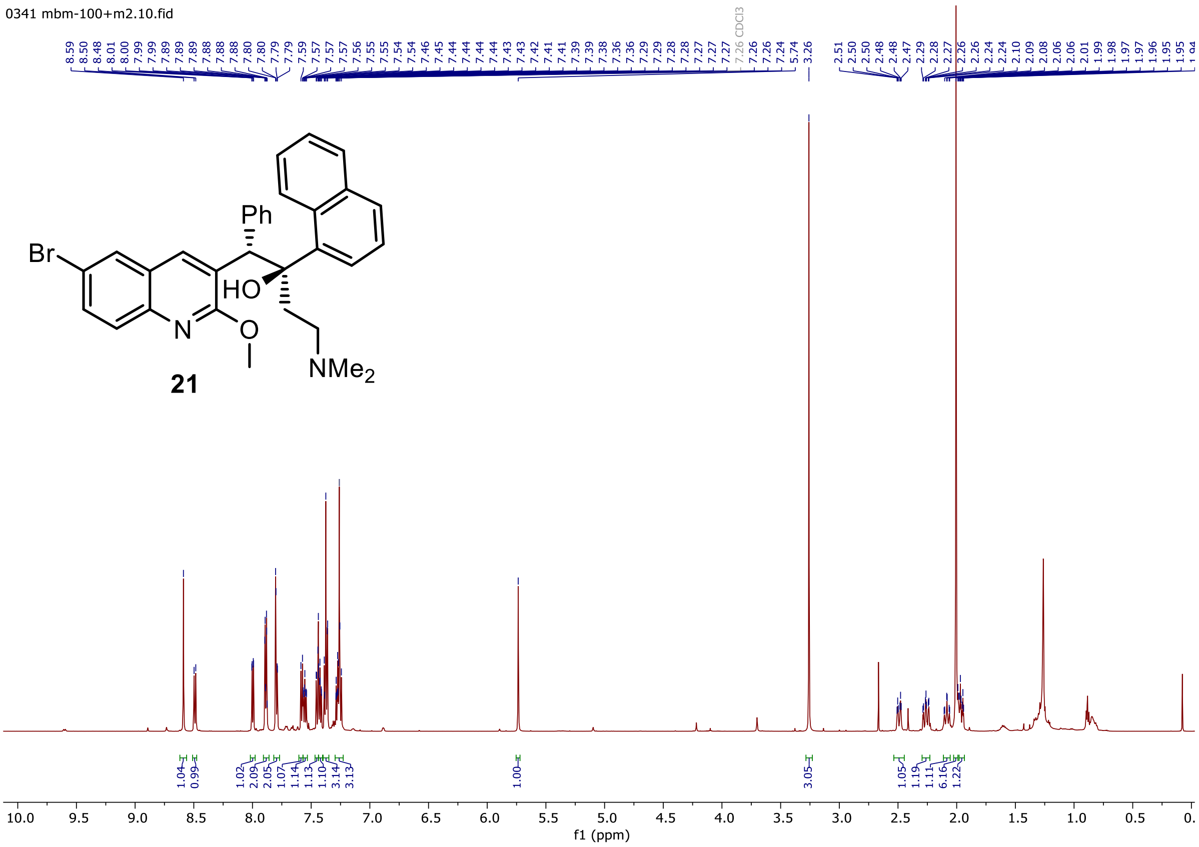

^{13}C NMR (150 MHz, $CDCl_3$) of **21**

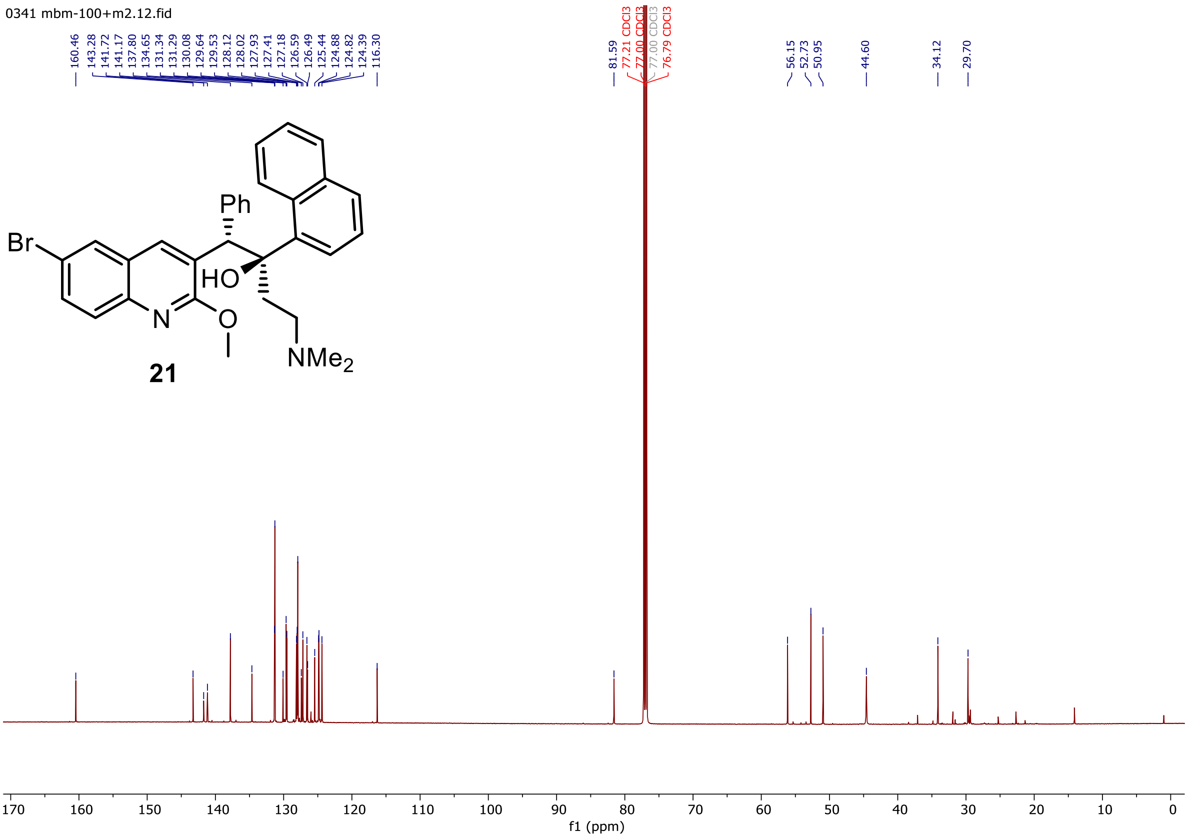

Chiral Phase Chromatograms for Epoxides (+)-3 and (−)-3

Conditions: OD-H column, 1% IPA/Hexane, 1 mL/min, R_t *trans*: 6.60 min. ((*R*,*R*), major), 16.93 min. ((*S*,*S*), minor), *cis:* 3.90 min. and 7.67 min.

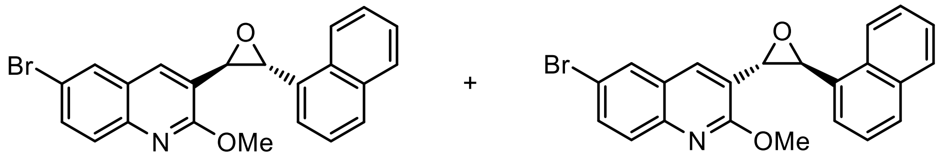

(+)-3 **(–)-3**

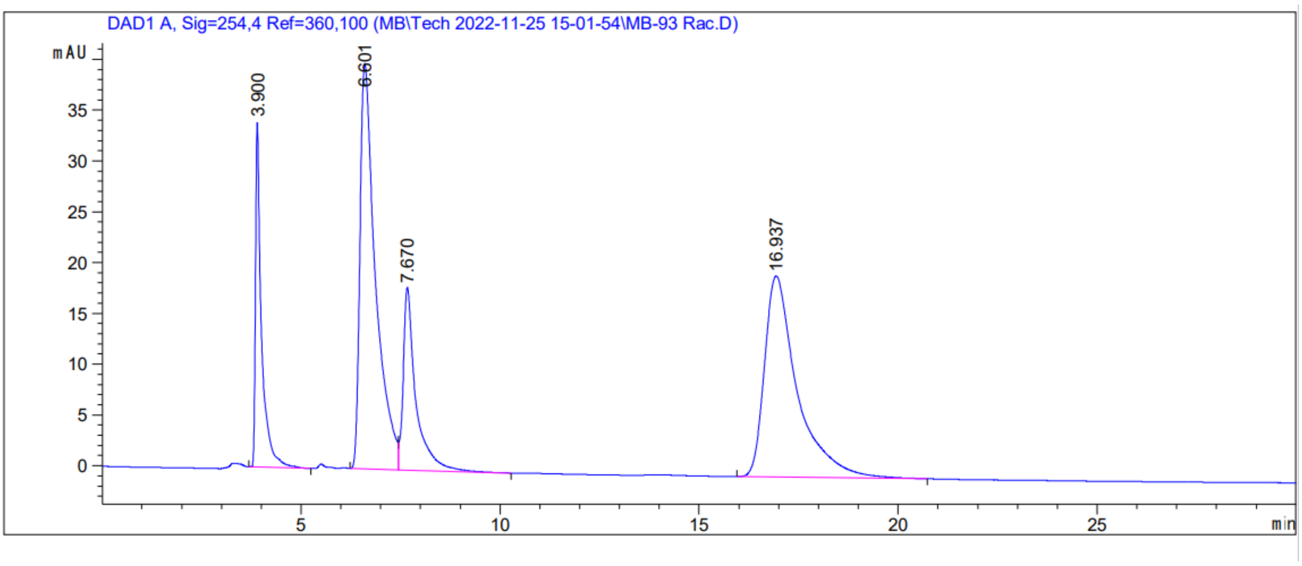

Peak #	RetTime [min]	Type	Width [min]	Area [mAU*s]	Height [mAU]	Area %
1	3.900	BB	0.1431	352.74417	33.98402	11.8750
2	6.601	BV	0.3919	1086.82288	39.88632	36.5874

Peak #	RetTime [min]	Type	Width [min]	Area [mAU*s]	Height [mAU]	Area %
3	7.670	VB	0.3102	398.12845	17.98826	13.4028
4	16.937	BB	0.8379	1132.78870	19.78619	38.1348

Conditions: OD-H column, 1% IPA/Hexane, 1 mL/min, R_t *trans*: 6.60 min. ((*R*,*R*), major), 16.93 min. ((*S*,*S*), minor)

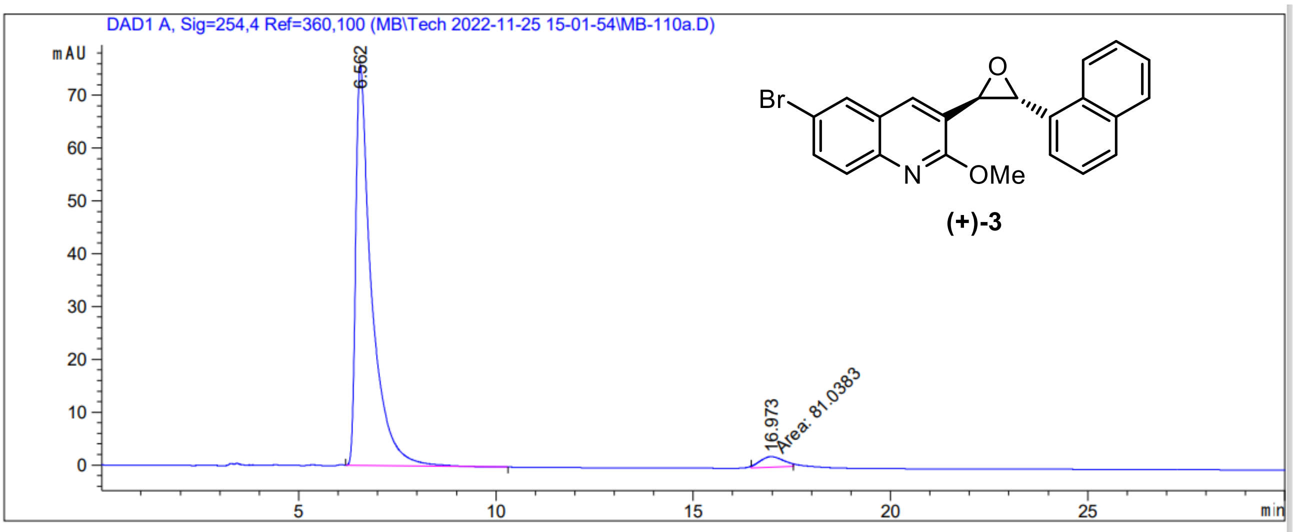

Peak #	RetTime [min]	Type	Width [min]	Area [mAU*s]	Height [mAU]	Area %
1	6.562	BB	0.3978	2117.20142	75.81586	96.3135
2	16.973	MM	0.6831	81.03829	1.97712	3.6865

Conditions: OD-H column, 1% IPA/Hexane, 1 mL/min, R_t *trans*: 6.60 min. ((*R*,*R*), minor), 16.93 min. ((*S*,*S*), major)

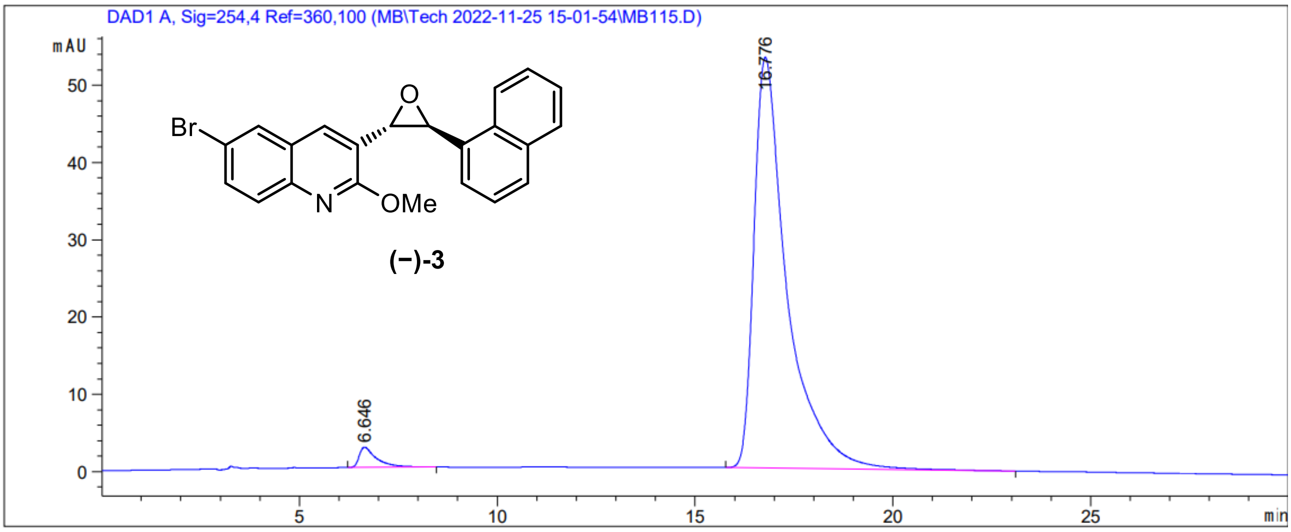

Peak #	RetTime [min]	Type	Width [min]	Area [mAU*s]	Height [mAU]	Area %
1	6.646	BB	0.4342	78.58680	2.58882	2.4727
2	16.776	BB	0.8541	3099.60840	53.17048	97.5273

Comparison of racemic, (+)-3 and (–)-3 enantiomers

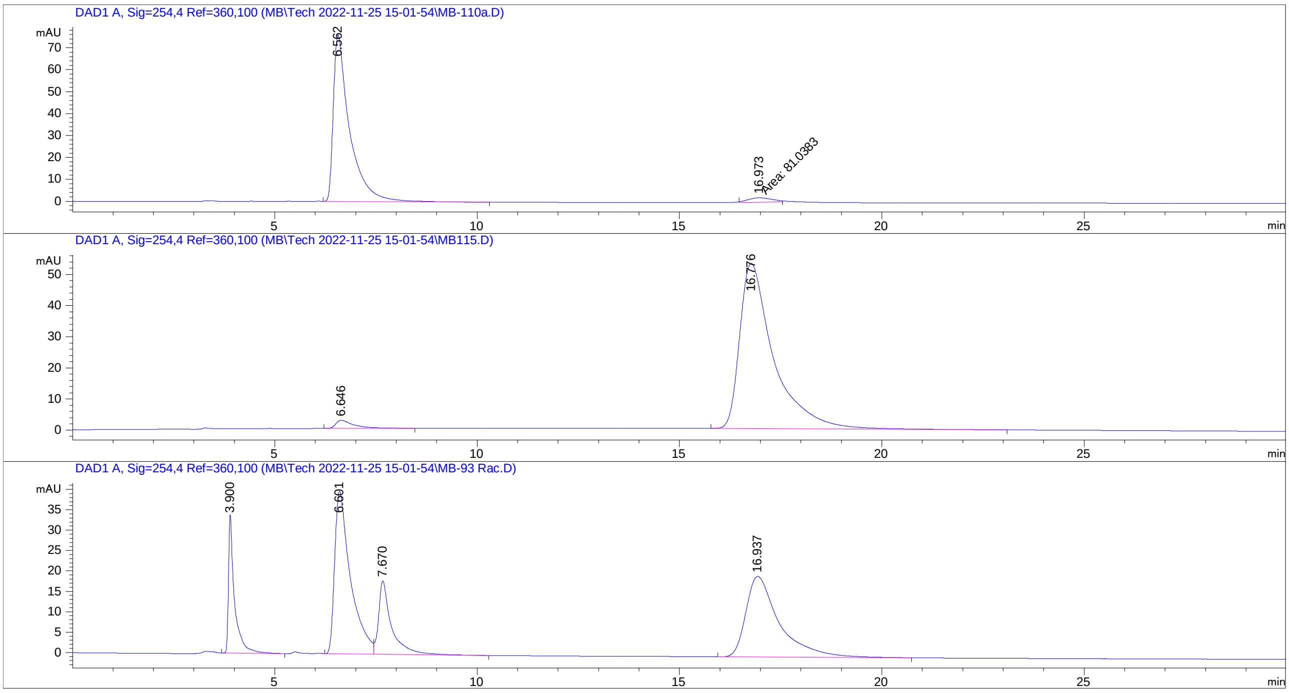

Chiral GC chromatograms

Chiral GC conditions: Oven temperature; On 70 °C for 0 min. then 5 °C/min. to 90 °C for 0 min. then 0.1 °C/min. to 101 °C for 1 min. then 50 °C/min. to 180 °C for 3 min. Run Time 119.58 min.

(+)-Isothiocineole (+)-9 derived from *S*-limonene (*e.r.* 90:10)

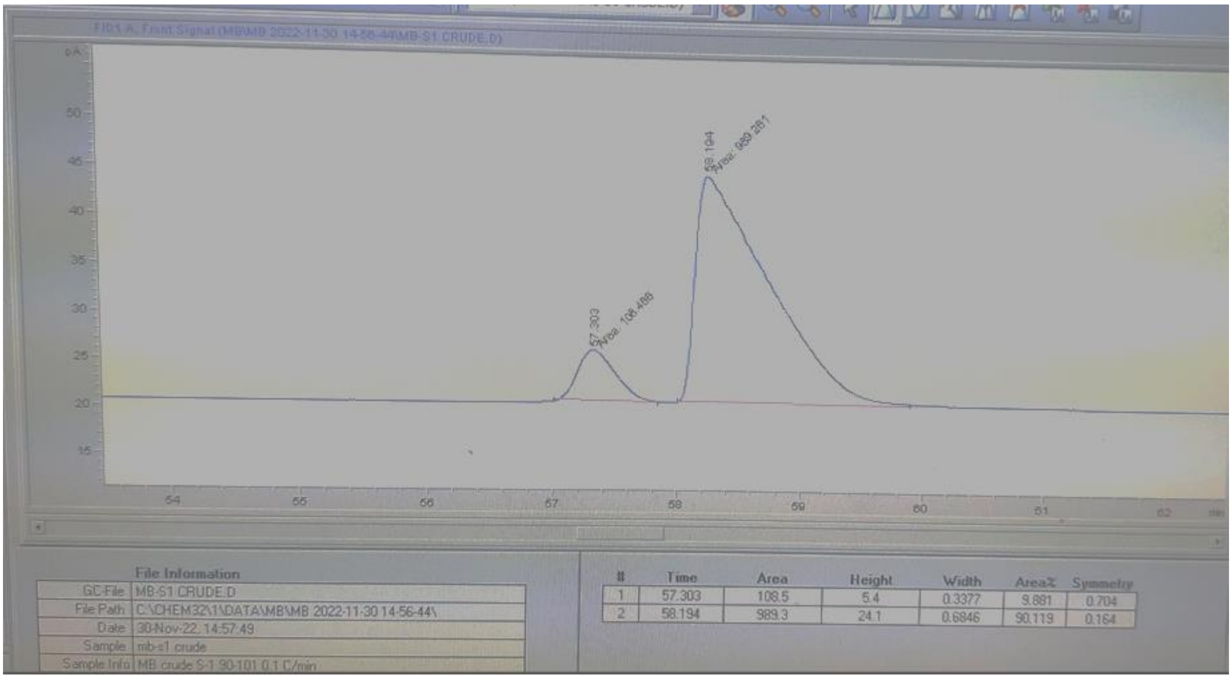

Enantiopure (+)-9 after low temperature (–50 ºC) recrystallization in pentane

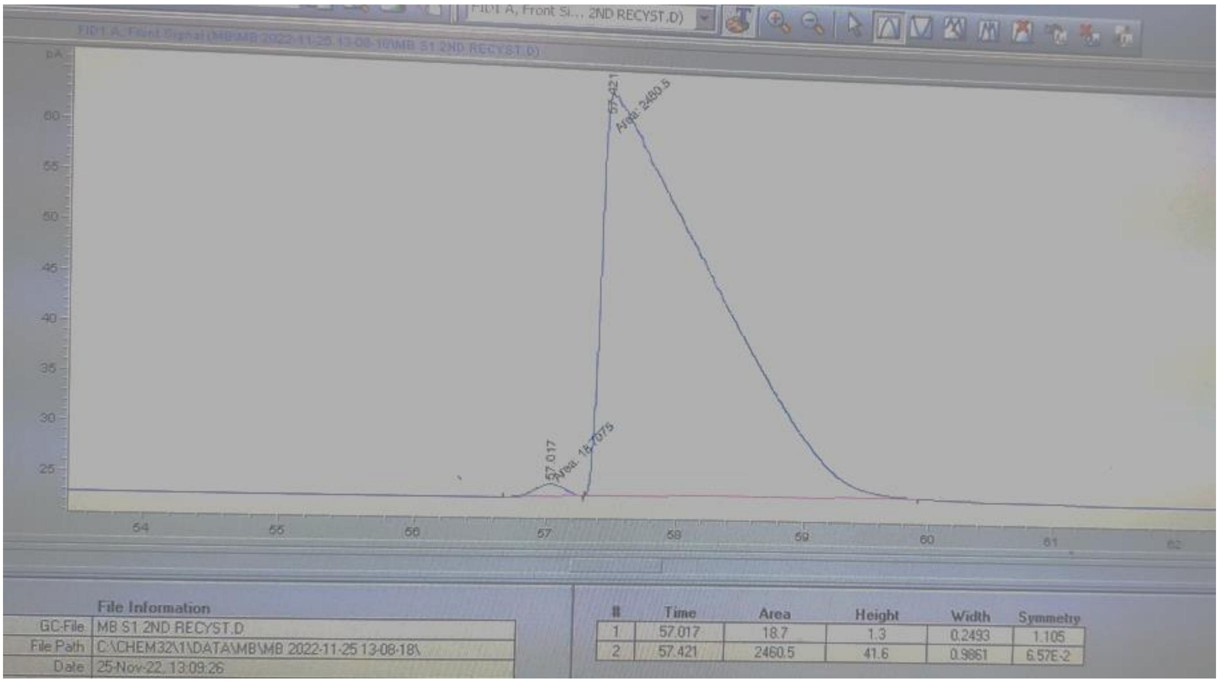

(+)-9 and (−)-9 Isothiocineole mixture

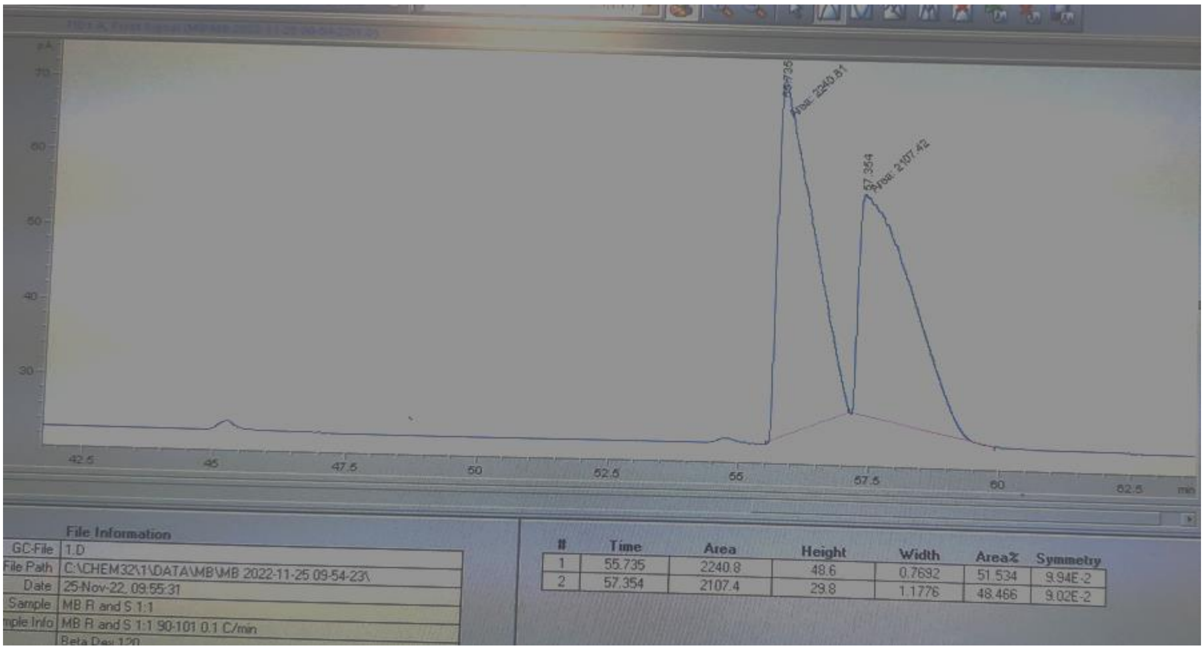

7. REFERENCES

1. Joshi, S. D.; More, U. A.; Parkale, D.; Aminabhavi, T. M.; Gadad, A. K.; Nadagouda, M. N.; Jawarkar, R., *Med. Chem. Res.* **2015,** *24*, 3892-3911.
2. Thangaraj, M.; Gengan, R. M.; Ranjan, B.; Muthusamy, R., *J. Photochem. Photobiol., B,* **2018,** *178*, 287-295.
3. Illa, O.; Namutebi, M.; Saha, C.; Ostovar, M.; Chen, C. C.; Haddow, M. F.; Nocquet-Thibault, S.; Lusi, M.; McGarrigle, E. M.; Aggarwal, V. K., *J. Am. Chem. Soc.* **2013,** *135*, 11951-11966.
4. Illa, O.; Arshad, M.; Ros, A.; McGarrigle, E. M.; Aggarwal, V. K., *J. Am. Chem. Soc.* **2010,** *132*, 1828-1830.
5. Dess, D. B.; Martin, J., *J. Am. Chem. Soc.* **1991,** *113*, 7277-7287.
6. Chandrasekhar, S.; Babu, G. K.; Mohapatra, D. K., *Eur. J. Org. Chem*., **2011**, 2057-2061
7. Barbaro, L.; Nagalingam, G.; Triccas, J. A.; Tan, L.; West, N. P.; Baell, J. B.; Priebbenow, D. L., *RSC Med. Chem.,* **2021,** *12*, 943-959.
8. Mear, S.J.; Lucas, T.; Ahlqvist, G.P.; Robey, J.M.; Dietz, J.P.; Khairnar, P.V.; Maity, S.; Williams, C.L.; Snead, D.R.; Nelson, R.C. and Opatz, T., *Chem. Eur. J.,* **2022**, 28, e2022013.
9. Aggarwal, V. K.; Bae, I.; Lee, H. Y.; Richardson J.; Williams, D. T. *Angew. Chem. Int. Ed.* **2003**, *42*, 3274.
